# Analogous environments across the tropics have similar levels of tree species alpha diversity

**DOI:** 10.1093/nsr/nwaf465

**Published:** 2025-10-29

**Authors:** Shumei Xiao, Jonathan M Adams, Shufeng Li, Ferry Slik, Daniel M Griffith, Adriano Quaresma, Aisha Sultana, Andes Hamuraby Rozak, Andres Avella Muñoz, Andrew R Marshall, Arellano Gabriel, Ashaq Ahmad Dar, Asyraf Mansor, Ayyappan Narayanan, Bruno Herault, Carlos Alfredo Joly, Daniel Piotto, David J Harris, Donald R Drake, Douglas Sheil, Diogo S B Rocha, Eddie Lenza de Oliveira, Eddy Nurtjahya, Eduardo van den Berg, Edward L Webb, Faridah Hanum Ibrahim, Felipe Zamborlini Saiter, Florian Wittmann, Francisco Mora Ardila, Giselda Durigan, Gopal Shukla, Guillermo Ibarra-Manríquez, Hidetoshi Nagamasu, Ida Theilade, Irie Casimir Zo-Bi, Isau Huamantupa-Chuquimaco, J Orlando Rangel-Ch, James Grogan, Javid Ahmad Dar, Jochen Schöngart, John Herbohn, John R Poulsen, John N Williams, Jon Lovett, Jose Don De Alban, José Rafael Lozada, José Roberto Rodrigues Pinto, Juan Ernesto Guevara-Andino, Jurgi Cristóbal-Azkarate, Jürgen Homeier, Katrin Böhning-Gaese, Khalid Rehman Hakeem, Kenneth Feeley, Kyle W Tomlinson, Ladan Rasingam, Layon Oreste Demarchi, Yves Laumonier, Luciana F Alves, Luis Torres Montenegro, Manichanh Satdichanh, Manuel J Macía, Marcelo Tabarelli, Marcio Seiji Suganuma, Marcos Antonio Ríos Paredes, Maria Teresa Fernandez Piedade, Mark Schulze, Mattheas van de Bult, Meredith L Bastian, Mohammed Latif Khan, Mohammad Shah Hussain, Michael Kessler, Michael J Lawes, Miguel A Munguía-Rosas, Narayanaswamy Parthasarathy, Naret Seuaturian, Naveen Babu Kanda, Naveenkumar Jagadeesan, Nigel Pitman, Onrizal Onrizal, Ole R Vetaas, Pantaleo Munishi, Phourin Chhang, Polyanna da Conceição Bispo, Rahmad Zakaria, Rahayu Sukmaria Sukri, Rajkumar Muthu, Rama Chandra Prasad, Ramachandran V S, Rhett D Harrison, Rizza Karen Veridiano, Robert Steinmetz, Robin L Chazdon, Roven Tumaneng, Samir Gonçalves Rolim, S C Garkoti, Selene Báez, Serge Wich, Sharif A Mukul, Shijo Joseph, Simone Aparecida Vieira, S Muthuramkumar, Somaiah Sundarapandian, Sumit Chakravarty, Subashree Kothandaraman, Tânia Wendt, Thiago Metzker, Timothy Whitfeld, Tao Su, Tinde R Van Andel, Verbeeck Hans, Víctor Arroyo-Rodríguez, Wanlop Chutipong, William F Laurance, Yrma Andreina Carrero, Zhekun Zhou

**Affiliations:** Laboratory of Tropical Forest Ecology, Xishuangbanna Tropical Botanical Garden, Chinese Academy of Sciences, Mengla 666303, China; Yunnan Key Laboratory of Forest Ecosystem Stability and Global Change, Xishuangbanna Tropical Botanical Garden, Chinese Academy of Sciences, Mengla 666303, China; University of Chinese Academy of Sciences, Beijing 100049, China; School of Geography and Ocean Science, Nanjing University, Nanjing 210023, China; Laboratory of Tropical Forest Ecology, Xishuangbanna Tropical Botanical Garden, Chinese Academy of Sciences, Mengla 666303, China; Yunnan Key Laboratory of Forest Ecosystem Stability and Global Change, Xishuangbanna Tropical Botanical Garden, Chinese Academy of Sciences, Mengla 666303, China; State Key Laboratory of Plant Diversity and Specialty Crops, Xishuangbanna Tropical Botanical Garden, Chinese Academy of Sciences, Mengla 666303, China; Southeast Asia Biodiversity Research Institute, Chinese Academy of Sciences & Xishuangbanna Tropical Botanical Garden, Chinese Academy of Sciences, Mengla 666303, China; Environmental and Life Sciences, Faculty of Science, University Brunei Darussalam, Gadong BE1410, Brunei Darussalam; Departamento de Ciencias Biológicas y Agropecuarias, EcoSs Lab, Universidad Técnica Particular de Loja, Loja 110150, Ecuador; Grupo de Pesquisa Ecologia, Monitoramento e uso Sustentável de Áreas Úmidas (MAUA), Instituto Nacional de Pesquisas da Amazônia (INPA), Manaus 69067-375, Brazil; Biodiversity Parks Programme, CEMDE, Department of Environmental Studies, University of Delhi, Delhi 110007, India; Research Organization for Life Sciences and Environment, National Research and Innovation Agency (BRIN), Bogor 16911, Indonesia; Facultad del Medio Ambiente y Recursos Naturales, Universidad Distrital Francisco José de Caldas, Bogotá 110231, Colombia; Forest Research Institute, University of the Sunshine Coast, Sippy Downs, QLD 4556, Australia; Reforest Africa, Mang’ula, Kilombero, Tanzania; Flamingo Land Ltd, Kirby Misperton, North Yorkshire, YO17 6UX, UK; Department of Ecology and Evolutionary Biology, University of Michigan, Ann Arbor, MI 48109, USA; Oikobit LLC, Albuquerque, NM 87120, USA; Department of Ecology and Environmental Sciences, School of Life Sciences, Pondicherry University, Puducherry 605014, India; School of Biological Sciences (SBS), Universiti Sains Malaysia, Penang 11800, Malaysia; Centre for Marine and Coastal Studies (CEMACS), Universiti Sains Malaysia, Penang 11800, Malaysia; Department of Ecology, French Institute of Pondicherry, Puducherry 605001, India; CIRAD, Campus de Baillarguet, Montpellier Cedex 5, 34398, France; Department of Plant Biology, Institute of Biology, University of Campinas, Campinas 13083-862, Brazil; Laboratório de Dendrologia e Silvicultura Tropical, Centro de Formação em Ciências Agroflorestais, Universidade Federal do sul da Bahia, Itabuna 45613-204, Brazil; Royal Botanic Garden Edinburgh, Edinburgh EH3 5LR, UK; Harold L. Lyon Arboretum, University of Hawaiʻi at Mānoa, Honolulu, HI 96822, USA; Forest Ecology and Forest Management Group, Wageningen University & Research, Wageningen 6700 AA, The Netherlands; International Institute for Sustainability, Rio de Janeiro 22460-320, Brazil; Programa de Pós-graduação em Ecologia e Conservação, Universidade do Estado de Mato Grosso, Nova Xavantina 78690-000, Brazil; Department of Biology, Universitas Bangka Belitung, Kampus Terpadu UBB Balunijuk, Kec. Merawang, Kab. Bangka, 33172, Indonesia; Herbarium Bangka Belitungense, Kampus Terpadu UBB Balunijuk, Kec. Merawang, Kab. Bangka, 33172, Indonesia; Departamento of Ecology and Conservation, Universidade Federal de Lavras, Lavras, MG 37200-900, Brazil; Viikki Tropical Resources Institute, Department of Forest Sciences, University of Helsinki, Helsinki 00790, Finland; Helsinki Institute of Sustainability Science (HELSUS), Helsinki 00100, Finland; Institute of Tropical Forestry and Forest Products (INTROP), Universiti Putra Malaysia, Serdang, Selangor 43400, Malaysia; Federal Institute of Espirito Santo, Cariacica Campus, Espirito Santo 29150-410, Brazil; National Institute of the Atlantic Forest, Santa Teresa, Espirito Santo 29650-000, Brazil; Department of Wetland Ecology, Karlsruhe 76131, Germany; Instituto de Investigaciones en Ecosistemas y Sustentabilidad, Universidad Nacional Autónoma de México. Morelia 58190, Mexico; Floresta Estadual de Assis, Instituto de Pesquisas Ambientais. Assis 19800-000, Brazil; Department of Forestry, North Eastern Hill University, Tura Campus, Tura, Meghalaya 794002, India; Instituto de Investigaciones en Ecosistemas y Sustentabilidad, Universidad Nacional Autónoma de México. Morelia 58190, Mexico; The Kyoto University Museum, Kyoto University, Kyoto 606-8501, Japan; Department of Food and Resource Economics, University of Copenhagen, DK-1958 Frederiksberg C, Denmark; Institute National Polytechnique Félix Houphouët-Boigny, INP-HB, Yamoussoukro, BP 1093, Côte d'Ivoire; Herbario Alwyn Gentry (HAG), Departamento Académico de Ciencias Básicas. Universidad Nacional Amazónica de Madre de Dios (UNAMAD), Madre de Dios 17001, Peru; Centro Ecológico INKAMAZONIA, Valle de Kosñipata, vía Cusco—Parque Nacional del Manú, Cusco, Peru; Instituto de Ciencias Naturales, Universidad Nacional de Colombia, Bogotá D.C. 111321, Colombia; Botanic Garden of Smith College, Northampton, MA 01063, USA; Department of Environmental Science and Engineering, School of Engineering and Sciences, SRM University-AP, Andhra Pradesh 522240, India; Centre for Geospatial Technology, SRM University-AP, Andhra Pradesh 522240, India; Ecology, Monitoring and Sustainable Use of Wetlands (MAUA), Instituto Nacional de Pesquisas da Amazônia (INPA), Manaus 69060-001, Brazil; Forest Research Institute, University of the Sunshine Coast, Sippy Downs, Queensland 4556, Australia; The Nature Conservancy, Boulder, CO 80302, USA; Department of Environmental Science and Policy, University of California, Davis, CA 95616, USA; School of Geography, University of Leeds, Leeds LS2 9JT, UK; Clear Wind Pte Ltd, Singapore 068894, Singapore; Fauna & Flora International—Philippines Programme, Cambridge CB2 3QZ, UK; Instituto de Investigaciones para el Desarrollo Forestal (INDEFOR), Universidad de Los Andes, Mérida 5101, Venezuela; Departamento de Engenharia Florestal, Centro de Referência em Conservação da Natureza e Recuperação de Áreas Degradadas, Universidade de Brasília, Brasília 70910-900, Brazil; Grupo de Investigación en Ecología y Evolución en los Trópicos-EETrop, Universidad de las Américas, Quito 170125, Ecuador; Department of Basic Psychological Processes and Development, University of the Basque Country UPV/EHU, Donostia 20018, Spain; Faculty of Resource Management, HAWK University of Applied Sciences and Arts, Göttingen 37085, Germany; Senckenberg Biodiversity and Climate Research Centre, Frankfurt am Main, Germany and Goethe University, Frankfurt am Main 60323, Germany; Department of Biological Sciences, Faculty of Science, King Abdulaziz University, Jeddah 21589, Saudi Arabia; Princess Dr. Najla Bint Saud Al-Saud Center for Excellence Research in Biotechnology, King Abdulaziz University, Jeddah 21589, Saudi Arabia; Centre of Research Impact and Outcome, Chitkara University Institute of Engineering and Technology, Chitkara University, Rajpura, Punjab 140401, India; Biology Department, University of Miami, Coral Gables, FL 33146, USA; Center for Integrative Conservation & Yunnan Key Laboratory for Conservation of Tropical Rainforests and Asian Elephants, Xishuangbanna Tropical Botanical Garden, Mengla 666303, China; Botanical Survey of India, Deccan Regional Centre, Hyderabad, Telangana 500095, India; Ecology, Monitoring and Sustainable Use of Wetlands (MAUA), Instituto Nacional de Pesquisas da Amazônia (INPA), Manaus 69060-001, Brazil; Centre for International Forestry Research, Bogor 16115, Indonesia; Institute of the Environment and Sustainability, University of California Los Angeles, Los Angeles, CA 90095, USA; Herbario Herrerense, Instituto de Investigaciones de la Amazonía Peruana, Iquitos 16002, Peru; School of Life Sciences, University of Hawaiʻi at Mānoa, Honolulu, HI 96822, USA; Departamento de Biología, Área de Botánica, Universidad Autónoma de Madrid, Madrid 28049, Spain; Centro de Investigación en Biodiversidad y Cambio Global (CIBC-UAM), Universidad Autónoma de Madrid, Madrid 28049, Spain; Botany Department, Universidade Federal de Pernambuco, Recife, PE 50670-901, Brazil; Colégio de Aplicação, Universidade Federal de Santa Catarina, Florianópolis 88040-900, Brazil; Herbario Herrerense, Instituto de Investigaciones de la Amazonía Peruana, Iquitos 16002, Peru; Keller Science Action Center, The Field Museum, Chicago, IL 60605-2496, USA; Ecology, Monitoring and Sustainable Use of Wetlands (MAUA), Instituto Nacional de Pesquisas da Amazônia (INPA), Manaus 69060-001, Brazil; H.J. Andrews Experimental Forest, Blue River, OR 97413, USA; Doi Tung Development Project, Social Development Department, Chiang Rai 57240, Thailand; Proceedings of the National Academy of Sciences, Washington, DC 20001, USA; Department of Evolutionary Anthropology, Duke University, Durham, NC 27708, USA; Department of Botany, Dr. Harisingh Gour Vishwavidyalaya (A Central University), Sagar, Madhya Pradesh 470003, India; Biodiversity Parks Programme, CEMDE, Department of Environmental Studies, University of Delhi, Delhi 110007, India; Systematic and Evolutionary Botany, University of Zurich, Zurich 8008, Switzerland; School of Life Sciences, University of KwaZulu-Natal, Scottsville 3209, South Africa; Laboratorio de Ecología Terrestre, Centro de Investigación y de Estudios Avanzados del Instituto Politécnico Nacional (Cinvestav), Mérida 97310, Mexico; Department of Ecology and Environmental Sciences, School of Life Sciences, Pondicherry University, Puducherry 605014, India; World Wildlife Fund Thailand, Bangkok 10400, Thailand; Department of Ecology and Environmental Sciences, School of Life Sciences, Pondicherry University, Puducherry 605014, India; Department of Ecology, French Institute of Pondicherry, Puducherry 605001, India; National Biobank of Thailand, National Science and Technology Development Agency, Pathum Thani 12120, Thailand; Viikki Tropical Resources Institute, Department of Forest Sciences, University of Helsinki, Helsinki 00790, Finland; Field Museum of Natural History, Chicago, IL 60605, USA; Faculty of Forestry, Universitas Sumatera Utara, Medan 20155, Indonesia; Department of Geography, University of Bergen, Bergen N-5020, Norway; Department of Ecosystems and Conservation, Sokoine University of Agriculture, Morogoro Tanzania; Forest and Wildlife Research and Development Institute, Phnom Penh, Cambodia; Department of Geography, School of Environment Education and Development, University of Manchester, Manchester M13 9PL, UK; School of Biological Sciences, Universiti Sains Malaysia, Pulau Pinang 11800, Malaysia; Institute for Biodiversity and Environmental Research, Universiti Brunei Darussalam, Jalan Tungku Link, BE 1410, Brunei Darussalam; Tropical Forest Research Institute, Jabalpur, Madhya Pradesh 482021, India; Lab for Spatial Informatics, International Institute of Information Technology, Telangana 500032, India; Centre for Environmental Studies, Amrita School of Engineering, Coimbatore, Amrita Vishwa Vidyapeetham 641112, India; CIFOR-ICRAF, St Eugene Office Park, Lusaka, Zambia; FORLIANCE GmbH, Bonn 53119, Germany; Fauna & Flora International—Philippines Programme, Cambridge CB2 3QZ, UK; World Wildlife Fund Thailand, Bangkok 10400, Thailand; Forest Research Institute, University of the Sunshine Coast, Sippy Downs, QLD, 4556, Australia; Fauna & Flora International—Philippines Programme, Cambridge CB2 3QZ, UK; Department of Science and Technology—Philippine Council for Industry, Energy and Emerging Technology Research and Development, Taguig City 1631, Philippines; Laboratório de Dendrologia e Silvicultura Tropical, Centro de Formação em Ciências Agroflorestais, Universidade Federal do sul da Bahia, Itabuna 45613-204, Brazil; School of Environmental Sciences, Jawaharlal Nehru University, New Delhi 110067, India; Department of Biology, Faculty of Sciences, National Polytechnic School of Ecuador, Quito 170525, Ecuador; School of Biological and Environmental Sciences, Liverpool John Moores University, Liverpool L3 3AF, UK; Department of Environment and Development Studies, United International University, Dhaka 1212, Bangladesh; Tropical Forests and People Research Centre, University of the Sunshine Coast, Maroochydore DC, Queensland 4558, Australia; Department of Climate Variability and Aquatic Ecosystems, Faculty of Ocean Science and Technology, Kerala University of Fisheries and Ocean Studies, Kerala 692508, India; Environmental Studies and Research Center, Universidade Estadual de Campinas, UNICAMP, Campinas 13083-970, Brazil; Research Centre in Botany, VHNSN College, Virudhunagar 626001, India; Department of Ecology and Environmental Sciences, School of Life Sciences, Pondicherry University, Puducherry 605014, India; Department of Forestry, Uttar Banga Krishi Viswavidyalaya, Pundibari, Cooch Behar, West Bengal 736165, India; Department of Environmental Science and Engineering, School of Engineering and Sciences, SRM University-AP, Andhra Pradesh 522240, India; Centre for Geospatial Technology, SRM University-AP, Andhra Pradesh 522240, India; Department of Botany, Institute of Biology, Federal University of Rio de Janeiro, Rio de Janeiro 21941-590, Brazil; IBAM—Instituto Bem Ambiental, Belo Horizonte 30130-090, Brazil; Myr Projetos Sustentáveis (Grupo Myr), Belo Horizonte 30130-009, Brazil; Bell Museum, University of Minnesota, St Paul, MN 55108, USA; Laboratory of Tropical Forest Ecology, Xishuangbanna Tropical Botanical Garden, Chinese Academy of Sciences, Mengla 666303, China; Yunnan Key Laboratory of Forest Ecosystem Stability and Global Change, Xishuangbanna Tropical Botanical Garden, Chinese Academy of Sciences, Mengla 666303, China; State Key Laboratory of Oil and Gas Reservoir Geology and Exploitation & Institute of Sedimentary Geology, Chengdu University of Technology, Chengdu 610059, China; Naturalis Biodiversity Center, 2300 RA, Leiden, The Netherlands; CAVElab -Computational & Applied Vegetation Ecology, Department of Environment, Faculty of Bioscience Engineering, Ghent University, 9000 Gent, Belgium; Instituto de Investigaciones en Ecosistemas y Sustentabilidad, Universidad Nacional Autónoma de México. Morelia 58190, Mexico; Escuela Nacional de Estudios Superiores, Universidad Nacional Autónoma de México, Mérida, 97357, Mexico; Conservation Ecology Program, King Mongkut’s University of Technology Thonburi, Bangkok 10150, Thailand; Centre for Tropical Environmental and Sustainability Science, and College of Science and Engineering, James Cook University, Cairns, Queensland 4878, Australia; Programa de Maestría en Manejo de Bosques, Universidad de Los Andes, Vía Chorros de Milla, Mérida 5101, Venezuela; Laboratory of Tropical Forest Ecology, Xishuangbanna Tropical Botanical Garden, Chinese Academy of Sciences, Mengla 666303, China; Yunnan Key Laboratory of Forest Ecosystem Stability and Global Change, Xishuangbanna Tropical Botanical Garden, Chinese Academy of Sciences, Mengla 666303, China

**Keywords:** rainforest, tree richness, modeling, climate, sample survey

## Abstract

Different regions of the tropics vary in overall tree species diversity, with the tropical Americas exhibiting strikingly higher regional tree species richness than Africa and Southeast Asia. We investigated whether these differences also occur at the local scale and whether the environmental conditions associated with tree species richness are consistent across tropical regions despite highly dissimilar species pools. A spatial random forest model was trained by using a network of 429 1-hectare plots across the tropics, together with 24 environmental variables, to predict plot-level tree α diversity. A combination of climatic, soil and topographical variables explained ∼86% of the variation in richness. Despite differences in regional species pools and the potentially disruptive effects of different geological, climatic and evolutionary histories, the relationship between environmental variables and local-scale tree species richness is closely similar across different continents. Our findings imply a pervasive role of niche-based mechanisms in structuring local tree species richness, regardless of the regional species assemblages. This pantropical convergence in the richness–environment relationship poses a challenge for ecology to explain.

## INTRODUCTION

High levels of tree species richness and diversity in tropical forests have long fascinated biologists, representing an enduring challenge to ecological theory. While many mechanisms have been proposed to explain how such high levels of diversity have arisen and are maintained, substantial uncertainty persists [1–4]. To test emerging hypotheses about the mechanisms underlying tropical tree diversity, an empirical approach is required to identify robust predictors of species richness variation across broad scales [5]. An ideal method to explore and explain patterns of tropical tree diversity is to compare standardized inventory plots [1]. In a pioneering study, Gentry [1] compared a relatively limited number of plots by using least-squares regression to reveal a general relationship between primary productivity and species richness. Other studies of woody plant species richness, reviewed in [6], have concentrated on analysing single regions separately or are extratropical, preventing the comparison of tropical tree diversity relationships between regions. Ricklefs and He [7] compared 47 forest plots globally, fewer than half of which were from the tropics, finding that consistently warm and moist climates favored higher richness. However, they detected significant regional variation in extratropical regions and the limited number of plots prevented definitive comparison within the tropics.

Consideration of the large differences in geological and evolutionary history between the different regions and subregions of tropical forest led us to propose two main hypotheses: (i) local-scale tree α diversity across the global tropics can be consistently predicted by using contemporary environmental variables, such that analogous environmental conditions will support similar levels of richness, irrespective of deep historical biogeographic divergences; (ii) due to localized effects of forest history and dispersal lag, and the complex ecology of forest communities, there will be differences in the best predictors of 1-ha richness at the local scale compared with at the broader scale.

While more recent studies have expanded upon this foundation, pantropical comparisons of the patterns and potential drivers of local tree diversity remain limited. Comparing 2046 tree plots across Amazonia, Ter Steege *et al.* [8] demonstrated that a combined influence of climate and soil factors explained local tree richness and community composition. In a comparison of forest plots from South America and Africa, Parmentier *et al.* [9] showed lower plot-level richness in Africa under similar warm and moist climatic conditions. In global surveys of forest plots of varying sizes, Keil and Chase [10] and Chu *et al.* [11] found that the drivers of the variation in diversity differ with the plot size and spatial distance between samples, which are important sampling issues in ecology generally [12]. However, no studies thus far have addressed local tree species richness by using a large number of plots of standardized size across the world’s tropics, which is necessary for continental-scale comparisons. The increasing availability and integration of forest plot data now make such comparisons possible [13].

Two other important advances in environmental data science facilitate hypothesis testing about the patterns and processes driving tropical tree diversity. First, the availability of interpolated climate, soil and other environmental parameters has grown immensely in recent years [14–18]. Second, machine-learning models enable analysis of the simultaneous effects of multiple factors on community structure and diversity across large scales [19,20]. A particularly robust machine-learning method is random forest (RF) modeling, which, when combined with spatial regression, enables the analysis and prediction of spatially structured data in which observations exhibit autocorrelation [21,22]. These models extend the capabilities of traditional RF to handle spatial data more effectively, allowing spatial autocorrelation and other geographic phenomena to be incorporated into vegetation modeling.

Here, we used the Pantropical Forests Network (PFN) of tree inventory plots assembled by Slik *et al.* [23] in combination with publicly available environmental data surfaces [14–18] to determine which environmental variables explain plot-level tree α diversity across the global tropics. We applied a spatial RF and negative binomial generalized linear model to detect and compare the strength of empirical links between environmental variables and tree species richness. The number of species per unit area (e.g. Gentry [1])—the classic measure of forest tree richness—has its weaknesses given that stem density varies by both biogeographic region and along climate gradients. To compare species richness while accounting for variations in sampling effort and completeness, we employed rarefaction, which is a sample-coverage-based approach based on abundance data (e.g. [24,25]), together with Fisher’s α [26], and the ‘classical’ method of the number of species recorded per plot, with the goal of improving understanding of the potential drivers and maintenance of tropical diversity. Specifically, we addressed three main questions: (i) Which environmental predictors explain most of the variation in local tree species richness across the tropics? (ii) How do the environmental factors associated with tree species richness differ between broad versus local scales of sample plot spacing? (iii) Given the environmental predictors, does tree species richness at the 1-ha scale converge between tropical regions to give a consistent pantropical pattern?

A main goal of this study is to determine whether local tree richness and the environmental factors associated with it differ amongst the world’s major tropical regions. Such a global comparison is important to inquire whether community assemblage rules may be consistent across geographically disparate regions—in other words, evolutionarily conserved underlying environment–richness relationships across the tropical forest biome.

## RESULTS

**Figure 1.**
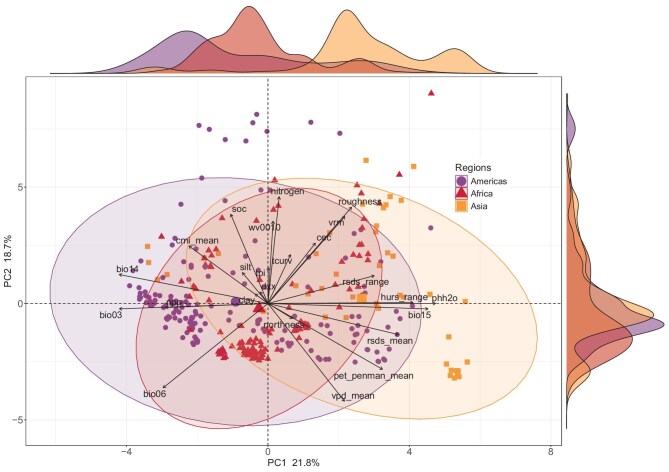
PCA of 24 environmental variables for 1-ha tropical tree plots in the Americas, Africa and Asia. Marginal density plots above and to the right of the biplot show the distribution of samples from each region along the first (PC1) and second (PC2) principal components, respectively. Percentages on each axis represent the variation explained by the respective principal component.

A principal component analysis (PCA) conducted on 24 environmental variables across 1-ha plots in the global tropics revealed regional differences, with the first two axes accounting for 40.5% of the variance in the variables (Table S1 and Fig. 1). Overall, the three regions exhibited moderate separation along the first principal component (PC1), especially between the Americas and Asia, but were largely overlapping along the second principal component (PC2). PC1 was primarily associated with moisture-related variables, including precipitation seasonality (bio15), the precipitation of the driest month (bio14) and the annual range of monthly relative humidity (hurs_range), along with soil pH (phh2o), isothermality (bio03), net primary productivity (NPP) and annual range and mean of monthly surface downwelling shortwave flux in air (rsds_range and rsds_mean) (Fig. 1 and Table S2). PC2 predominantly comprised soil-related variables, including total nitrogen, volumetric water content (wv0010) and soil organic carbon content in the fine earth fraction (SOC), as well as topography-related variables, including tangential curvature (tcurv), ruggedness and vector ruggedness measure (vrm) (Fig. 1 and Table S2). The Americas exhibited greater variation in environmental conditions along PC1, characterized by higher precipitation during the driest month and lower precipitation seasonality compared with Asia and Africa (Fig. 1). Specifically, Asia demonstrated the highest precipitation during the driest month and lower precipitation seasonality, while Africa showed the lowest variation in these environmental conditions. Variation along PC2 was similar among the three regions (Fig. 1).

**Figure 2.**
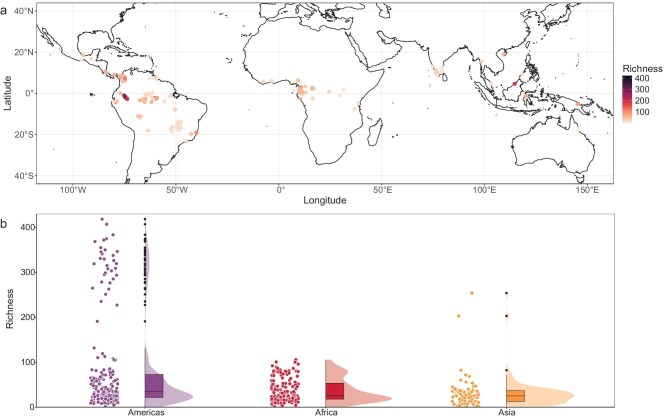
Tree species richness in 1-ha plots across the global tropics. (a) Distribution of 429 1-ha plots located in undisturbed old-growth forests used in this study. (b) Density distribution of the plots for the three regions. Box plots show the median and interquartile range of species richness estimated from rarefaction, alongside individual plot data for each region. Vertical lines extend to 1.5 times the difference between the quartiles and black points represent the outliers. Width of the distribution represents the number of plots at a given richness level. Similar plots for Fisher’s α and observed richness are shown in Figs S3 and S4. Review drawing number: GS京(2025) 2372号.

**Figure 3.**
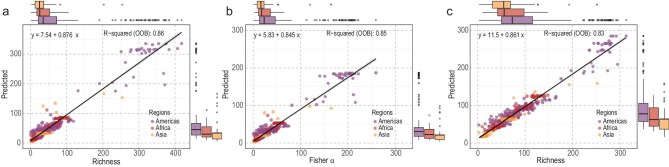
Predicted tree species richness according to non-spatial RF based on 24 environmental variables compared with richness estimated from (a) sample-based rarefaction, (b) Fisher’s α and (c) observed richness. Tree inventory data were obtained from 429 1-ha plots of old-growth tropical forest located across the Americas, Africa and Asia. The out-of-bag *R*^2^ reflects the performance of each RF model based on observations that were excluded from the training subset for each tree. Box plots above and to the right of each graph show the estimated and predicted richness between regions, respectively.

We obtained the species richness estimated from rarefaction based on sample coverage (Fig. 2) and Fisher’s α from a standardized sample of stems. By analysing non-spatial and spatial RF based on the observed richness, Fisher’s α and the richness from rarefaction data returned high ‘out-of-bag’ *R*^2^ values (0.83–0.88), demonstrating a robust ability to predict 1-ha-scale tree species richness from the 24 environmental predictors across the global tropics (Fig. 3 and Table S1).

**Figure 4.**
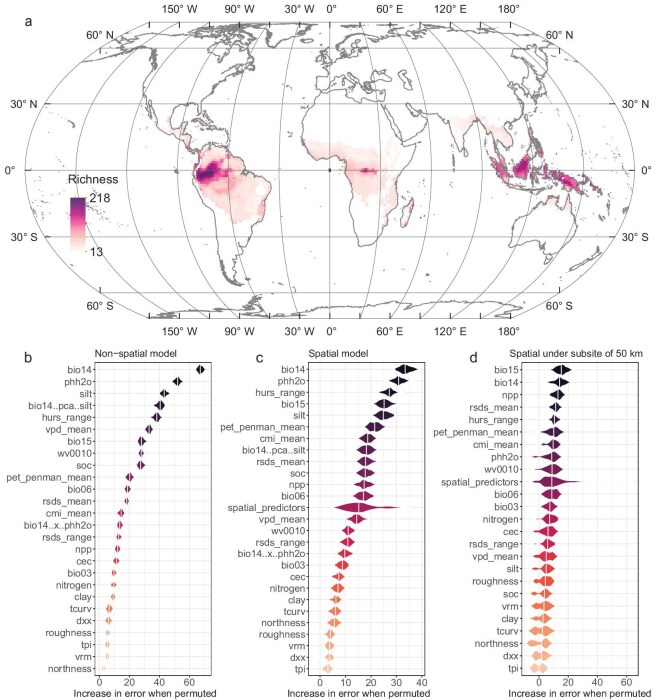
Predicted tree species richness at the 1-ha scale and importance of environmental variables based on non-spatial and spatial RF. (a) Local tree species richness estimated from rarefaction and predicted using non-spatial RF across the tropics. (b) Importance of environmental variables according to non-spatial RF, which does not account for spatial autocorrelation among forest plots. (c) Importance of environmental variables according to spatial RF, which accounts for spatial autocorrelation among forest plots. (d) Importance of environmental variables according to spatial RF with thinning of samples by 50 km. Review drawing number: GS京(2025) 2372号.

Training the RF for each of the three richness measures revealed that the predicted tree species richness at the 1-ha scale was highly heterogeneous across the tropics (Fig. 4a and Figs S6a and S7a), with the predicted richness highest in western South America, particularly the Andean–Amazon foothills and Colombian Chocó, the major islands of Southeast Asia, and New Guinea (Fig 4a). Low levels of predicted local species diversity were found across most of tropical Africa, eastern and southern Amazonia and continental Southeast Asia.

Analysis using non-spatial RF revealed that bio14 was identified as the most important single variable for predicting tree richness, followed by phh2o (Fig. 4b). The interaction between the precipitation of the driest month and the proportion of silt particles in the fine earth fraction (silt) also contributed significantly to the model (Fig. 4b). Spatial RF, which accounted for spatial autocorrelation (see Methods S1), showed a higher importance of moisture-related variables, including hurs_range, bio15, the mean monthly potential evapotranspiration (pet_penman_mean) and the mean monthly climate moisture index (cmi_mean) (Fig. 4c). In contrast, the importance of soil-related variables, such as pH and silt, remained relatively consistent, while the interaction between the precipitation of the driest month and silt (bio14..pca..silt) decreased in importance. An additional analysis in which non-spatial and spatial RF were applied to a thinned set of sample locations separated by at least 50, 100 and 200 km showed a diminished importance of soil-related variables such as pH, SOC and silt (Fig. 4d and Fig. S8b and d). This reduction was particularly pronounced with thinning of 100 and 200 km, for which the importance of soil pH and silt decreased markedly. In contrast, variables associated with moisture (e.g. hurs_range, cmi_mean), growing season (e.g. NPP), solar radiation (e.g. rsds_mean) and topography (e.g. topographic position index [tpi]) increased in importance.

According to spatial RF, the Americas, Africa and Asia exhibited similar relationships between tree species richness and six dominant environmental variables: bio14, phh2o, hurs_range, bio15, silt and pet_penman_mean (Fig. S9). In all three regions, the tree richness was highest in areas with abundant moisture in the driest month (bio14; Fig. S9a). Conversely, the richness was low in areas with high soil pH and silt fraction, except in Asia, where the richness increased with silt (Fig. S9b and e). High tree richness was also associated with a low annual range of monthly near-surface relative humidity, precipitation seasonality and mean monthly potential evapotranspiration, peaking when these were approximately 5%−6%, 25–35 mm and 110–115 mm/month, respectively (Fig. S9c, d and f). Notably, the Americas displayed a large variation in the precipitation of the driest month, soil pH and silt, whereas Asia exhibited substantial variation in the humidity range, precipitation seasonality and monthly potential evapotranspiration. Africa showed a low variation in these parameters compared with the other two regions.

The contribution of the six dominant environmental variables to local tree species richness varied within and across the regions. In areas with high precipitation, low seasonality in rainfall and low soil pH (Figs S10 and S11), the predictive contribution of these climatic variables to species richness within the RF model was reduced (Figs S12 and S13). For example, in areas with low soil pH (∼3.7–4), such as the Amazon (Fig. S10b), the importance of soil pH in the spatial RF decreased (Fig. S12b). By contrast, in areas characterized by high soil pH, such as India and southeast Africa, this soil parameter was a critical predictor in the model (Fig. S12b).

The negative binomial generalized linear models (glm.nb) demonstrated a weaker ability than the RF to predict local tree species richness, whether estimated from rarefaction (OOB *R*^2^ = 0.69), Fisher’s α (OOB *R*^2^ = 0.70) or observed richness (OOB *R*^2^ = 0.72) (Fig. S14). Moreover, in contrast to results from the spatial RF, the glm.nb found that Africa and Asia exhibited similar levels of local tree species richness, both of which were significantly lower than those of the Americas (Tables S6 and S7). Despite those differences, the direction and significance of the effects of important precipitation (e.g. bio14, bio15, mean monthly vapor pressure deficit [vpd_mean], hurs_range, temperature (mean monthly minimum air temperature of the coldest month [bio06]) and soil variables (e.g. pH, silt) were similar between the two approaches (Tables S6 and S7). Variables showing a significant interaction between Africa and the Americas included cmi_mean, hurs_range, vpd_mean, clay proportion, soil pH and tcurv (Tables S6 and S7). Both the glm.nb and RF models showed consistent results, indicating that variables such as bio14, pH, hurs_range, silt and bio15 are important factors in controlling α diversity. While RF excels in predictive power (*R*^2^ = 0.86), capturing complex, nonlinear relationships, the glm.nb model (*R*^2^ = 0.69) also explains a large portion of the richness (Fig. 2 and Fig. S14). This demonstrates glm.nb’s ability to capture substantial data variability despite its lower *R*^2^. Crucially, glm.nb shows significant regional differences between the three regions, confirming broad-scale patterns. While the results from glm.nb and RF diverged in important ways, the RF model is given precedence here due to its superior OOB *R*^2^ values and capacity to incorporate nonlinear effects [27,28]. However, the glm.nb model offers the advantage of interpretability and simplicity, which can be crucial for understanding the underlying factors driving richness. Therefore, while RF may provide superior predictive accuracy, glm.nb remains a valuable tool for future additional exploratory analysis and hypothesis testing.

**Figure 5.**
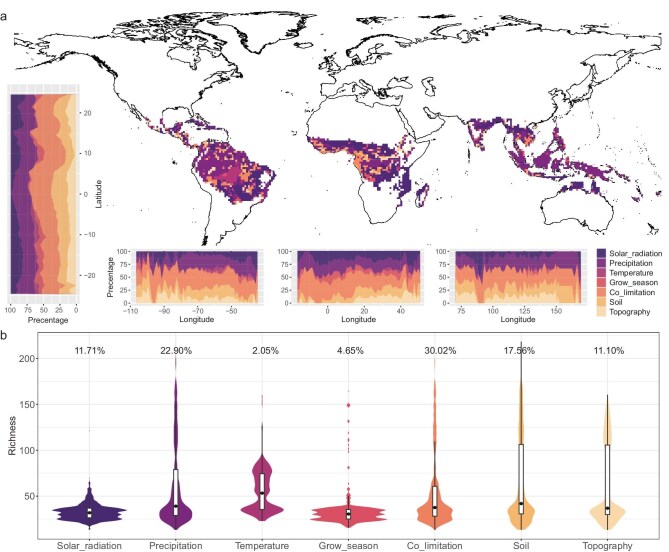
Geographical distribution of predominance of seven categories of environmental variables. Co-limitation refers to areas in which no single factor dominates. (a) Driving factors distribution pattern in pantropical regions, along longitude and latitude. (b) Percentage of main categories driving factors in tropical region. Detailed variables for different drivers are explained in Table S3. Review drawing number: GS京(2025) 2372号.

When environmental variables were grouped into seven categories—temperature, precipitation, growing season, solar radiation, soil, topography and co-limitation, which means no single type of environmental factor dominates—the local tree species richness was largely explained by the same three categories: co-limitation, precipitation and soil (Fig. 5). Referring to areas in which no single category dominates, co-limitation represented the most important category, accounting for 30.02% of the variation in local tree species richness (Fig. 5b). Precipitation (22.90%) and soil (17.56%) were the next most important categories, followed by solar radiation (11.71%) and topography (11.10%). In contrast, temperature (2.05%) and growing season (4.65%) were relatively unimportant overall, but increased in significance at higher latitudes and altitudes within the tropics (Fig. 5a). Solar radiation and growing season tended to gain importance relative to other categories where the species richness was low, particularly near 10°N and 10–20 °S latitude. Along the latitudinal gradient, co-limitation, moisture and soil were the dominant categories explaining the richness variation in the three regions. Compared with other regions, along the longitudinal gradient, the growing season and solar radiation were more important in Africa, while the soil and precipitation categories were most important in Asia. Overall, species richness in most tropical regions is constrained by multiple environmental factors, indicating a co-limitation effect, particularly in areas of higher species richness close to the equator.

## DISCUSSION

### The relationship between environmental factors and species richness

The RF analysis and glm.nb model showed that variation in tropical tree species richness was consistently predictable by using 24 primary environmental variables across the three main biogeographic regions of the tropics. Local tree species richness predicted by RF were strongly correlated with those based on sample coverage estimation from rarefaction and Fisher’s α across the tropics, with the observed raw richness and predicted richness showing similar results in the RF analysis (Table S5) and relatively lower in the glm.nb model. While these relationships are empirical, the strength of the predictions implies that, across the world’s tropical forests, local species richness at the 1-ha scale is largely deterministic. The results showed that local tree diversity tends to converge on similar levels of richness when conditions are similar. This trend holds despite the fact that a minority of genera and almost no species of trees exist in common between the studied biogeographic regions, with different families and genera having undergone their own patterns of diversification within each region [29–32].

A noteworthy nuance emerges when comparing the relationship of individual environmental variables to species richness: although the correlations between any single environmental variable and richness are relatively weak, simultaneous incorporation of all environmental predictors in the RF analysis achieved high predictive power (*R*^2^ > 0.86). Furthermore, sensitivity analysis highlighted that the importance of these interactions is context-dependent: the trained RF model reflected the specific range of environmental conditions in the current dataset. While predictive relationships might shift with the inclusion of additional forest plots (taking in a greater range of local environments or subregions), the sample size as it already stands is very large and in diverse settings, so we regard these inferences to be robust and unlikely to change substantially with the addition of more samples.

Our results suggest that moisture-related variables are more important at the large scale, while the soil-related variables play a relatively more important role at the local scale. The relatively strong local effect of soil factors is consistent with the mosaic of soil-influenced habitats that can exist at a fairly local scale within the tropics, producing differences in tropical tree communities [33,34].

The strongest climate-related predictors of tree richness identified in the RF and sensitivity analysis (Figs 4 and 5) were those reflecting aspects of the moisture supply to vegetation, with variations in temperature playing a less important role. The estimated npp itself is high on the list of predictors at large scales (50, 100 and 200 km; Fig. 4d and Fig. S8). In general, the climate factors that emerge as importantly correlated with tree species richness were all consistent with conditions that favor plant growth and productivity rather than conditions that impose severe water stress or suppress photosynthesis due to low temperatures. This reinforces and refines the patterns linking tree diversity and plant physiology/productivity that have long been noted [1,2,6,35]. While relationships between species richness and climate have been demonstrated in other studies [36,37], what is striking in these new results is how predictable the overall relationship is when soil factors were included (discussed below) and how consistent the relationship is across different regions of the tropics.

The question of why such environment–richness relationships exist for trees is one of the greatest conundrums in ecology and evolution [2]. Explanations vary from the legacy of a moist tropical origin of angiosperms [38] to the niche partitioning capabilities of more productive ecosystems [35] to the lack of physiological extremes, which allows functional equivalency and thus both greater diversity of growth architectures [39–41] and extensive niche overlap [3] that favors the coexistence of high species richness.

Certain internal patterns of influence on species richness within each tropical region are discernible (Fig. 5 and Figs S10–S13). Towards higher latitudes of the tropics, species richness was more strongly impacted by factors related to temperature and growing season, whereas, closer to the equator, moisture-related factors were more important (Fig. 5). Although particular categories of environmental factors cause a variation in the species richness in some areas, most regions are subject to co-limitation by multiple categories of environmental factors (Fig. 5). The high species richness observed in tropical regions may be maintained through the interaction of multiple factors and co-limitation [19]. In natural environments, plant growth is impacted by multiple variables [42], with the optimal growth occurring when these interacting variables attain a state of conducive equilibrium [43]. In this study, these co-limitation areas likely create environmental conditions under which resources are relatively balanced, favoring the survival of various species and thus making these areas high in species richness. Given the relatively limited attention given to the role of soil parameters in affecting the patterns of tropical tree diversity, soil pH surprisingly emerges as influential in the RF analysis, especially at the local scale (Fig. 4b and c, and Fig. S8). The role of soil pH has long been known as an influence on more local-scale patterns in tropical forest composition [34] and soil pH is known to play a major role in plant ecology in general [44]. Soil pH correlates with a range of other factors, such as nutrient concentrations and availability, including the mobility of toxic ions such as aluminum [45]. The species composition of vegetation can itself influence the soil pH and other soil factors [46–48], but the possibility of complex feedback loops between species diversity and soil being significant on the pantropical scale can only be considered as speculative.

While a lower soil pH may be seen as a physiologically more extreme environment, for the above reasons, in fact, tree species richness tends to be highest in the lowest pH soils in our dataset (Figs S9b and S10b). Empirically, this supports previous results from studies in other biomes that plant species richness peaks in low soil pH of ∼4 [49] and declines as the pH increases (Fig. S9b). A widely discussed principle in plant ecology is that a certain degree of physiological ‘stress’—such as low soil pH—may suppress plant growth and productivity, reducing the competitive ability of faster-growing generalists [44]. In the context of disturbance events at varying scales, this may reduce competitive exclusion and enable a greater number of species to coexist [44,50,51].

While it is reasonable to focus on the potential effect of environmental factors on the trees themselves, it is also crucial to consider that other factors could be at work, without directly involving the physiology of the trees as the primary driver. For instance, the Janzen–Connell hypothesis suggests that the diversity levels in tropical forests are controlled by the intensity of attacks by insect herbivores and pathogens [2,52–54]—with constantly warm and moist conditions favoring the specialization of insect pest or pathogen populations and the strong density-dependent control of tree populations allowing more tree species to coexist locally. There is considerable evidence that a degree of selective pest pressure can maintain diversity in plant communities, but inconsistent evidence that density-dependent mortality is stronger in the tropics than in temperate regions [2]. However, to explain the patterns seen here, the pest-pressure effect would need to operate in a finely modulated way along environmental and species richness gradients within the tropics, quite aside from whether it differs between tropical and temperate regions. A potential effect of the soil pH on herbivory—perhaps with respect to the extent of the species pools on different soil types and perhaps in affecting the nutrient or secondary compound content of plants or the growth rate of fresh edible tissues—might also be involved in the observed relationship with soil factors.

### Convergence in richness amongst biogeographic regions

From the analysis of this dataset, there is no obvious evidence for any regional influence producing anomalously high or low tree species richness relative to the overall pantropical trend. When 1-ha richness data from all the regions are overlaid on the same scatterplot, the Americas, Asia (including Wallacea and Australasia) and tropical Africa all fall close to one another, within margins of error (Fig. 3 and Figs S14‒S16). The spatial RF model demonstrates that analogous environments support comparable richness levels, irrespective of regional species pools (Fig. S17). This contrasts with the overall comparison of regions without accounting for equivalent environments. Most strikingly, there is particularly high species richness for some plots in the Americas (Fig. 3 and Figs S14‒S16). One of the primary predictors, and possible drivers, of the elevated diversity in the neotropics appears to be this region’s consistently higher levels of moisture, as expressed by a range of factors in the RF model [55]. This may be further reinforced by the region’s relatively low soil pH values, which we find correlate significantly with species richness independently of climate. When the RF model was implemented, the anomalously high richness of the Americas disappeared and was explicable almost exclusively in terms of empirical environmental factors.

The close similarity in richness between tropical regions, when compared in terms of the combinations of environmental factors that best predict richness, is evident despite the divergent geological, climatic and evolutionary histories of these regions that span tens of millions of years [29,38,56,57]. Independent evolution and diversification of clades has given a distinctive taxonomic composition to the forests in each of the main regions—the Americas, South East Asia (and Wallacea/Oceania) and tropical Africa [38,56,57]. In each region, the composition and distribution of flora have been influenced by continental collisions, mountain building and the cooling and drying of the climate in the Cenozoic [30,56–59]. For instance, tropical Africa lost a large proportion of its previous diversity during the late Cenozoic/Quaternary due to the drying of the climate, especially during glacial episodes [38,56,57], and now has restricted total richness and phylogenetic diversity of trees at the regional scale [23,30]. The fossil pollen record also shows that the rainforests of northern Australia have been through strong drying episodes in which their areal extent was severely restricted [38,56,57]. While the climate history of the tropical forests of India is not well known, it is possible that, as a relatively dry and small rainforest enclaves it, it could also have gone through past climate bottlenecks [56]. The uplift of the Andes created a complex topographical gradient that facilitated allopatric speciation and the establishment of numerous microhabitats, leading to species diversification [60,61].

Despite all of these different histories and potential trajectories, brought about by climatic and tectonic history, and regional-scale diversifications, it is striking that, from the perspective of the 1-ha-sample scale, all the regions that we distinguish here adhere closely to the same pantropical pattern of richness in relation to present-day environmental conditions (Fig. 2 and Figs S14‒S16).

The very close correspondence in tree species richness between the regions, despite all of the historical legacy factors that could have potentially caused a divergence in diversity, implies the existence of precise control by ‘governance’ factors in the forest community that tends to cause the richness to settle at a particular level. Many potential mechanisms have been put forward to explain how the tree species richness in tropical forests is maintained [2], including those discussed above. It is, however, surprising that the mechanisms at work are able to operate so precisely, all across the tropics, to modulate the local-scale richness when there are so many factors that would be expected to cause the richness to diverge. Overall, the mechanisms invoked to explain the high tree species richness of tropical forests and its variation within the world’s tropical forests can be grouped into two kinds. Disequilibrium hypotheses invoke time-dependent processes of the progressive buildup of diversity by diversification or migration and its destruction by extinction [58]. According to such mechanisms, there is no ‘lid’ on the maximum diversity in the tropics and differences in diversity reflect the balance between diversification events and extinction events. Equilibrium hypotheses, by contrast, assume that there is a set capacity to the number of tree species that can coexist in any one place and that differences in diversity reflect differences in this capacity. This set of mechanisms necessarily depends upon differences in niche structuring—e.g. the number of discrete niches available due to the heterogeneity of microenvironments [62], the narrowness of specialized niches that is possible in a given environment (affecting the opportunities for slotting in extra species) [63] or the degree of overlap in tree species niches that can occur before competitive exclusion begins to reduce the diversity [64,65]. In our opinion, the results of this study support a predominance of equilibrium- or niche-based mechanisms, as the relationships between environmental parameters and hectare-scale species richness are so strongly convergent between different parts of the world. If the vagaries of diversification and extinction were more important in affecting the richness, then we might expect to see large differences in local diversity between different regions under similar environmental conditions.

Whatever the ecological mechanisms that mediate the relationship between environmental parameters and tree species richness, they appear to operate consistently and in combination along a sliding scale in terms of the levels of richness that they permit. Whilst it is intuitively hard to accept that such mechanisms could exert their effects so precisely between different regions of the world with their own distinct tropical tree floras that have been separated for tens of millions of years, this is indeed what the results of our study suggest.

## CONCLUSIONS

Although regional species pools differ due to distinct geological histories, analogous environmental conditions yield similar local richness patterns. The broad-scale approach employed in this study, combined with spatial RF analysis, has demonstrated that there is a striking predictability in pantropical tree species richness sampled at the hectare scale. This predictability involves a combination of environmental factors, with climate and to some extent soil showing strong correlations with tree species richness patterns across and between tropical regions. Pantropical regions exhibit high species richness, primarily due to the intricate interplay of co-limitation that creates stable and balanced conditions. Although regional species pools differ, analogous environmental conditions yield similar local richness patterns. A multifactorial interplay of evolutionary and ecological mechanisms is presumably at work in controlling this consistency of species richness, preventing regional divergence. The observed variation in tropical species richness must be seen as the outcome of a range of different factors acting simultaneously, sometimes in parallel and sometimes in opposition to one another. It is possible that these multiple factors each act through a range of different ecological mechanisms, requiring separate elucidation. These findings decisively strengthen the case for the niche theory, revealing its remarkable predictive power. They provide compelling evidence that niche segregation is a pervasive and dominant force, structuring communities from local patches to broad regional landscapes, even in the presence of diverse regional species assemblages. Furthermore, by elucidating the contemporary environmental controls on species richness, this work contributes a vital context for predicting plant diversity changes under future warming scenarios.

Whilst identification of the true underlying mechanisms involved remains a fundamental challenge for ecology, this study contributes to the ongoing challenge in ecology to identify and understand the controls on biological diversity.

In this paper, we had originally hypothesized that local-scale tree α diversity across the global tropics can be consistently predicted by using contemporary environmental variables, such that analogous environmental conditions will support similar levels of richness, irrespective of deep historical biogeographic divergences. This hypothesis has survived its test, with striking predictability of species richness as sampled at the hectare scale. We also hypothesized that variation in richness on more localized scales between samples would be governed by a distinct set of influences. Despite some subtle scale-related differences, this was essentially disproven, with similar sets of environmental factors governing throughout.

It is necessary to keep in mind, however, that our study is confined to data obtained at the 1-ha sampling scale and that other patterns may emerge at other local sampling scales [10] or for other life forms (e.g. lianas)—either in sub-hectare or larger plots, or at the level of beta-diversity turnover rates. This awaits other studies and the additional information that comes from these will shed more light on the mechanisms at work behind tropical tree diversity patterns. The findings of this study are derived from current sample surveys and the uneven distribution of these samples may have affected our results. We advocate for an increase in sample surveys in tropical regions and the development of a more comprehensive tropical sample database in future scientific research.

Intriguingly, other aspects of the community structuring of tropical forests may be found to show striking convergence patterns across the tropics. Cooper *et al.* [32] have recently revealed a strong convergence in relative abundance data—rather than richness, as is shown here—in forest plots throughout the tropics. There is a need for further careful comparisons of the structure and functioning of tropical forests across different regions in order to understand how closely they have maintained their similarities and to better understand the driving mechanisms behind the observed patterns.

## MATERIALS AND METHODS

### Tree data and richness

Tree inventory data were assembled from the PFN of old-growth (not recently logged or cleared) closed canopy forest plots from across the global tropics, including the tropical dry forest and its various transitional forms to the tropical rainforest [23]. All trees, defined as free-standing woody individuals (including palms) with a diameter at breast height (1.3 m) of ≥10 cm, were measured and identified in each plot (Data S1). If the species names could not be determined, then plot-specific morphospecies were recorded, with the closest taxonomic assignment. All morphotypes included here were identified to the Linnean species, genus or at least family level. Unknowns at the Linnean family level were not included.

The numbers of individuals and species were obtained from a total of 429 1-ha plots located in the Americas (197 plots), Africa (150 plots) and Asia (82 plots) (Fig. 2, Figs S3 and S4, Data S2). The Asian region included Wallacea and Australasia based on a shared floristic affinity [23]. The geographical boundaries of the tropics were defined based on the map in [66].

In addition to the observed species richness, we analysed the richness in each 1-ha plot estimated by using sample-based rarefaction and Fisher’s α (see Methods S1). Both of these methods are widely recognized and frequently used measures of species richness [24]. Sample-based rarefaction is often considered a more reliable estimator of true species richness in a community, particularly when sampling is incomplete, as is frequently the case in diverse assemblages [25,67]. This technique allows a more accurate comparison across communities by taking into account undetected species and different levels of sampling effort. We used the function ‘estimated’ from the iNEXT package in R [68] to estimate species richness based on sample coverage, which accounts for the completeness of sampling by estimating how well a community has been sampled. The richness was standardized to the same sample coverage (1 ha) to compare the survey completeness of each sample size, providing a robust measure that accounts for uneven sampling efforts across the regions. The rarefaction curves were plotted to visualize the richness of species across the coverage sampling efforts, indicating that the sample coverage values for all the localities are quite high—most of them are >0.8 (Fig. S1).

Our primary analysis focused on species richness estimated from sample-based rarefaction to ensure the robustness of our estimates. Fisher’s α was also examined given its applicability to communities in which the species follow a log-series pattern with high proportions of rare species and for detecting the influence of abundance distributions on species diversity [26,69]. Compared with other diversity measures, these two indices showed the strongest correlation with the observed richness (Table S4 and Fig. S2; see also Methods S1).

### Environmental data

We collected data surfaces for an initial set of 65 environmental predictors, including bioclimatic, soil and topographic variables (Data S3‒S5). Bioclimatic data were sourced from CHELSA (Climatologies at high resolution for the Earth’s Land Surface Areas; http://chelsa-climate.org/), which provides climate data at a spatial resolution of 30 arc-seconds (∼1 km²). Soil data were obtained from the ISRIC World Soil Information SoilGrids dataset (https://data.isric.org/), which provides model-interpolated predictions of soil parameters at a resolution of 250 m. These predictions are derived from the integration of data from thousands of soil cores from across the globe, with geological, surface sediment, topographic, normalized difference vegetation index and climatic background information [15]. Topographic data with a spatial resolution of ∼1 km were downloaded from EarthEnv (http://www.earthenv.org/topography). Important environmental variables were selected by using the Boruta algorithm from the ‘Boruta’ package [70] in R (Data S6). Multicollinearity was addressed by assessing the correlation and variance inflation factors, resulting in a refined set of 24 environmental predictors for the RF analysis (Table S1).

### Spatial RF

To predict species richness based on multiple environmental variables, we applied a RF combined with spatial regression by using the ‘spatialRF’ package in R [22], which can be applied on regular or irregular data [71]. This package enhances traditional RF techniques by accounting for spatial autocorrelation when observations are not independent, but rather show geographic relationships. Spatial autocorrelation based on Moran’s I index was explicitly taken into account to improve the model accuracy in capturing spatial patterns. To ensure robust evaluation of the model, we employed cross-validation by dividing the data into 30 spatial folds for training and testing. Additionally, spatially thinned occurrence data were generated by using the ‘thin’ function from the ‘spThin’ package [72], which filters occurrence locations to ensure they are a set minimum distance apart (e.g. 50 km). This spatial thinning reduces bias from uneven species collections and was used in RF analysis to compare the results from the full dataset (see Methods S2).

### Statistical analysis

We used linear regression to examine the relationship between the species richness predicted by using non-spatial and spatial RF and that estimated from the sample-based rarefaction, Fisher’s α and observed richness. The PCA was performed to explore variation between the 24 environmental variables in the three major tropical regions: the Americas, Africa and Asia. We used the ‘FactoMineR’ package [73] in R to implement the PCA and the ‘factoextra’ package [74] to generate a biplot that enabled visualization of the results.

Spatial RF excels at capturing complex, nonlinear relationships and identifying important predictors, primarily due to its predictive power [75]. As a complementary approach, the Generalized Linear Model with Negative Binomial distribution (glm.nb) was used. This allowed us to validate the RF results by statistically testing broad-scale factors such as continental differences and examining linear relationships, combining data-driven prediction with statistical inference. To quantify the effects of the 24 environmental variables on the species richness across the three tropical regions, we applied a negative binomial generalized linear model (glm.nb) by using the ‘MASS’ package in R and compared the results with those from spatial RF. Spatial RF excels in capturing complex, nonlinear relationships between species richness and environmental variables, but does not provide interpretable coefficients that explain the direction and magnitude of those relationships [76]. As a complementary approach, glm.nb was used to validate the robustness of the RF results by enabling the identification and interpretation of regional differences and linear relationships between environmental predictors and species richness. Both the main effects and the interactions between environmental variables and regions were examined by using this approach (see Methods S3).

### Sensitivity analysis

To determine which class of environmental variables (e.g. precipitation, temperature, soil, etc.) best explained the variation in the local tree species richness, we classified the 24 environmental variables into seven categories—temperature, precipitation, growing season, solar radiation, soil, topography and co-limitation—and conducted a sensitivity analysis of the RF model by following the methods of Saltelli *et al.* [77] and Liang *et al.* [19]. All of the above analyses were performed in R version 4.2.3 (R Core Team, 2024). The R script used for all analyses is provided in Data S6.

We conducted a sensitivity analysis through the following steps:

Step 1: Using all environmental variables *X*(*s*), we applied the RF model to simulate the predicted species richness *Y*_all_(*s*):
  \begin{eqnarray*}
{Y}_{all}( s ) = f\left( {X\left( s \right)} \right)\!,
\end{eqnarray*}
  where *f*() represents the RF model, *X*(*s*) represents the values of the environmental variables and *s* represents the six categories to which the environmental variables belong: E1, temperature; E2, precipitation; E3, growing season; E4, solar radiation; E5, soil; E6, topography (Table S3).
Step 2: We then applied the RF model to predict the tree species richness based on all the environmental variables except those belonging to E1, *S*_−E1_ (*s*):
  \begin{eqnarray*}
{Y}_{ - E1}( s ) = {f}_{ - E1}( {( {X - E1} )( s)} ),
\end{eqnarray*}
  where *f*_−E1_() represents the RF model simulated with all the variables except those associated with temperature and (*X* – E1) (*s*) represents the variables of the remaining five categories (E2–E6).
Step 3: We calculated the relative sensitivity of the predicted species richness to E1 from:
  \begin{eqnarray*}
R( {E1} ) = \left| {{Y}_{all}( s ) - {Y}_{ - E1}( s)} \right|\ /\ {Y}_{all}( s).
\end{eqnarray*}
Step 4: We repeated Steps 2 and 3 to calculate the relative sensitivity of each of the remaining categories E2–E6. For a given area, the category with the highest relative sensitivity and meeting the threshold of relative sensitivity of ≥1/7 was considered that which best explained the variation in the tree richness for that area.
Step 5: In areas in which the relative sensitivities were <1/7 for all the categories, we hypothesized that the tree richness was not related to any single category, but rather multiple categories of environmental variables. Therefore, we created a seventh category (E7) called co-limitation to characterize areas in which no single type of environmental factor dominates.
Step 6: Steps 1–5 were repeated to calculate the relative sensitivity of each of the seven categories, including E7. To visualize the regional variation in category importance, we calculated the relative sensitivity of each category as a percentage of that of all the categories and plotted this along latitudinal and longitudinal gradients spanning the tropics on a map.

## Supplementary Material

nwaf465_Supplemental_Files

## Data Availability

All datasets, R scripts and model output results have been uploaded to Figshare (Data S1–S7 and Appendix in Supplementary materials).
