# Supplementary material for: Analogous environments across the tropics have similar levels of tree species alpha diversity
Source: Natl Sci Rev. 2025 Oct 29;13(2):nwaf465. doi: 10.1093/nsr/nwaf465 (PMC12860206; doi:10.1093/nsr/nwaf465)
Supplement: nwaf465_Supplemental_Files [file nwaf465_supplemental_files.zip › Supplementary_files/Supplementary.docx]

**Supplementary Materials**

Analogous Environments Across the Tropics Have Similar Levels of Tree Species Αlpha Diversity

**Supplementary Methods**

**S1 Rarefaction and Fisher's alpha diversity**

We analyzed tree inventory data from 429 1–hectare (ha) old-growth tropical forest plots located across the Americas, Africa and Asia (Data S1−Data S2). Diversity measures such as species richness are sensitive to sampling effort, which refers to the intensity of effort spent collecting individuals or surveying areas. Therefore, it is appropriate to standardize sampling effort to enable the comparison of species richness across different regions and scales. We calculated diversity indices for each 1-ha plot, focusing on richness estimated from rarefaction based on sample coverage (Fig. S1), and Fisher’s α based on a standardized sample of stems (Data S4). Compared to other diversity measures (e.g., Shannon index and Simpson index), these two richness estimators showed the highest correlation with observed species richness (Table S1, Fig. S2). Moreover, the other diversity measures address evenness, whereas we were primarily interested in the number of species. For these reasons, and given their extensive use as robust measures of species richness [1,2], we used sample-based rarefaction and Fisher’s α for comparing local tree species richness across tropical regions and in relation to environmental predictors (see Supplementary Methods S2).

We apply rarefaction to standardize species richness, addressing the inherent dependence of observed richness on stem density across our plots. While 1-hectare plots are conceptually considered completely censused, actual stem densities can vary significantly between different forest types and regions. Rarefaction standardizes species richness by simulating the number of species that would be observed if each plot or sample had the same number of individuals [3]. This helps account for the fact that larger samples, or those with more individuals, are likely to contain more species. We used the function “estimated” from the *iNEXT* package in R [4] to estimate species richness based on sample coverage, which accounts for the completeness of sampling by estimating how well a community has been sampled. Richness was standardized to the same sample coverage (survey completeness), providing a robust measure that accounts for uneven sampling efforts across regions. This method is distinct from traditional “sample-based rarefaction” (Gotelli & Colwell, 2001), which typically involves accumulating discrete sub-samples or plots. Instead, our approach uses abundance data (number of stems per species per plot) to standardize diversity estimates to a common level of sample completeness (coverage). Sample coverage estimates the proportion of individuals in a community that belong to the species detected in a sample. It primarily relies on the number of singletons (species with one individual) and doubletons (species with two individuals); a higher proportion of singletons indicates less complete sampling. By standardizing to a common sample coverage, we ensure equitable and meaningful comparisons of biodiversity across plots with varying stem densities and underlying species rarity. The rarefaction curves were plotted to visualize the richness of species across coverage sampling efforts, the species richness standardized to sample coverage near 0.8 (Fig. S1).

Fisher’s α is a widely used diversity index that is particularly robust for measuring richness in tropical forests [5–8]. It assumes a log-series distribution of species abundance and is less sensitive to sample size than other diversity measures. Derived from the relationship between the number of species (*S*) and number of individuals (*N*), this index provides a measure of species richness that is relatively unaffected by differences in the number of individuals sampled. This makes it suitable for communities where species abundances follow a log-series distribution, as is common in tropical forests. We calculated Fisher’s alpha for each forest plot using the “fisher.alpha” function from the *vegan* package in R [9].

**S2 Spatial random forest**

Spatial random forest (RF) was run in R using the *spatialRF* package [10,11]. Starting with a set of 65 environmental variables (Data S3), we began this analysis by assessing the importance of each variable using the Boruta algorithm, which was based on RF model. It iteratively evaluates the importance of each variable and ultimately selected the variables that is most closely related to the target variable. The result suggested that 59 confirmed important variables and six unimportant variables (Data S6). Next, we used the “plot_training_df_moran” tool from *spatialRF* to evaluate spatial autocorrelation in the response variable (i.e., tree species richness) and the 59 important variables identified with the Boruta algorithm at different distance thresholds (12, 13, Fig. S5). The “auto_cor” and “auto_vif” functions were executed to reduce multicollinearity among the predictors using cor.threshold=0.9 and vif.threshold=10, which ultimately resulted in the selection of 24 environmental variables for subsequent analyses (Table S1).

Variable interactions were explored using the “the_feature_engineer” function. Interactions generated via multiplication were labeled "a..x..b" while interactions generated via PCA were labeled "a..pca..b". A non-spatial RF model was then constructed using the “rf” function. The “get_importance_local” function was used to calculate local importance by evaluating the increase in error explained by each of the 24 environmental predictors based on permutation over all decision trees. This allowed us to map spatial changes in the importance of each predictor.

To assess the overall performance of non-spatial and spatial RF models, the “print_performance” function was used, which yielded metrics like out-of-bag (OOB) *R*^2^, overall *R*^2^, and pseudo-*R*^2^. We used the “rf_evaluate” function to apply spatial cross-validation by splitting the data into 30 spatially independent folds, setting 75% as training data and 25% as testing data. Model performance was tuned using the “rf_tuning” function, in which hyperparameters such as num.trees, mytry, and min.node.size were adjusted for optimal performance. Given that RF is a stochastic algorithm, we used the “rf_repeat” function to repeat model execution 10 times, which produced a distribution of importance scores for each predictor. In the spatial model, hyperparameter tuning was carried out using spatial cross-validation to ensure the model performed well on the data that were not included in the training process.

Model residuals were tested for spatial autocorrelation using Moran's I with the “plot_moran” function. When significant spatial autocorrelation was detected (p-value > 0.5), the model was transformed into a spatial model using the “rf_spatial” function. After applying the spatial model, we found that no significant spatial autocorrelation remained in the residuals at any distance. Comparing variable importance plots between non-spatial and spatial models enabled us to identify additional spatial predictors, which represented neighborhood effects across different spatial scales. The “select_spatial_predictors_sequential” function was used to find a smaller subset of spatial predictors that maximized *R*^2^ and minimized Moran's I in the residuals. Spatial thinning was employed to assess whether the importance of variables changes across different scales. We used distances of 50, 100 and 200 km to thin the sample sites.

**S3 Negative binomial generalized linear model**

To quantify the effects of specific environmental predictors and their interaction with region on tree species richness, we employed a negative binomial generalized linear model (glm.nb) to compare results with those obtained from the spatial RF model. While spatial RF is effective at capturing complex, non-linear relationships between species richness and environmental variables, it does not produce interpretable coefficients that explain the direction and magnitude of these relationships. In contrast, glm.nb represents a complementary statistical approach by providing interpretable coefficients that can be compared with the RF results. This is particularly useful for identifying and interpreting regional differences and linear relationships between environmental predictors and species richness. However, glm.nb cannot substitute for the power of nonlinear multivariate analysis provided by RF.

We used the “glm.nb” function from the *MASS* package [14] and the *lme4* package [15] in R to construct generalized linear models (GLMs) that appropriately accounted for the nature of the data. Species richness was the response variable while the 24 environmental variables were used as predictors. “Region” was included as an interaction term. We initially applied a Poisson GLM, which is appropriate for count data assuming that the mean and variance of the data are equal. However, model output showed a residual deviance of 5794.1 with 354 degrees of freedom, indicating significant overdispersion. We then applied a quasi-Poisson GLM, which allows the variance to be a linear function of the mean and thereby accommodates extra variability in the data. Despite this adjustment, the estimated dispersion parameter was 19.85, which is much higher than the recommended range (1–15) for a quasi-Poisson model. Given the overdispersion observed in the Poisson and quasi-Poisson models, we turned to a negative binomial GLM (glm.nb). This model provided a dispersion parameter closer to 1, suggesting a better fit of the data by appropriately addressing the overdispersion issue present in the Poisson-based models.

**Supplementary Figures**

**
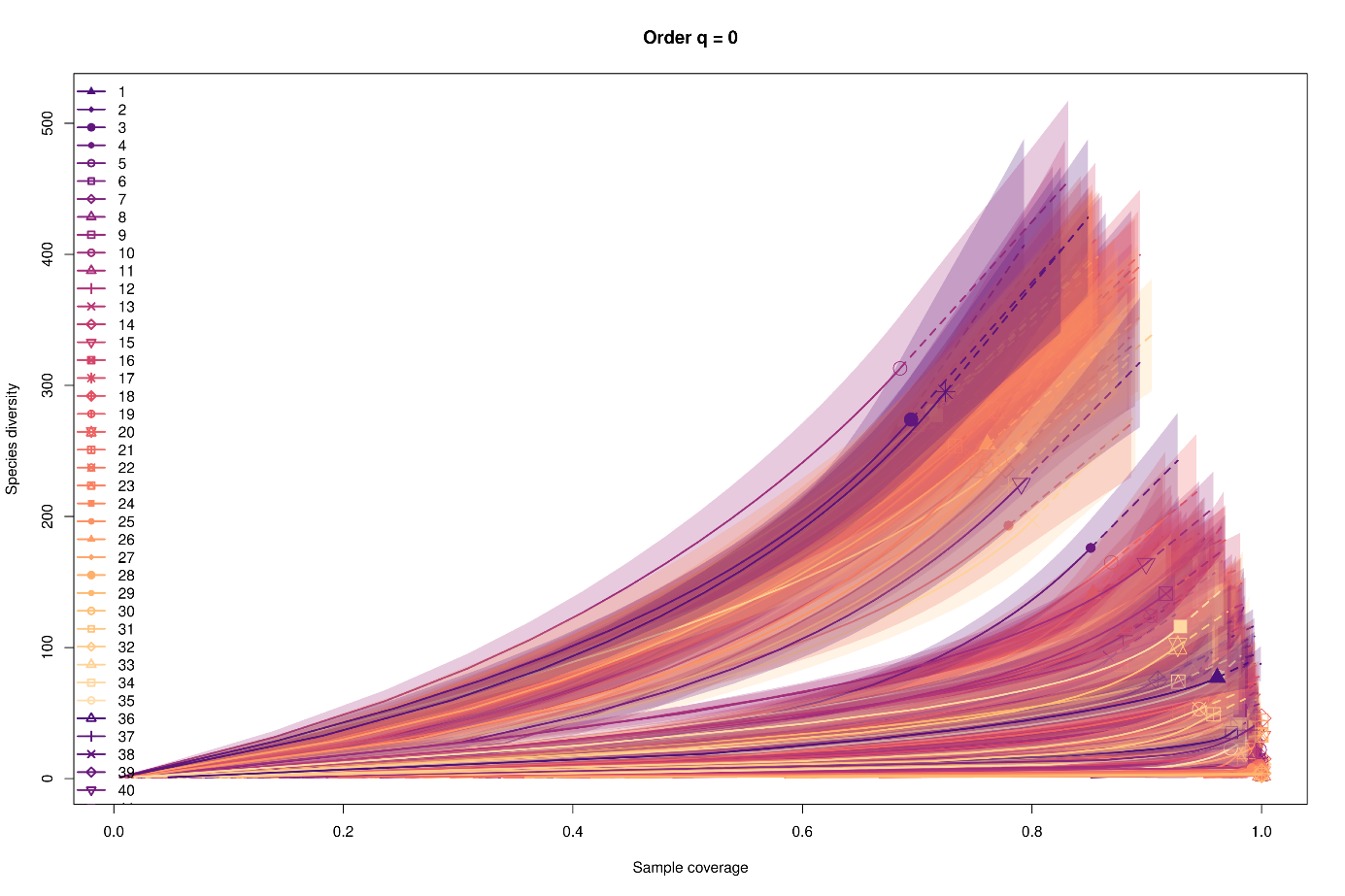
**

**Fig. S1.** **Rarefaction curves based on sample coverage estimation for 429 1-ha old-growth forest plots located across the global tropics.**

**
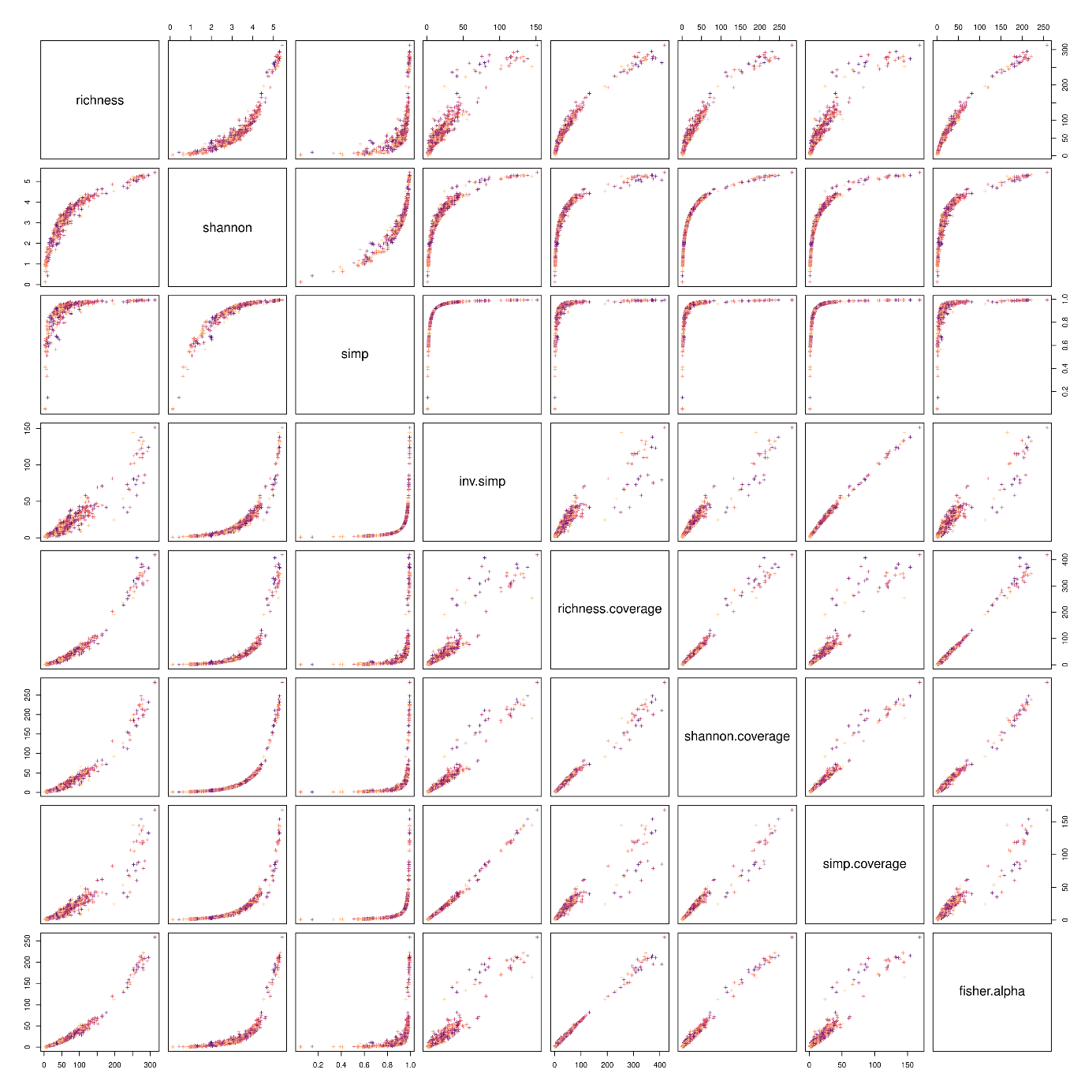
**

**Fig. S2.** **Pairwise comparisons of** **observed raw richness (richness), Shannon index (shannon), Simpson index (simp), inverse Simpson index (inv.simp), richness estimated from sample-based rarefaction (richness.coverage), Shannon index estimated from sample-based rarefaction (shannon.coverage), Simpson index estimated from sample-based rarefaction (simp.coverage), and Fisher’s alpha (fisher.alpha).**


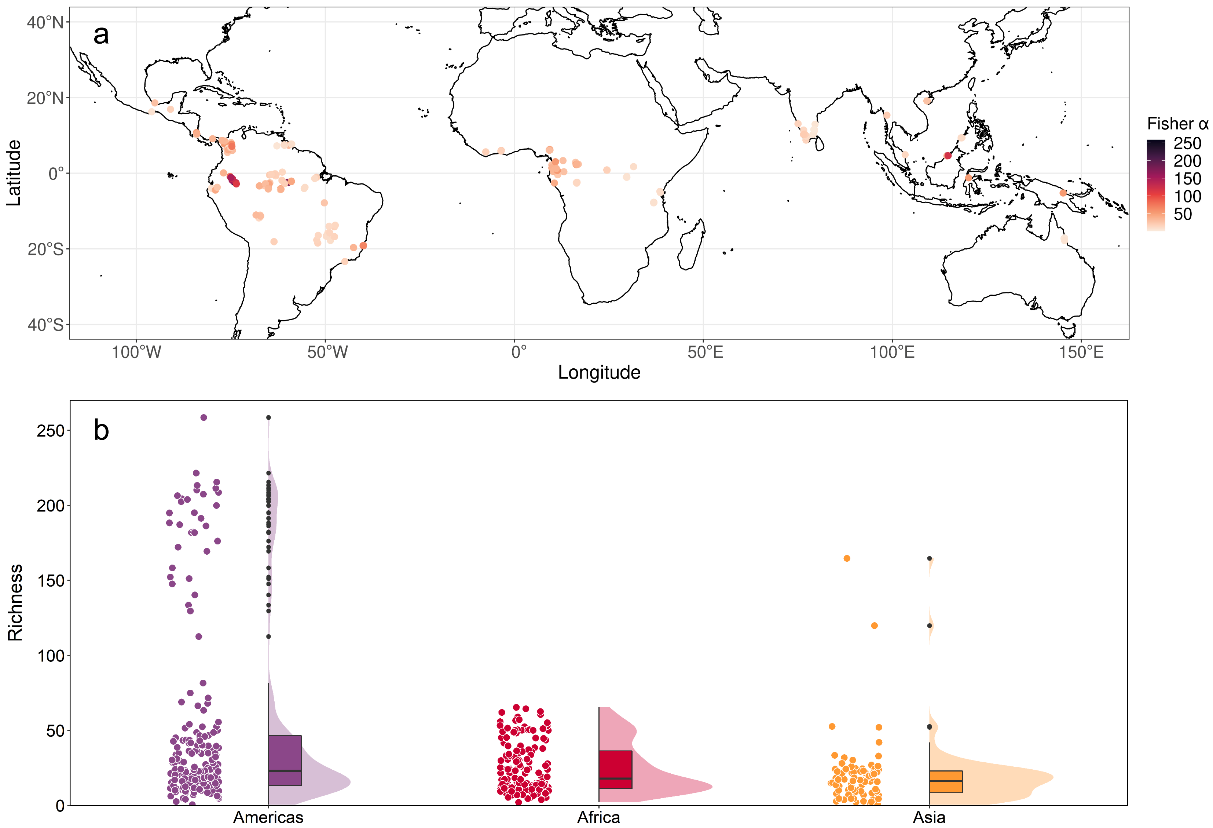


**Fig. S3.** **Tree species richness based on Fisher’s** **α in 1-ha plots across the global tropics.** (**a**) Distribution of 429 1-ha old-growth forest plots used in this study. Different colored points show tree species richness estimated from Fisher’s α. (**b**) Density distribution of the plots for the three major tropical regions. Box plots represent the median and interquartile range of Fisher’s α, alongside individual plot data. Vertical lines extend to 1.5 times the difference between quartiles and black points represent outliers. Width of the distribution represents the number of plots at a given level of Fisher’s α.

**
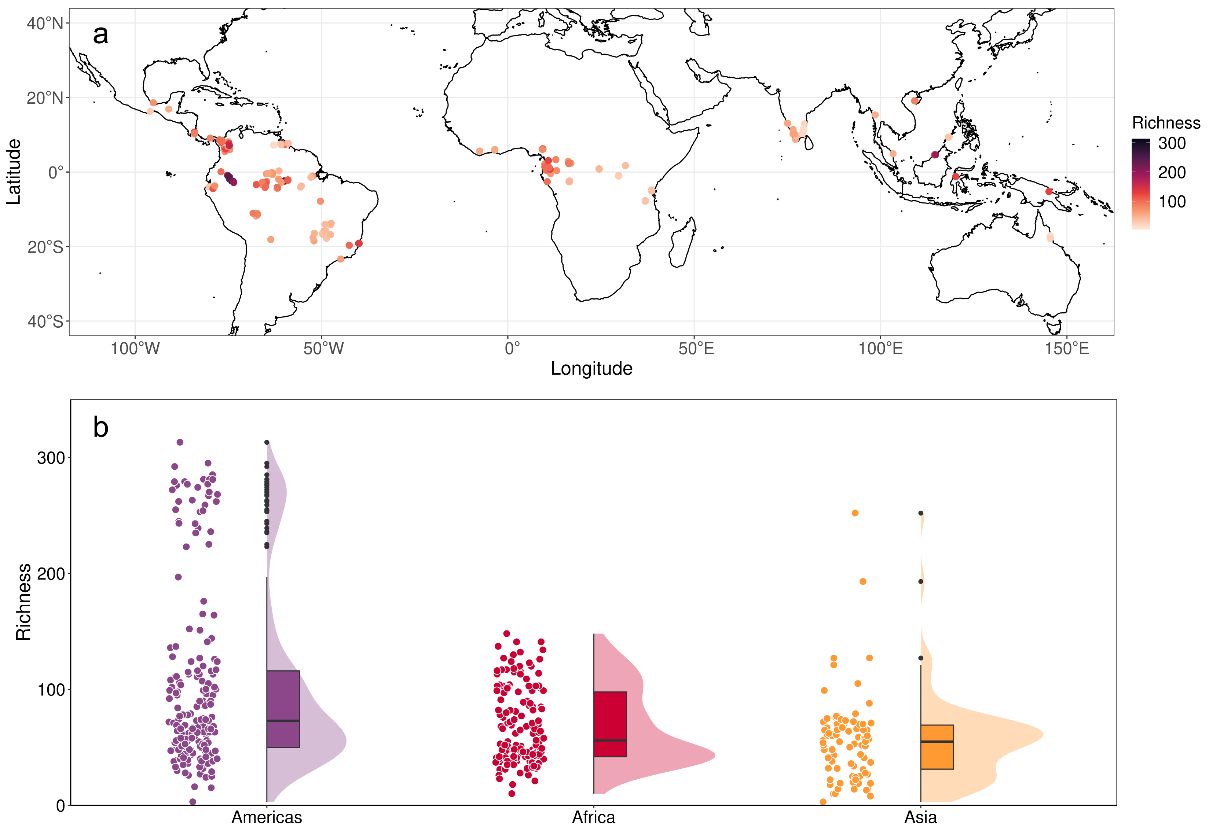
**

**Fig. S4.** **Observed tree species richness in 1-ha plots across the global tropics.** (**a**) Distribution of 429 1-ha old-growth forest plots used in this study. Different colored points show observed tree species richness. (**b**) Density distribution of the plots for the three major tropical regions. Box plots display the median and interquartile range of observed richness, alongside individual plot data. Vertical lines extend to 1.5 times the difference between quartiles and black points represent outliers. Width of the distribution represents the number of plots at a given richness level.

**
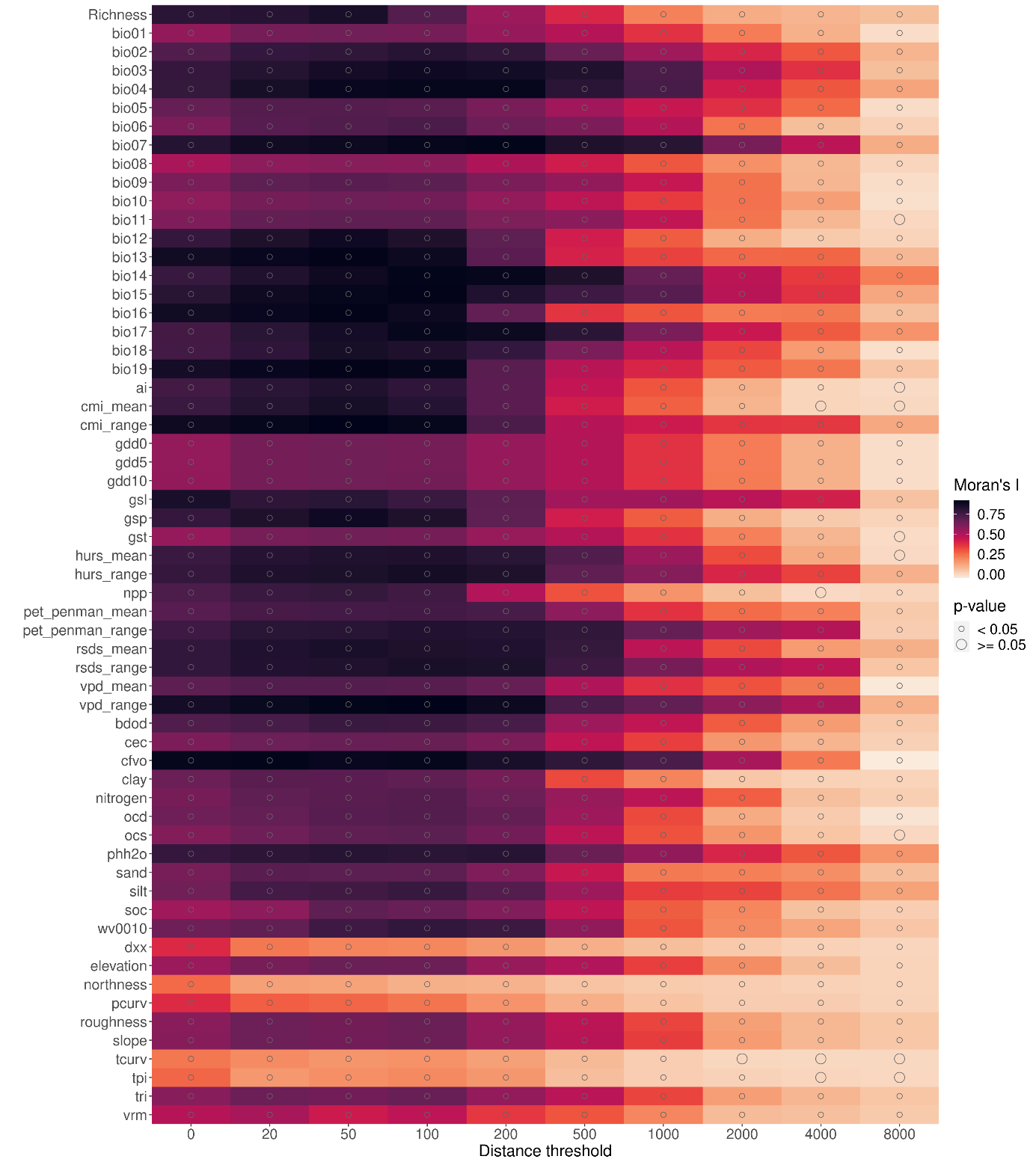
**

**Fig. S5.** **Spatial autocorrelation of tree species richness and 59 environmental predictors** **based on Moran’s index.**

**
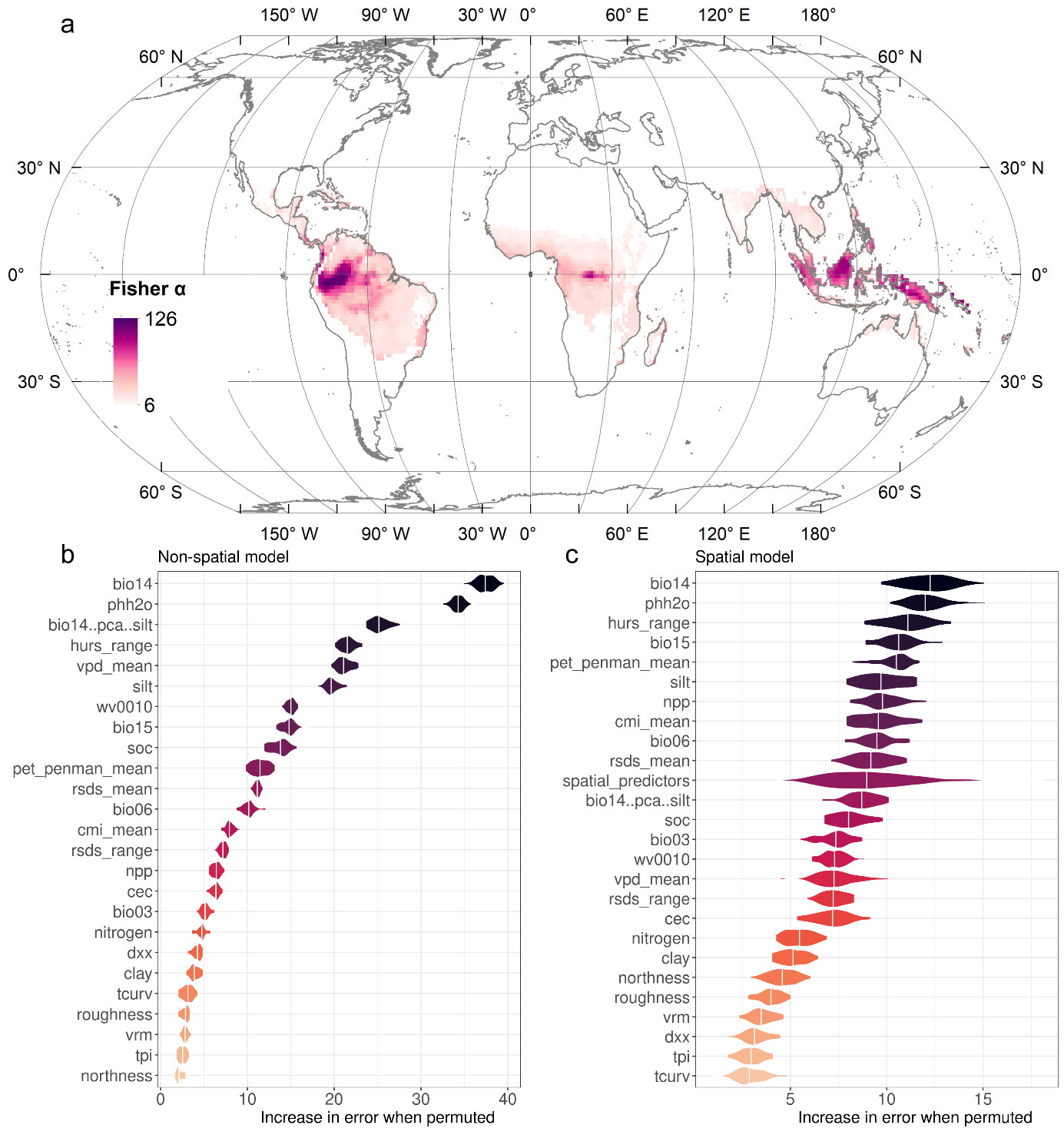
**

**Fig. S6.** **Predicted Fisher’s α at the 1-ha scale and importance of environmental variables based on non-spatial and spatial random forest (RF).** (**a**) Predicted Fisher’s α extrapolated across the tropics based on non-spatial RF. (**b**) Importance of environmental variables according to non-spatial RF, which does not account for spatial autocorrelation among forest plots. (**c**) Importance of environmental variables according to spatial RF, which does account for spatial autocorrelation among forest plots.

**
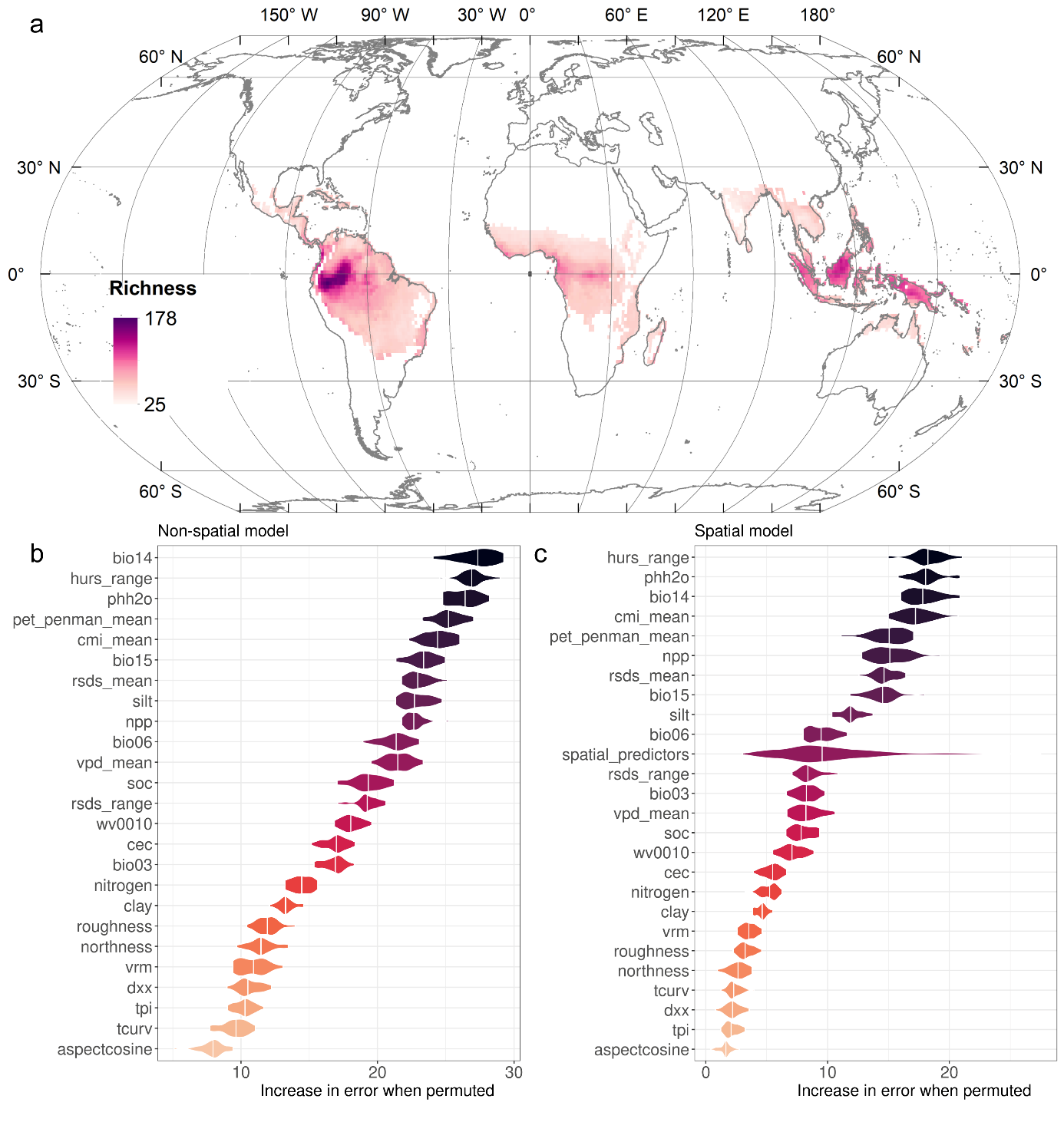
**

**Fig. S7.** **Predicted tree species richness based on observed number of species at the 1-ha scale and importance of environmental variables based on non-spatial and spatial RF.** (**a**) Predicted tree species richness extrapolated across the tropics based on non-spatial RF. (**b**) Importance of environmental variables according to non-spatial RF. (**c**) Importance of environmental variables according to spatial RF.

**
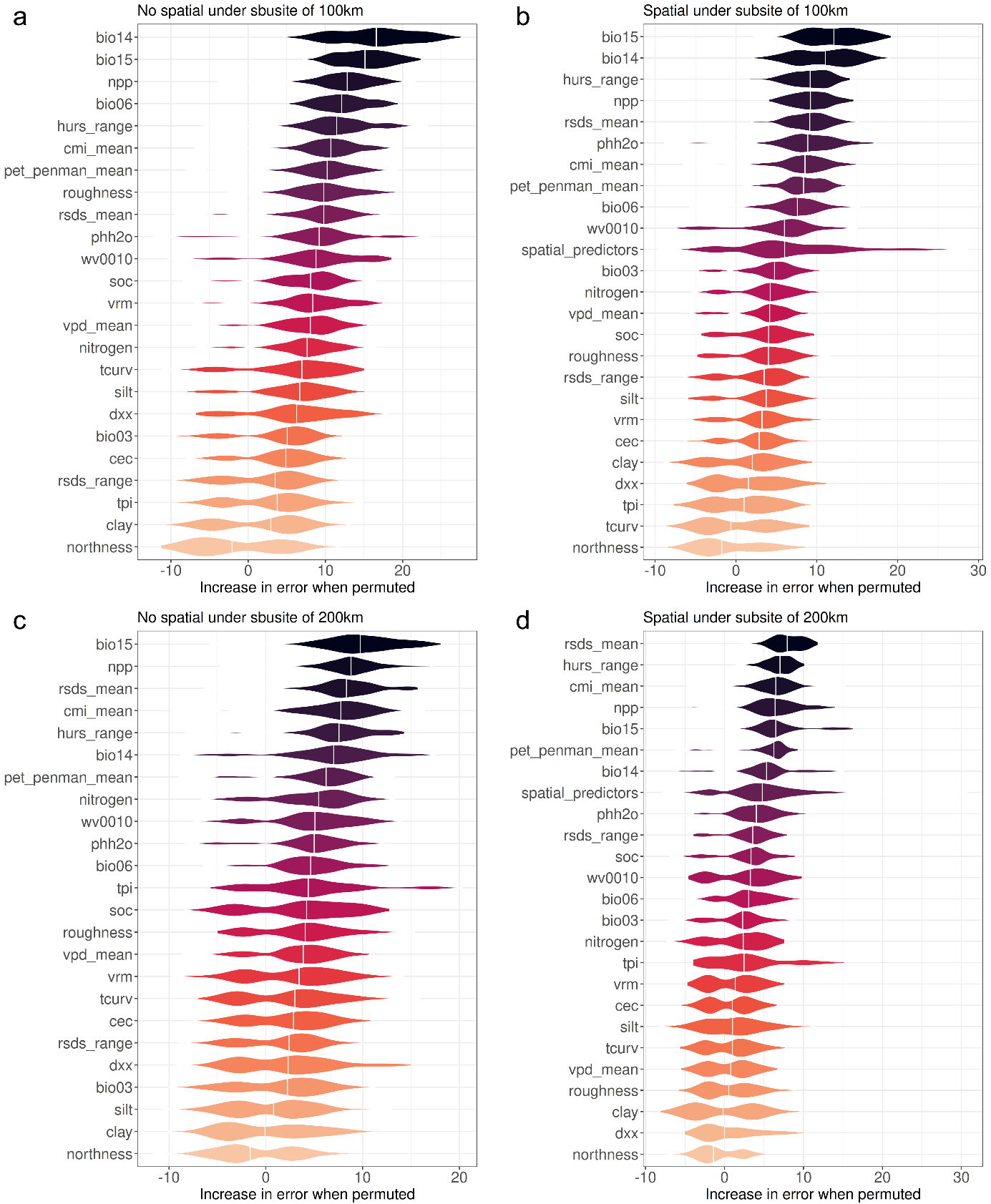
**

**Fig. S8.** **Twenty-four environmental variables ranked by importance for predicting tree species richness estimated from rarefaction according to different RF models.** (**a**) non-spatial RF on subset data with spatial thinning of 100 km, (**b**) spatial RF on subset data with spatial thinning of 100 km, (**c**) non-spatial RF on subset data with spatial thinning of 200 km, and (**d**) spatial RF on subset data with spatial thinning of 200 km.


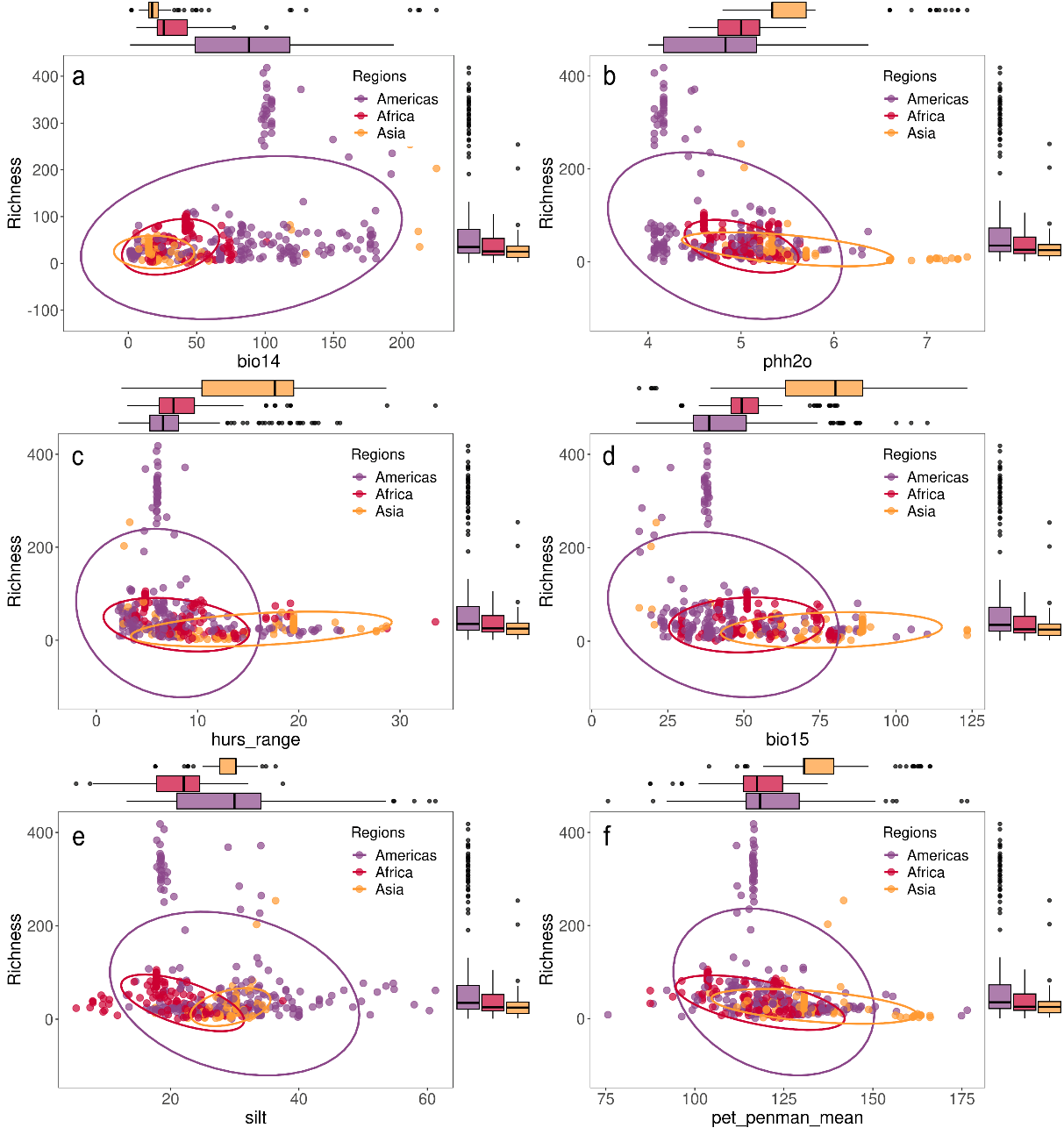


**Fig. S9.** **Relationships between six dominant environment variables and tree species richness estimated from rarefaction.** These variables are: (**a**) precipitation amount of the driest month (bio14), (**b**) soil pH (phh2o), (**c**) annual range of monthly near-surface relative humidity (hurs_range), (**d**) precipitation seasonality (bio15), (**e**) silt proportion (silt), and (**f**) mean monthly potential evapotranspiration (pet_penman_mean). Note that these are a fraction of the environmental variables considered by the RF model.


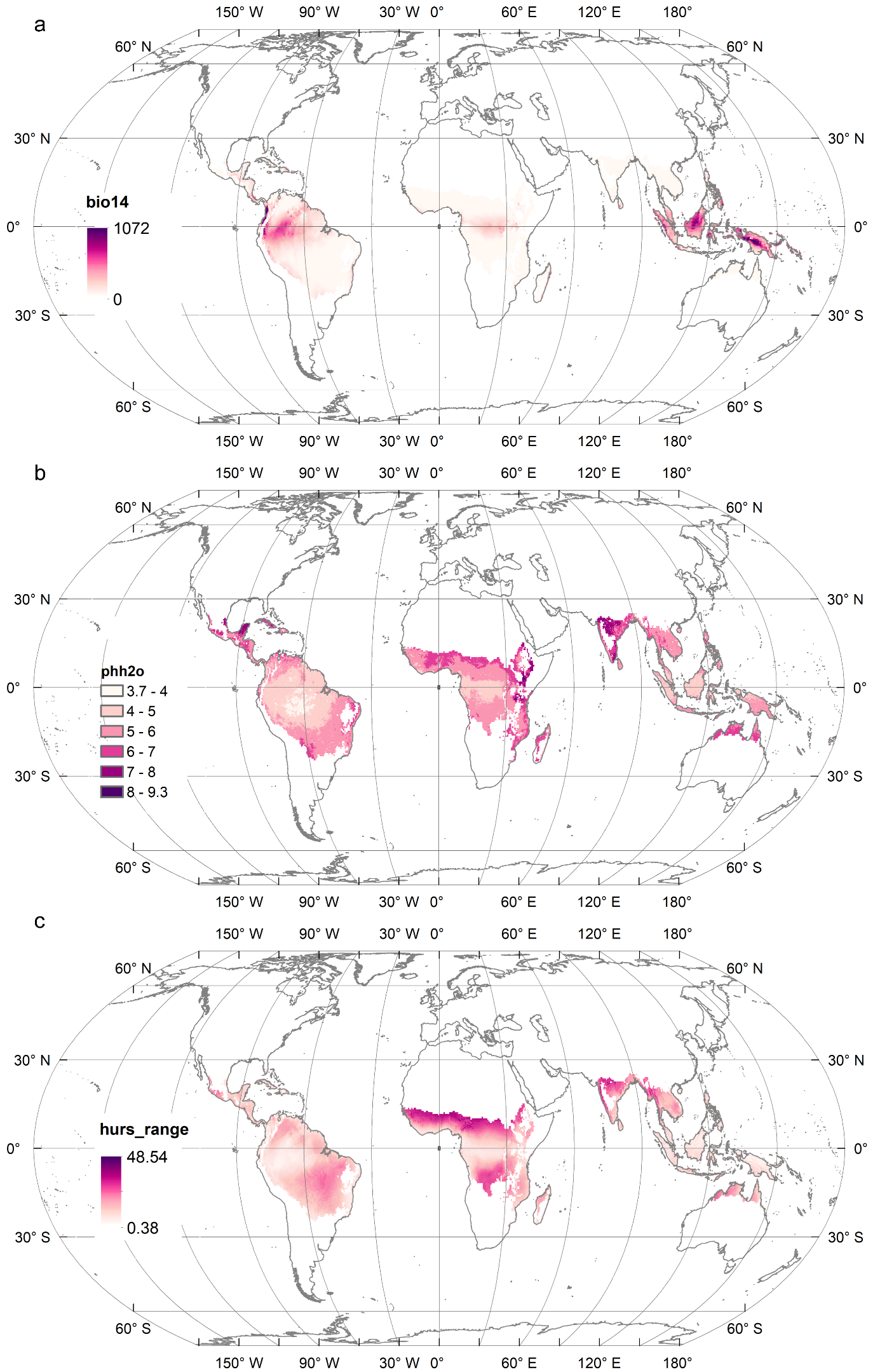


**Fig. S10.** **Spatial variation of important environmental variables extrapolated across the tropics based on spatial RF.** (**a**) precipitation of the driest month (bio14), (**b**) soil pH (phh2o), and (**c**) annual range of relative humidity (hurs_range).


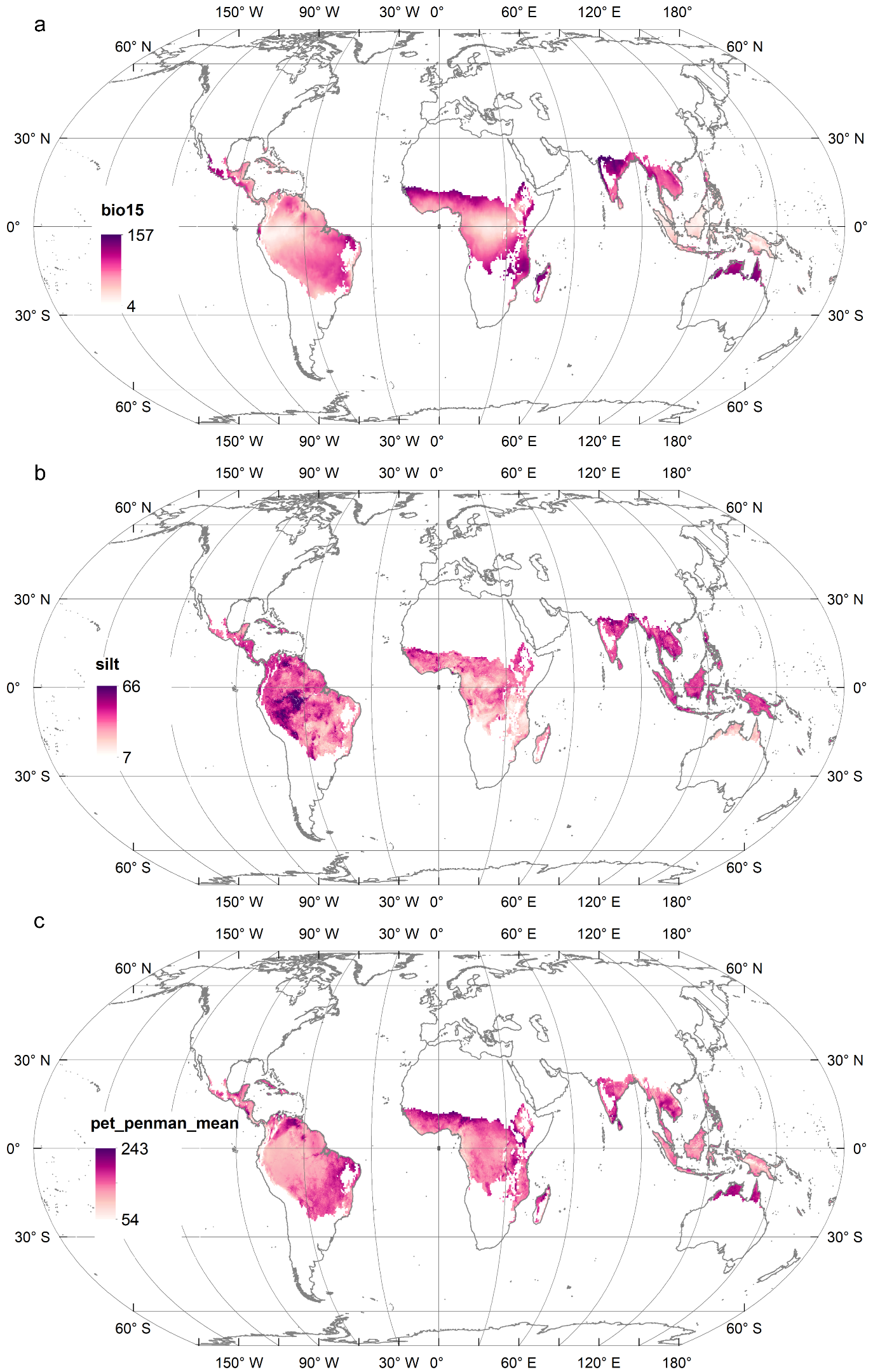


**Fig. S11.** **Spatial variation of important environmental variables extrapolated across the tropics based on spatial RF.** (**a**) precipitation seasonality (bio15), (**b**) proportion of silt particles in fine earth fraction (silt), and (**c**) mean monthly potential evapotranspiration (pet_penman_mean).


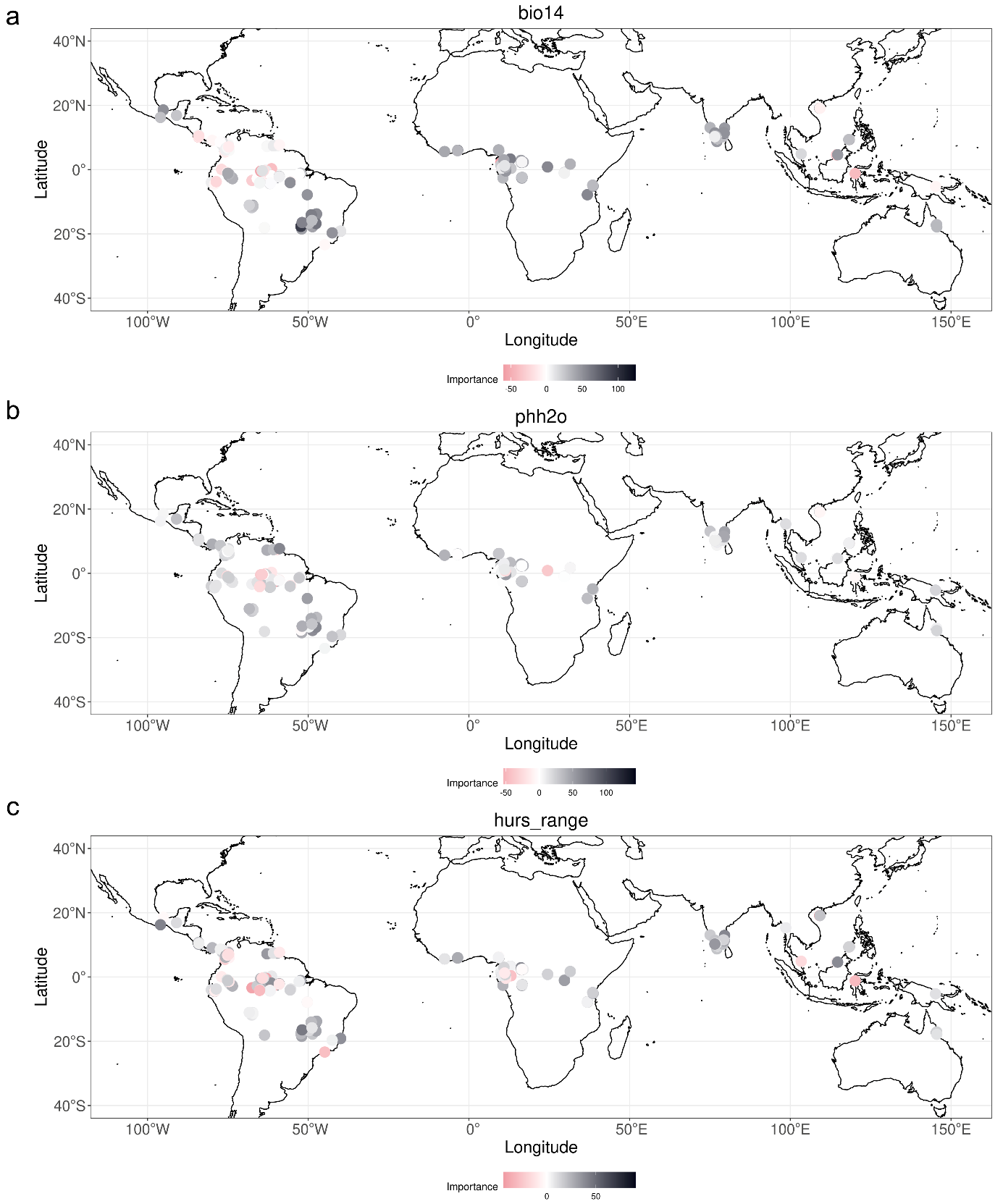


**Fig. S12.** **Importance of key environmental variables based on permutation error in 429 1-ha old-growth tropical forest plots.** (**a**) precipitation of the driest month (bio14), (**b**) soil pH (phh2o), and (**c**) annual range of relative humidity (hurs_range).


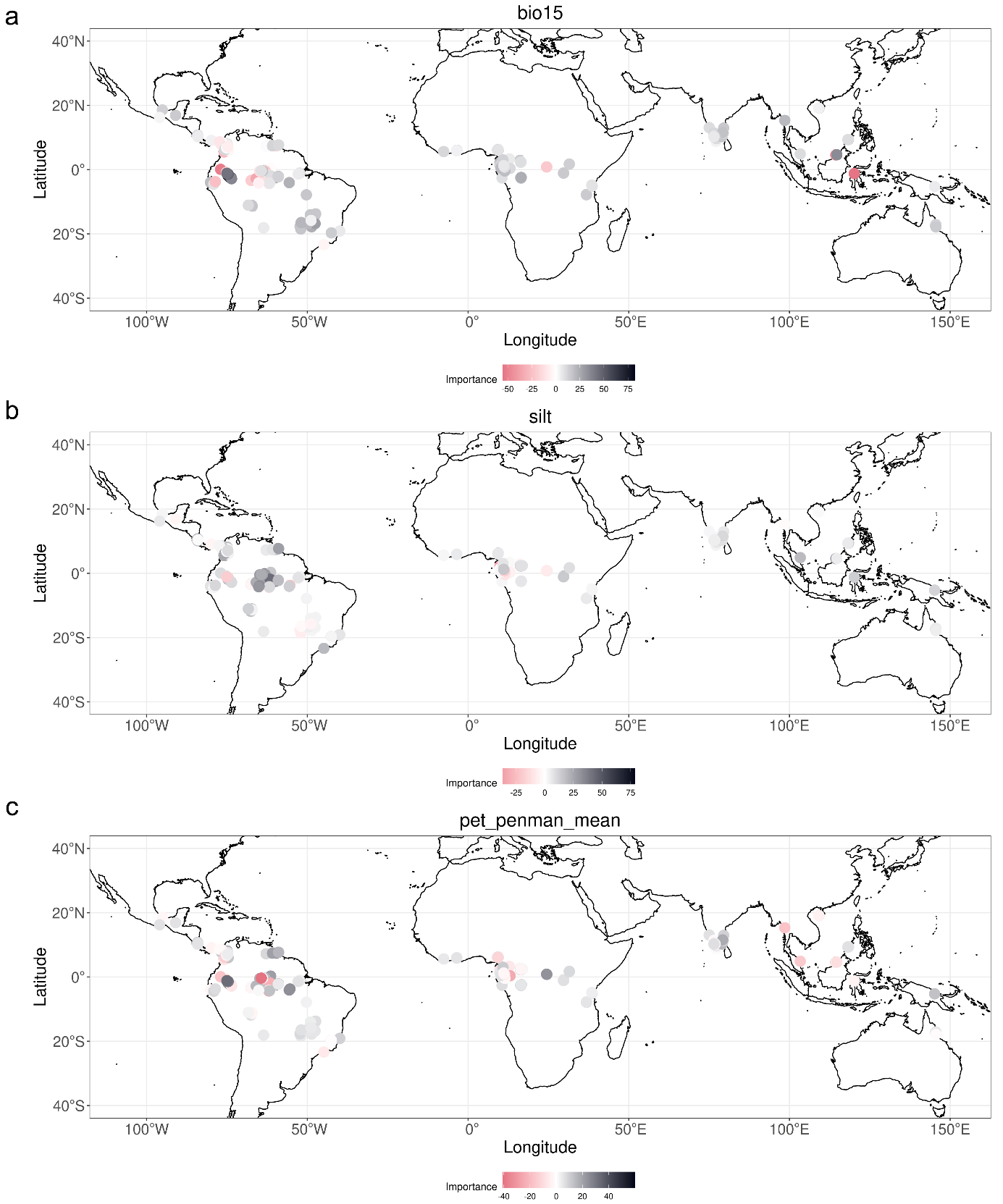


**Fig. S13.** **Importance of key environmental variables based on permutation error in 429 1-ha old-growth tropical forest plots.** (**a**) precipitation seasonality (bio15), (**b**) silt proportion (silt), and (**c**) mean monthly potential evapotranspiration (pet_penman_mean).


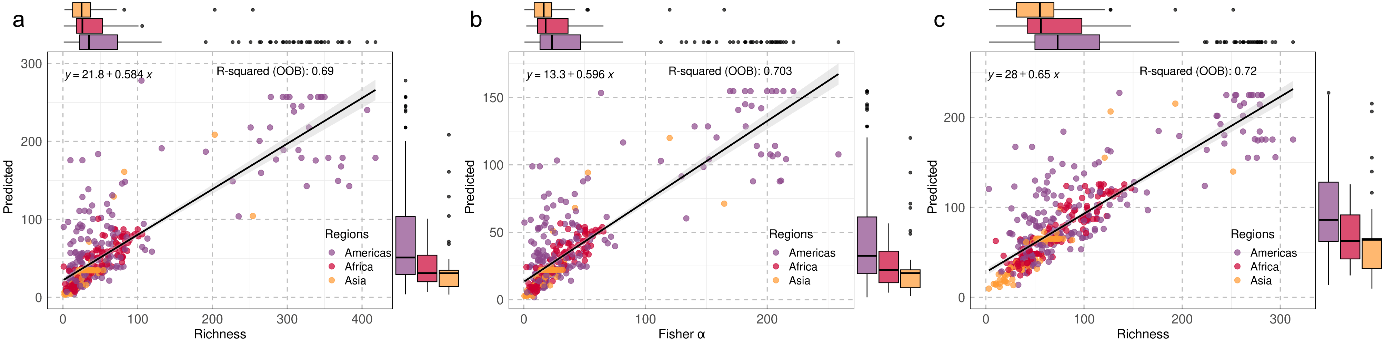


**Fig. S14.** **Comparison of simple total richness per sample and predicted richness using regression analysis.** These predictions are made by glm.nb models. **(A)** glm.nb model based on rarefaction estimated richness, **(B)** glm.nb model based on Fisher’s alpha, **(C)** glm.nb model based on observed richness.


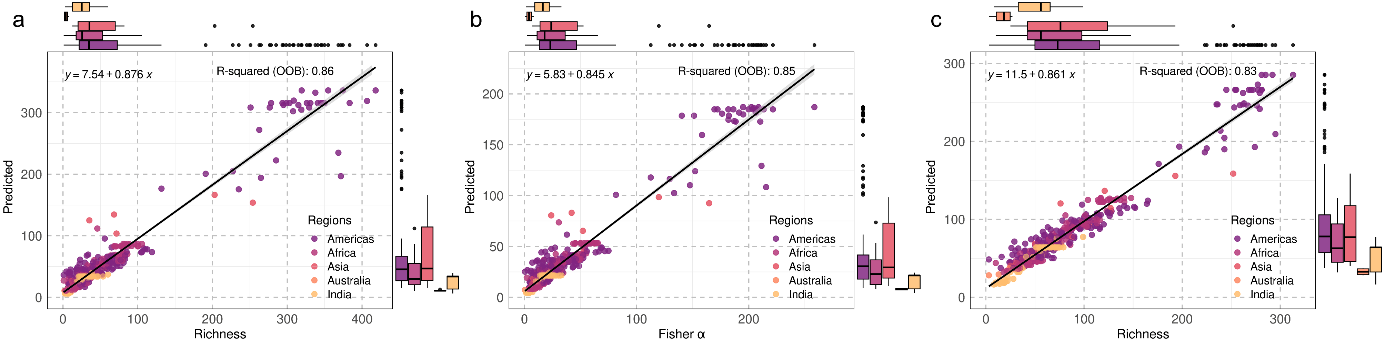


**Fig. S15.** **Comparison of simple total richness per sample and predicted richness using regression analysis, broken down into five regions.** Predictions are made by RF models. **(a)** RF model based on rarefaction estimated richness, **(b)** RF model based on Fisher’s α, **(c)** RF model based on observed richness.


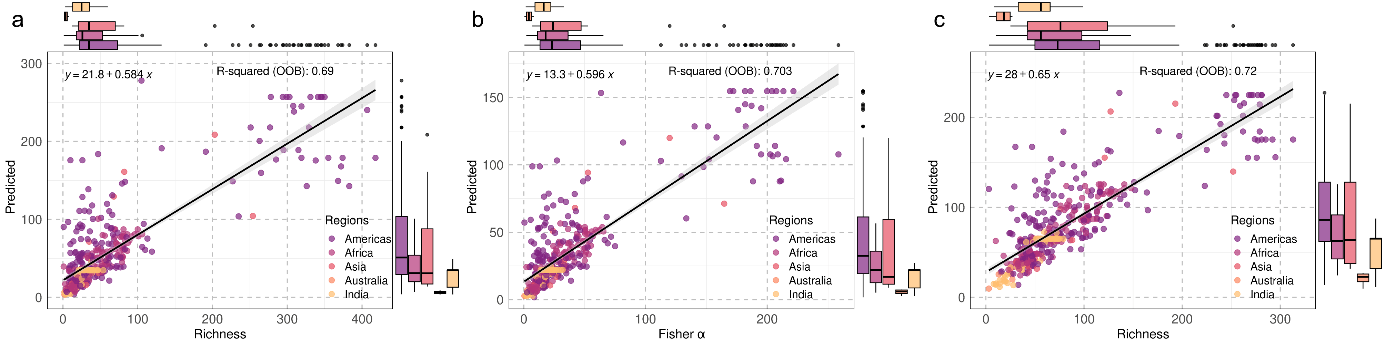


**Fig. S16.** **Comparison of simple total richness per sample and predicted richness using regression analysis, broken down into five regions.** Predictions are made by glm.nb models, respectively. **(a)** glm.nb model based on rarefaction estimated richness, **(b)** glm.nb model based on Fisher’s α, **(c)** glm.nb model based on observed richness.


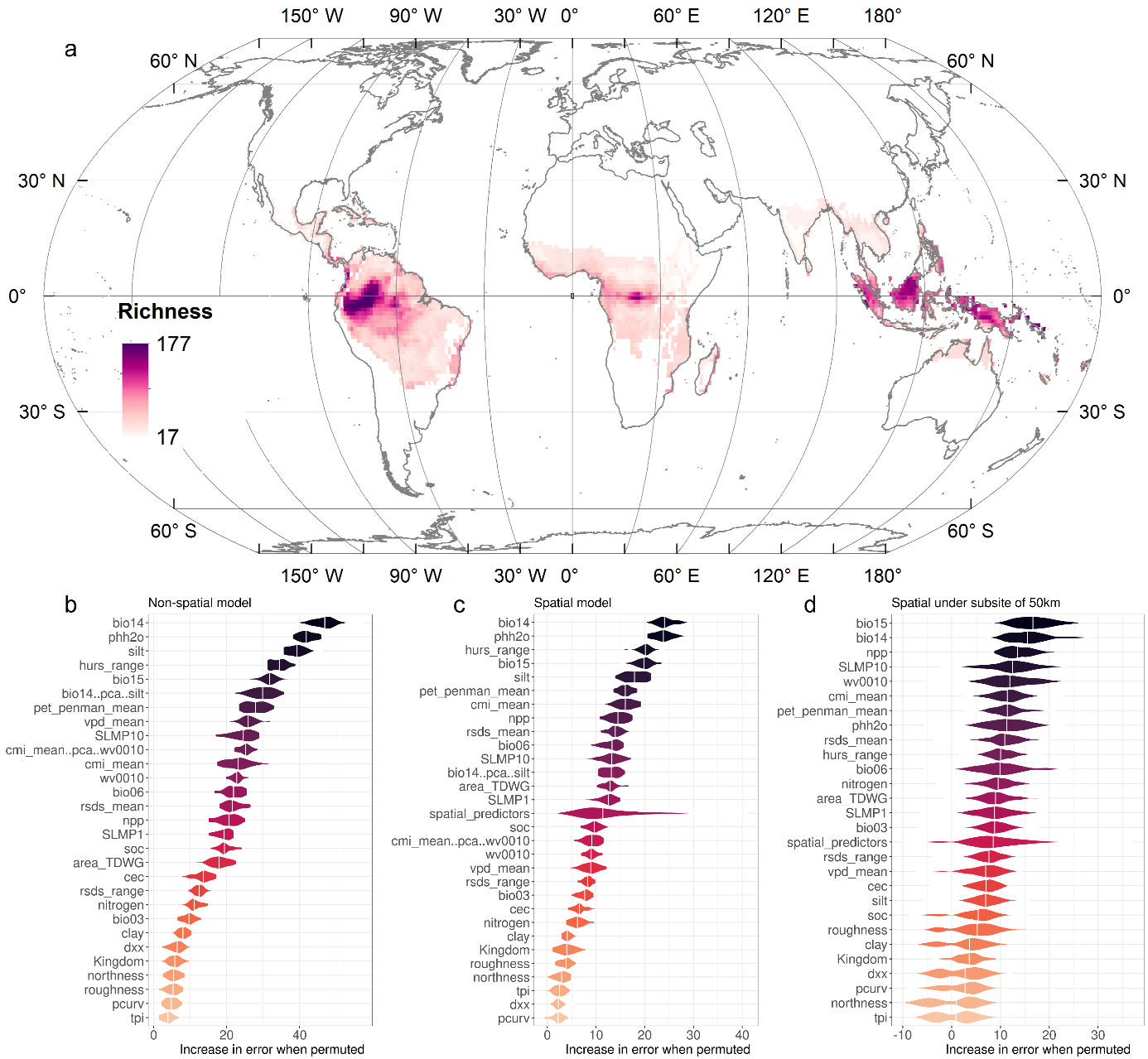


**Fig. S17. Incorporating species pools to predict tree species richness at the 1-ha scale and importance of environmental variables based on non-spatial and spatial random forest (RF).** **(a)** Local tree species richness estimated from rarefaction and predicted using non-spatial RF across the tropics. **(b)** Importance of environmental variables according to non-spatial RF, which does not account for spatial autocorrelation among forest plots. **(b)** Importance of environmental variables according to spatial RF, which accounts for spatial autocorrelation among forest plots. **(d)** Importance of environmental variables according to spatial RF with thinning of samples by 50 km.


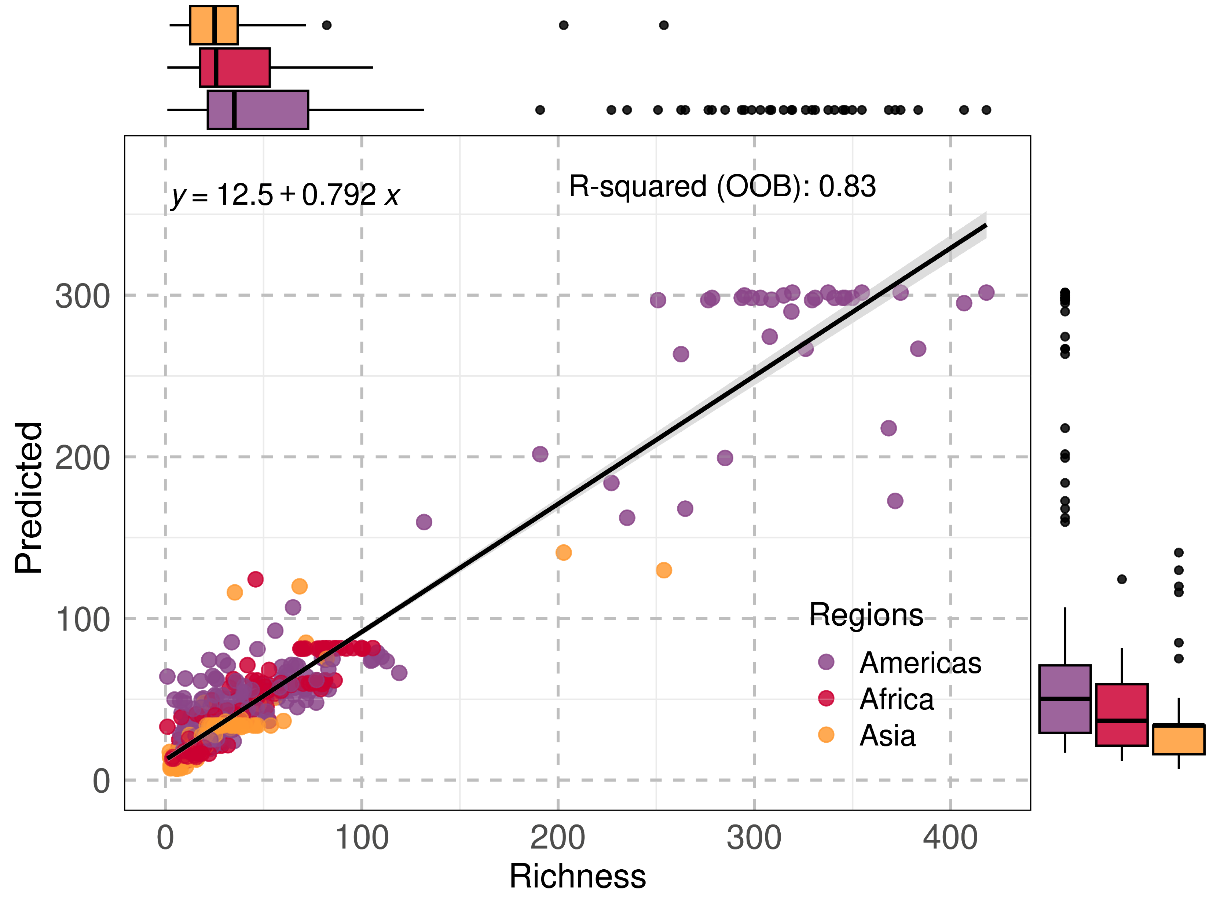


**Fig. S18.** **Predicted tree species richness (sample-based rarefaction) according to spatial RF based on 24 environmental variables and incorporating species pools.**


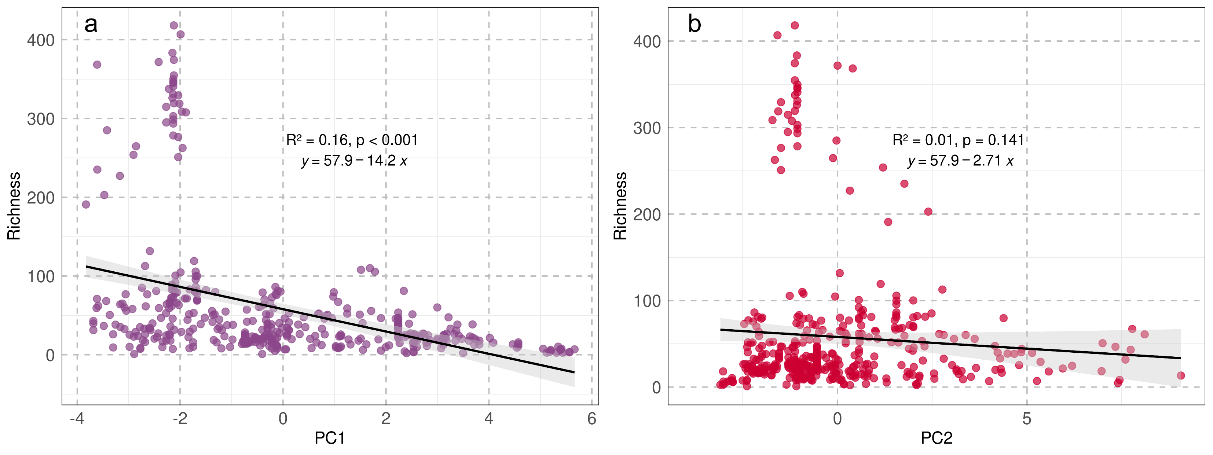


**Fig. S19.** **The line regression between two principal components with Richness.** **(a)** PC1 VS Richness, **(b)** PC2 VS Richness.

**Supplementary Tables**

**Table S1. Category, description and short name of 24 environmental variables used in random forest and negative binomial generalized linear models**

| Category | Variable description | Short name |
| --- | --- | --- |
| Temperature | Isothermality | bio03 |
|  | Mean daily minimum air temperature of the coldest month | bio06 |
| Precipitation | Precipitation amount of the driest month | bio14 |
|  | Precipitation seasonality | bio15 |
|  | Mean monthly climate moisture index | cmi_mean |
|  | Annual range of monthly near surface relative humidity | hurs_range |
|  | Mean monthly potential evapotranspiration | pet_penman_mean |
|  | Mean monthly vapor pressure deficit | vpd_mean |
| Grow season | Net primary productivity | npp |
| Solar radiation | Mean monthly surface downwelling shortwave flux in air | rsds_mean |
|  | Annual range of monthly surface downwelling shortwave flux in air | rsds_range |
| Soil | Cation exchange capacity of the soil | cec |
|  | Proportion of clay particles (< 0.002 mm) in the fine earth fraction | clay |
|  | Total nitrogen (N) | nitrogen |
|  | Soil pH | phh2o |
|  | Proportion of silt particles (≥ 0.002 mm and ≤ 0.05 mm) in the fine earth fraction | silt |
|  | Soil organic carbon content in the fine earth fraction | soc |
|  | Volumetric water content at 10 kPa in 10^-3^ cm3cm-3 | wv0010 |
| Topography | Second order partial derivative (E-W slope) | dxx |
|  | Northness | northness |
|  | Roughness | roughness |
|  | Tangential curvature | tcurv |
|  | Topographic position index | tpi |
|  | Vector ruggedness measure | vrm |

**Table S2.** **Results of the principal component analysis load**

|  | Dim.1 | Dim.2 | Dim.3 | Dim.4 | Dim.5 |
| --- | --- | --- | --- | --- | --- |
| bio03 | -7.42e-01 | -5.66e-02 | -3.46e-01 | 1.39e-01 | -1.83e-01 |
| bio06 | -5.09e-01 | -6.44e-01 | 2.91e-01 | 1.45e-01 | 1.22e-01 |
| bio14 | -7.39e-01 | 2.09e-01 | 1.44e-01 | 3.72e-01 | 5.42e-02 |
| bio15 | 7.97e-01 | -2.74e-02 | 1.81e-01 | -3.44e-01 | 2.70e-01 |
| cmi_mean | -3.96e-01 | 4.31e-01 | 5.73e-01 | -2.21e-01 | 3.82e-01 |
| hurs_range | 7.24e-01 | 2.19e-02 | 2.72e-01 | -2.42e-01 | 3.01e-01 |
| npp | -5.05e-01 | -3.54e-02 | 5.39e-01 | -1.73e-01 | 4.79e-01 |
| pet_penman_mean | 5.79e-01 | -4.89e-01 | -5.20e-03 | 4.92e-01 | 4.88e-02 |
| rsds_mean | 6.52e-01 | -2.25e-01 | -1.36e-01 | 4.11e-01 | -2.43e-02 |
| rsds_range | 5.30e-01 | 2.24e-01 | 5.38e-01 | -5.58e-02 | 2.93e-01 |
| vpd_mean | 3.95e-01 | -7.30e-01 | 1.88e-01 | 3.17e-01 | 1.30e-01 |
| cec | 2.20e-01 | 4.51e-01 | 8.92e-02 | 6.18e-01 | -1.09e-01 |
| clay | -1.00e-01 | 6.65e-02 | -1.86e-01 | -2.51e-01 | -1.23e-01 |
| nitrogen | 3.53e-02 | 8.09e-01 | 2.04e-01 | 2.46e-01 | -9.51e-02 |
| phh2o | 8.08e-01 | 2.69e-02 | -1.36e-02 | 3.01e-01 | -9.71e-02 |
| silt | -1.19e-01 | 2.50e-01 | 3.17e-01 | 5.99e-01 | 2.63e-01 |
| soc | -2.04e-01 | 6.76e-01 | 1.49e-01 | 4.52e-01 | -4.69e-02 |
| wv0010 | 9.30e-03 | 6.24e-01 | 1.53e-01 | 7.41e-02 | 1.39e-01 |
| dxx | -4.12e-02 | 8.53e-02 | -6.61e-01 | 1.42e-01 | 6.11e-01 |
| northness | 1.24e-01 | -1.09e-01 | 7.94e-02 | 1.49e-02 | 9.05e-02 |
| roughness | 4.03e-01 | 7.33e-01 | -8.17e-02 | -2.34e-01 | -2.31e-01 |
| tcurv | 1.05e-01 | 3.69e-01 | -6.21e-01 | -1.36e-02 | 3.46e-01 |
| tpi | -3.31e-03 | 2.69e-01 | -6.87e-01 | 4.81e-02 | 6.06e-01 |
| vrm | 3.71e-01 | 6.63e-01 | -5.64e-02 | -1.99e-01 | -2.88e-01 |

**Table S3.** **Results of the linear model (formula = Richness ~ PC1 + PC2)**

|  | Estimate | Std.Error | t value | Pr(>\|z\|) |  |
| --- | --- | --- | --- | --- | --- |
| (Intercept) | 5.79e+01 | 3.56e+00 | 1.63e+01 | 1.35e-46 | *** |
| PC1 | -1.42e+01 | 1.56e+00 | -9.09e+00 | 3.92e-18 | *** |
| PC2 | -2.71e+00 | 1.68e+00 | -1.61e+00 | 1.08e-01 |  |

**Table S4.** **Pearson’s correlation coefficients between observed species richness and seven diversity indices**

|  | richness | shannon | simp | inv.simp | richness.coverage | shannon.coverage | simp.coverage | fisher.alpha |
| --- | --- | --- | --- | --- | --- | --- | --- | --- |
| richness | 1.000 | 0.854 | 0.517 | 0.926 | 0.957 | 0.955 | 0.925 | 0.966 |
| shannon | 0.854 | 1.000 | 0.847 | 0.790 | 0.721 | 0.737 | 0.752 | 0.734 |
| simp | 0.517 | 0.847 | 1.000 | 0.462 | 0.368 | 0.386 | 0.417 | 0.378 |
| inv.simp | 0.926 | 0.790 | 0.462 | 1.000 | 0.923 | 0.962 | 0.995 | 0.937 |
| richness.coverage | 0.957 | 0.721 | 0.368 | 0.923 | 1.000 | 0.991 | 0.941 | 0.996 |
| shannon.coverage | 0.955 | 0.737 | 0.386 | 0.962 | 0.991 | 1.000 | 0.976 | 0.994 |
| simp.coverage | 0.925 | 0.752 | 0.417 | 0.995 | 0.941 | 0.976 | 1.000 | 0.954 |
| fisher.alpha | 0.966 | 0.734 | 0.378 | 0.937 | 0.996 | 0.994 | 0.954 | 1.000 |

Notes: The diversity measures are observed raw richness (richness), Shannon index (shannon), Simpson index (simp), inverse Simpson index (inv.simp), richness estimated from sample-based rarefaction (richness.coverage), Shannon estimated from sample-based rarefaction (shannon.coverage), Simpson estimated from sample-based rarefaction (simp.coverage), and Fisher’s alpha (fisher.alpha).

**Table S5. Performance of non-spatial and spatial random forest models of pantropical tree species richness estimated from sample-based rarefaction, Fisher’s α and observed richness**

| Model performance | Richness from rarefaction | | Fisher’s α | | Observed richness | |
| --- | --- | --- | --- | --- | --- | --- |
|  | Non spatial | Spatial model | Non spatial | Spatial model | Non spatial | Spatial model |
| *R*^2^ (OOB) | 0.86 | 0.88 | 0.85 | 0.87 | 0.83 | 0.86 |
| *R*^2^ (cor(obs, pred)^2^) | 0.94 | 0.94 | 0.93 | 0.94 | 0.95 | 0.94 |
| Pseudo *R*^2^ (cor(obs, pred)) | 0.97 | 0.97 | 0.96 | 0.97 | 0.97 | 0.97 |
| RMSE (OOB) | 30.29 | 27.87 | 18.36 | 16.70 | 25.80 | 23.27 |
| RMSE | 20.47 | 21.04 | 13.16 | 12.61 | 15.10 | 15.82 |
| Normalized RMSE | 0.50 | 0.51 | 0.51 | 0.49 | 0.26 | 0.28 |

Notes: *R*^2^ (OOB) refers to the “out-of-bag” *R*^2^ value, derived from observations not included in the training subset for a particular tree. Standard *R*^2^ is calculated as the square of the correlation coefficient between observed (obs) and predicted (pred) values from the model. Pseudo *R*^2^ refers to the correlation between observed and predicted values. RMSE (OOB) is the root mean square error based on out-of-bag (OOB) predictions. RMSE is a standard metric used to evaluate the performance of a model by measuring the difference between predicted and observed values. Normalized RMSE is a version of root mean square error that is scaled to make it easier to interpret and compare across different datasets or models.

**Table S6.** **Results of the negative binomial generalized linear model with Americas designated as the reference category for region. See Table S3 for variable descriptions**

| Variable | Estimate | Std.Error | zvalue | Pr(>\|z\|) |  |
| --- | --- | --- | --- | --- | --- |
| (Intercept) | 4.06e+00 | 6.54e-02 | 6.21e+01 | < 2e-16 | *** |
| RegionAfrica | -1.43e+00 | 3.06e-01 | -4.69e+00 | 2.77e-06 | *** |
| RegionAsia | -1.44e+00 | 5.76e-01 | -2.50e+00 | 1.23e-02 | * |
| bio03 | 3.69e-02 | 1.01e-01 | 3.66e-01 | 7.15e-01 |  |
| bio06 | -6.21e-01 | 1.55e-01 | -4.00e+00 | 6.45e-05 | *** |
| bio14 | -6.27e-01 | 1.29e-01 | -4.85e+00 | 1.26e-06 | *** |
| bio15 | -4.60e-01 | 1.34e-01 | -3.45e+00 | 5.70e-04 | *** |
| cmi_mean | 5.59e-01 | 1.32e-01 | 4.24e+00 | 2.19e-05 | *** |
| hurs_range | -8.93e-01 | 1.19e-01 | -7.49e+00 | 6.93e-14 | *** |
| npp | 4.51e-01 | 1.21e-01 | 3.74e+00 | 1.81e-04 | *** |
| pet_penman_mean | -3.19e-01 | 1.03e-01 | -3.08e+00 | 2.05e-03 | ** |
| rsds_mean | -6.78e-02 | 1.19e-01 | -5.69e-01 | 5.69e-01 |  |
| rsds_range | -1.65e-01 | 9.63e-02 | -1.72e+00 | 8.63e-02 | . |
| vpd_mean | 1.02e+00 | 1.55e-01 | 6.55e+00 | 5.77e-11 | *** |
| cec | -7.88e-02 | 5.67e-02 | -1.39e+00 | 1.64e-01 |  |
| clay | -2.10e-01 | 4.54e-02 | -4.62e+00 | 3.83e-06 | *** |
| nitrogen | 1.10e-01 | 6.89e-02 | 1.60e+00 | 1.09e-01 |  |
| phh2o | -2.77e-01 | 6.64e-02 | -4.18e+00 | 2.90e-05 | *** |
| silt | -4.24e-01 | 4.21e-02 | -1.01e+01 | 6.93e-24 | *** |
| soc | -9.01e-02 | 5.73e-02 | -1.57e+00 | 1.16e-01 |  |
| wv0010 | -4.10e-01 | 6.92e-02 | -5.92e+00 | 3.22e-09 | *** |
| dxx | 2.25e-02 | 1.06e-01 | 2.13e-01 | 8.31e-01 |  |
| northness | 1.44e-02 | 3.68e-02 | 3.91e-01 | 6.96e-01 |  |
| roughness | 3.48e-01 | 1.46e-01 | 2.38e+00 | 1.71e-02 | * |
| tcurv | -1.56e-01 | 8.11e-02 | -1.92e+00 | 5.46e-02 | . |
| tpi | 2.49e-01 | 1.10e-01 | 2.26e+00 | 2.38e-02 | * |
| vrm | 1.15e-01 | 1.47e-01 | 7.84e-01 | 4.33e-01 |  |
| RegionAfrica:bio03 | 3.41e-01 | 2.10e-01 | 1.63e+00 | 1.04e-01 |  |
| RegionAsia:bio03 | 2.72e-01 | 3.37e-01 | 8.06e-01 | 4.20e-01 |  |
| RegionAfrica:bio06 | 1.13e+00 | 5.22e-01 | 2.17e+00 | 2.98e-02 | * |
| RegionAsia:bio06 | 1.45e-01 | 3.75e-01 | 3.87e-01 | 6.98e-01 |  |
| RegionAfrica:bio14 | 6.14e-01 | 4.56e-01 | 1.35e+00 | 1.78e-01 |  |
| RegionAsia:bio14 | 2.37e-01 | 5.19e-01 | 4.57e-01 | 6.48e-01 |  |
| RegionAfrica:bio15 | 9.77e-01 | 4.32e-01 | 2.26e+00 | 2.37e-02 | * |
| RegionAsia:bio15 | 1.91e-01 | 4.62e-01 | 4.14e-01 | 6.79e-01 |  |
| RegionAfrica:cmi_mean | -7.94e-01 | 2.22e-01 | -3.57e+00 | 3.54e-04 | *** |
| RegionAsia:cmi_mean | -3.25e-01 | 3.64e-01 | -8.93e-01 | 3.72e-01 |  |
| RegionAfrica:hurs_range | 9.90e-01 | 2.00e-01 | 4.94e+00 | 7.69e-07 | *** |
| RegionAsia:hurs_range | 1.04e+00 | 2.54e-01 | 4.08e+00 | 4.45e-05 | *** |
| RegionAfrica:npp | -6.47e-02 | 2.64e-01 | -2.45e-01 | 8.06e-01 |  |
| RegionAsia:npp | -1.26e-01 | 3.29e-01 | -3.83e-01 | 7.02e-01 |  |
| RegionAfrica:pet_penman_mean | -1.44e-01 | 4.55e-01 | -3.17e-01 | 7.52e-01 |  |
| RegionAsia:pet_penman_mean | 1.01e-01 | 4.54e-01 | 2.22e-01 | 8.24e-01 |  |
| RegionAfrica:rsds_mean | -9.06e-03 | 4.32e-01 | -2.09e-02 | 9.83e-01 |  |
| RegionAsia:rsds_mean | 3.22e-01 | 4.88e-01 | 6.60e-01 | 5.10e-01 |  |
| RegionAfrica:rsds_range | 1.06e-01 | 2.01e-01 | 5.29e-01 | 5.97e-01 |  |
| RegionAsia:rsds_range | -1.64e-01 | 4.58e-01 | -3.58e-01 | 7.21e-01 |  |
| RegionAfrica:vpd_mean | -1.68e+00 | 4.12e-01 | -4.08e+00 | 4.43e-05 | *** |
| RegionAsia:vpd_mean | -4.21e-01 | 3.63e-01 | -1.16e+00 | 2.46e-01 |  |
| RegionAfrica:cec | -3.35e-01 | 1.72e-01 | -1.94e+00 | 5.19e-02 | . |
| RegionAsia:cec | -7.59e-02 | 1.98e-01 | -3.83e-01 | 7.02e-01 |  |
| RegionAfrica:clay | 2.44e-01 | 8.97e-02 | 2.72e+00 | 6.54e-03 | ** |
| RegionAsia:clay | 2.93e-01 | 2.75e-01 | 1.07e+00 | 2.85e-01 |  |
| RegionAfrica:nitrogen | 4.51e-01 | 2.74e-01 | 1.65e+00 | 9.95e-02 | . |
| RegionAsia:nitrogen | 9.51e-02 | 3.13e-01 | 3.04e-01 | 7.61e-01 |  |
| RegionAfrica:phh2o | 1.02e+00 | 2.61e-01 | 3.92e+00 | 8.76e-05 | *** |
| RegionAsia:phh2o | 5.28e-01 | 3.51e-01 | 1.50e+00 | 1.33e-01 |  |
| RegionAfrica:silt | 2.96e-01 | 1.76e-01 | 1.68e+00 | 9.31e-02 | . |
| RegionAsia:silt | 7.44e-01 | 3.31e-01 | 2.25e+00 | 2.45e-02 | * |
| RegionAfrica:soc | -2.55e-01 | 3.27e-01 | -7.80e-01 | 4.36e-01 |  |
| RegionAsia:soc | 3.73e-01 | 2.16e-01 | 1.72e+00 | 8.52e-02 | . |
| RegionAfrica:wv0010 | 1.15e-01 | 1.50e-01 | 7.63e-01 | 4.45e-01 |  |
| RegionAsia:wv0010 | 9.52e-01 | 2.83e-01 | 3.37e+00 | 7.65e-04 | *** |
| RegionAfrica:dxx | 6.64e-02 | 1.51e-01 | 4.41e-01 | 6.59e-01 |  |
| RegionAsia:dxx | -1.43e-01 | 2.08e-01 | -6.88e-01 | 4.91e-01 |  |
| RegionAfrica:northness | 2.75e-02 | 6.06e-02 | 4.54e-01 | 6.50e-01 |  |
| RegionAsia:northness | -7.21e-02 | 1.49e-01 | -4.83e-01 | 6.29e-01 |  |
| RegionAfrica:roughness | -4.07e-01 | 2.28e-01 | -1.78e+00 | 7.44e-02 | . |
| RegionAsia:roughness | -9.16e-02 | 3.17e-01 | -2.89e-01 | 7.73e-01 |  |
| RegionAfrica:tcurv | 2.47e-01 | 1.16e-01 | 2.13e+00 | 3.36e-02 | * |
| RegionAsia:tcurv | 2.41e-01 | 1.50e-01 | 1.61e+00 | 1.08e-01 |  |
| RegionAfrica:tpi | -2.89e-01 | 1.79e-01 | -1.62e+00 | 1.05e-01 |  |
| RegionAsia:tpi | -2.69e-01 | 2.42e-01 | -1.11e+00 | 2.66e-01 |  |
| RegionAfrica:vrm | -1.32e-01 | 2.01e-01 | -6.58e-01 | 5.10e-01 |  |
| RegionAsia:vrm | -3.41e-01 | 2.46e-01 | -1.39e+00 | 1.66e-01 |  |

**Table S7.** **Results of the negative binomial generalized linear model with Africa designated as the reference category for region. See Table S3 for variable descriptions**

| Variable | Estimate | Std.error | zvalue | Pr(>\|z\|) |  |
| --- | --- | --- | --- | --- | --- |
| (Intercept) | 2.63e+00 | 2.98e-01 | 8.81e+00 | 1.21e-18 | *** |
| RegionAmericas | 1.43e+00 | 3.06e-01 | 4.69e+00 | 2.77e-06 | *** |
| RegionAsia | -8.70e-03 | 6.45e-01 | -1.35e-02 | 9.89e-01 |  |
| bio03 | 3.78e-01 | 1.84e-01 | 2.05e+00 | 4.01e-02 | * |
| bio06 | 5.14e-01 | 4.99e-01 | 1.03e+00 | 3.03e-01 |  |
| bio14 | -1.26e-02 | 4.37e-01 | -2.87e-02 | 9.77e-01 |  |
| bio15 | 5.17e-01 | 4.11e-01 | 1.26e+00 | 2.08e-01 |  |
| cmi_mean | -2.35e-01 | 1.79e-01 | -1.31e+00 | 1.89e-01 |  |
| hurs_range | 9.73e-02 | 1.61e-01 | 6.04e-01 | 5.46e-01 |  |
| npp | 3.87e-01 | 2.35e-01 | 1.65e+00 | 9.95e-02 | . |
| pet_penman_mean | -4.63e-01 | 4.43e-01 | -1.04e+00 | 2.96e-01 |  |
| rsds_mean | -7.69e-02 | 4.16e-01 | -1.85e-01 | 8.53e-01 |  |
| rsds_range | -5.89e-02 | 1.76e-01 | -3.34e-01 | 7.38e-01 |  |
| vpd_mean | -6.67e-01 | 3.82e-01 | -1.75e+00 | 8.08e-02 | . |
| cec | -4.14e-01 | 1.63e-01 | -2.54e+00 | 1.10e-02 | * |
| clay | 3.43e-02 | 7.74e-02 | 4.44e-01 | 6.57e-01 |  |
| nitrogen | 5.62e-01 | 2.65e-01 | 2.12e+00 | 3.42e-02 | * |
| phh2o | 7.45e-01 | 2.52e-01 | 2.96e+00 | 3.12e-03 | ** |
| silt | -1.28e-01 | 1.71e-01 | -7.49e-01 | 4.54e-01 |  |
| soc | -3.45e-01 | 3.22e-01 | -1.07e+00 | 2.84e-01 |  |
| wv0010 | -2.95e-01 | 1.33e-01 | -2.21e+00 | 2.68e-02 | * |
| dxx | 8.89e-02 | 1.07e-01 | 8.28e-01 | 4.08e-01 |  |
| northness | 4.19e-02 | 4.82e-02 | 8.69e-01 | 3.85e-01 |  |
| roughness | -5.90e-02 | 1.75e-01 | -3.36e-01 | 7.37e-01 |  |
| tcurv | 9.10e-02 | 8.32e-02 | 1.09e+00 | 2.74e-01 |  |
| tpi | -4.04e-02 | 1.41e-01 | -2.87e-01 | 7.74e-01 |  |
| vrm | -1.69e-02 | 1.37e-01 | -1.23e-01 | 9.02e-01 |  |
| RegionAmericas:bio03 | -3.41e-01 | 2.10e-01 | -1.63e+00 | 1.04e-01 |  |
| RegionAsia:bio03 | -6.97e-02 | 3.71e-01 | -1.88e-01 | 8.51e-01 |  |
| RegionAmericas:bio06 | -1.13e+00 | 5.22e-01 | -2.17e+00 | 2.98e-02 | * |
| RegionAsia:bio06 | -9.89e-01 | 6.04e-01 | -1.64e+00 | 1.01e-01 |  |
| RegionAmericas:bio14 | -6.14e-01 | 4.56e-01 | -1.35e+00 | 1.78e-01 |  |
| RegionAsia:bio14 | -3.77e-01 | 6.66e-01 | -5.65e-01 | 5.72e-01 |  |
| RegionAmericas:bio15 | -9.77e-01 | 4.32e-01 | -2.26e+00 | 2.37e-02 | * |
| RegionAsia:bio15 | -7.86e-01 | 6.04e-01 | -1.30e+00 | 1.93e-01 |  |
| RegionAmericas:cmi_mean | 7.94e-01 | 2.22e-01 | 3.57e+00 | 3.54e-04 | *** |
| RegionAsia:cmi_mean | 4.69e-01 | 3.84e-01 | 1.22e+00 | 2.21e-01 |  |
| RegionAmericas:hurs_range | -9.90e-01 | 2.00e-01 | -4.94e+00 | 7.69e-07 | *** |
| RegionAsia:hurs_range | 4.80e-02 | 2.76e-01 | 1.74e-01 | 8.62e-01 |  |
| RegionAmericas:npp | 6.47e-02 | 2.64e-01 | 2.45e-01 | 8.06e-01 |  |
| RegionAsia:npp | -6.15e-02 | 3.86e-01 | -1.59e-01 | 8.74e-01 |  |
| RegionAmericas:pet_penman_mean | 1.44e-01 | 4.55e-01 | 3.17e-01 | 7.52e-01 |  |
| RegionAsia:pet_penman_mean | 2.45e-01 | 6.26e-01 | 3.91e-01 | 6.96e-01 |  |
| RegionAmericas:rsds_mean | 9.06e-03 | 4.32e-01 | 2.09e-02 | 9.83e-01 |  |
| RegionAsia:rsds_mean | 3.31e-01 | 6.30e-01 | 5.26e-01 | 5.99e-01 |  |
| RegionAmericas:rsds_range | -1.06e-01 | 2.01e-01 | -5.29e-01 | 5.97e-01 |  |
| RegionAsia:rsds_range | -2.70e-01 | 4.81e-01 | -5.61e-01 | 5.75e-01 |  |
| RegionAmericas:vpd_mean | 1.68e+00 | 4.12e-01 | 4.08e+00 | 4.43e-05 | *** |
| RegionAsia:vpd_mean | 1.26e+00 | 5.04e-01 | 2.51e+00 | 1.21e-02 | * |
| RegionAmericas:cec | 3.35e-01 | 1.72e-01 | 1.94e+00 | 5.19e-02 | . |
| RegionAsia:cec | 2.59e-01 | 2.50e-01 | 1.03e+00 | 3.01e-01 |  |
| RegionAmericas:clay | -2.44e-01 | 8.97e-02 | -2.72e+00 | 6.54e-03 | ** |
| RegionAsia:clay | 4.95e-02 | 2.82e-01 | 1.76e-01 | 8.61e-01 |  |
| RegionAmericas:nitrogen | -4.51e-01 | 2.74e-01 | -1.65e+00 | 9.95e-02 | . |
| RegionAsia:nitrogen | -3.56e-01 | 4.04e-01 | -8.82e-01 | 3.78e-01 |  |
| RegionAmericas:phh2o | -1.02e+00 | 2.61e-01 | -3.92e+00 | 8.76e-05 | *** |
| RegionAsia:phh2o | -4.95e-01 | 4.27e-01 | -1.16e+00 | 2.47e-01 |  |
| RegionAmericas:silt | -2.96e-01 | 1.76e-01 | -1.68e+00 | 9.31e-02 | . |
| RegionAsia:silt | 4.48e-01 | 3.70e-01 | 1.21e+00 | 2.26e-01 |  |
| RegionAmericas:soc | 2.55e-01 | 3.27e-01 | 7.80e-01 | 4.36e-01 |  |
| RegionAsia:soc | 6.27e-01 | 3.83e-01 | 1.64e+00 | 1.02e-01 |  |
| RegionAmericas:wv0010 | -1.15e-01 | 1.50e-01 | -7.63e-01 | 4.45e-01 |  |
| RegionAsia:wv0010 | 8.37e-01 | 3.05e-01 | 2.75e+00 | 6.05e-03 | ** |
| RegionAmericas:dxx | -6.64e-02 | 1.51e-01 | -4.41e-01 | 6.59e-01 |  |
| RegionAsia:dxx | -2.09e-01 | 2.09e-01 | -1.00e+00 | 3.16e-01 |  |
| RegionAmericas:northness | -2.75e-02 | 6.06e-02 | -4.54e-01 | 6.50e-01 |  |
| RegionAsia:northness | -9.96e-02 | 1.53e-01 | -6.53e-01 | 5.14e-01 |  |
| RegionAmericas:roughness | 4.07e-01 | 2.28e-01 | 1.78e+00 | 7.44e-02 | . |
| RegionAsia:roughness | 3.15e-01 | 3.31e-01 | 9.51e-01 | 3.41e-01 |  |
| RegionAmericas:tcurv | -2.47e-01 | 1.16e-01 | -2.13e+00 | 3.36e-02 | * |
| RegionAsia:tcurv | -5.51e-03 | 1.51e-01 | -3.64e-02 | 9.71e-01 |  |
| RegionAmericas:tpi | 2.89e-01 | 1.79e-01 | 1.62e+00 | 1.05e-01 |  |
| RegionAsia:tpi | 2.00e-02 | 2.57e-01 | 7.79e-02 | 9.38e-01 |  |
| RegionAmericas:vrm | 1.32e-01 | 2.01e-01 | 6.58e-01 | 5.10e-01 |  |
| RegionAsia:vrm | -2.09e-01 | 2.40e-01 | -8.71e-01 | 3.84e-01 |  |

**Table S8.** **The range of environmental variables**

|  | Maximum | Median | Minmum |
| --- | --- | --- | --- |
| bio03 | 92.7 | 73.2 | 37 |
| bio06 | 24.75 | 20.95 | 4.95 |
| bio14 | 225.2 | 41.8 | 1.4 |
| bio15 | 123.4 | 49.3 | 14.5 |
| cmi_mean | 298.9 | 56.7 | -92.2 |
| hurs_range | 33.5 | 7.56 | 2.18 |
| pet_penman_mean | 176.5 | 121.68 | 75.61 |
| vpd_mean | 1671 | 1024.4 | 421.1 |
| npp | 2607 | 2213.8 | 1253.6 |
| rsds_mean | 22.225 | 17.14 | 12.804 |
| rsds_range | 13.669 | 5.71 | 1.915 |
| phh2o | 74.33334 | 50.33333 | 40 |
| silt | 612.6667 | 249.6667 | 53 |
| soc | 1538 | 285.6667 | 164.6667 |
| wv0010 | 503 | 384 | 313.6667 |
| cec | 474.2017 | 136.1372 | 72.33551 |
| clay | 494.1141 | 342.1399 | 187.425 |
| nitrogen | 689.6667 | 216 | 127.3333 |
| dxx | 0.000668 | -1.2E-06 | -0.00074 |
| northness | 0.801581 | 0.027777 | -0.75476 |
| roughness | 387.25 | 27.6875 | 0 |
| tcurv | 0.000968 | 6.22E-06 | -0.00069 |
| tpi | 33.875 | -0.03906 | -22.2109 |
| vrm | 0.037944 | 0.000417 | 0 |

**Table S9. Category, description and short name of 69 environmental variables**

| Category | Varibale | Shortname |
| --- | --- | --- |
| Temperature | mean annual air temperature | bio01 |
|  | mean diurnal air temperature range | bio02 |
|  | isothermality | bio03 |
|  | temperature seasonality | bio04 |
|  | mean daily maximum air temperature of the warmest month | bio05 |
|  | mean daily minimum air temperature of the coldest month | bio06 |
|  | annual range of air temperature | bio07 |
|  | mean daily mean air temperatures of the wettest quarter | bio08 |
|  | mean daily mean air temperatures of the driest quarter | bio09 |
|  | mean daily mean air temperatures of the warmest quarter | bio10 |
|  | mean daily mean air temperatures of the coldest quarter | bio11 |
| Precipitation | annual precipitation amount | bio12 |
|  | precipitation amount of the wettest month | bio13 |
|  | precipitation amount of the driest month | bio14 |
|  | precipitation seasonality | bio15 |
|  | mean monthly precipitation amount of the wettest quarter | bio16 |
|  | mean monthly precipitation amount of the driest quarter | bio17 |
|  | mean monthly precipitation amount of the warmest quarter | bio18 |
|  | mean monthly precipitation amount of the coldest quarter | bio19 |
|  | aridity Index | ai |
|  | Mean monthly climate moisture index | cmi_mean |
|  | Annual range of monthly climate moisture index | cmi_range |
|  | Mean monthly near-surface relative humidity | hurs_mean |
|  | Annual range of monthly nearsurface relative humidity | hurs_range |
|  | Mean monthly potential evapotranspiration | pet_penman_mean |
|  | Annual range of monthly potential evapotranspiration | pet_penman_range |
|  | Mean monthly vapor pressure deficit | vpd_mean |
|  | Annual range of monthly vapor pressure deficit | vpd_range |
| Grow season | Growing degree days heat sum above 0°C | gdd0 |
|  | Growing degree days heat sum above 5°C | gdd5 |
|  | Growing degree days heat sum above 10°C | gdd10 |
|  | growing season length TREELIM | gsl |
|  | Accumulated precipiation amount on growing season days TREELIM | gsp |
|  | Mean temperature of the growing season TREELIM | gst |
|  | Net primary productivity | npp |
| Solar radiation | Mean monthly surface downwelling shortwave flux in air | rsds_mean |
|  | Annual range of monthly surface downwelling shortwave flux in air | rsds_range |
| Soil | Bulk density of the fine earth fraction | bdod |
|  | Cation Exchange Capacity of the soil | cec |
|  | Volumetric fraction of coarse fragments (> 2 mm) | cfvo |
|  | Proportion of clay particles (< 0.002 mm) in the fine earth fraction | clay |
|  | Total nitrogen (N) | nitrogen |
|  | Organic carbon density | ocd |
|  | Organic carbon stocks | ocs |
|  | Soil pH | phh2o |
|  | Proportion of sand particles (> 0.05 mm) in the fine earth fraction | sand |
|  | Proportion of silt particles (≥ 0.002 mm and ≤ 0.05 mm) in the fine earth fraction | silt |
|  | Soil organic carbon content in the fine earth fraction | soc |
|  | Volumetric Water Content at 10kPa in 10-3 cm3cm-3 | wv0010 |
| Topography | Aspect Cosine | aspectcosine |
|  | aspectsine | aspectsine |
|  | First order partial derivative (E-W slope) | dx |
|  | Second order partial derivative (E-W slope) | dxx |
|  | First order partial derivative (N-S slope) | dy |
|  | Second order partial derivative (N-S slope) | dyy |
|  | Eastness | eastness |
|  | Elevation | elevation |
|  | Northness | northness |
|  | Profile curvature | pcurv |
|  | Roughness | roughness |
|  | Slope | slope |
|  | Tangential curvature | tcurv |
|  | Topographic Position Index | tpi |
|  | Terrain Ruggedness Index | tri |
|  | Vector Ruggedness Measure | vrm |
| Geography | Area of geographic regions km^2^ | area_TDWG |
|  | Surrounding landmass proportion: Summed proportions of landmass area surrounding the target region within buffer distances of 10,000 km | SLMP_10 |
|  | Surrounding landmass proportion: Summed proportions of landmass area surrounding the target region within buffer distances of 1000 km | SLMP_1 |
| Past environments | Floristic kingdoms: Antarctic kingdom, Australis kingdom, Cape kingdom, Holarctic kingdom, Neotropic kingdom, Paleotropic kingdom. | Kingdom |

**Appendices**

Data S1 (separate file). Shumei Xiao. 2024. (Date the data were deposited: 2024-11-14). Tree species present in 429 pantropical tree inventory plots. DOI: 10.6084/m9.figshare.27677763. Available at: https://figshare.com/s/7bfdbce44102ae5930e8.

Data S2 (separate file) Shumei Xiao. 2024. (Date the data were deposited: 2024-11-14). Contributors of 429 tree inventory plots. DOI: 10.6084/m9.figshare.27678141. Available at: https://figshare.com/s/eb715edfca7fa5aa97af.

Data S3 (separate file) Shumei Xiao. 2024. (Date the data were deposited: 2024-11-14). Source and full name of 69 environmental variables. DOI: 10.6084/m9.figshare.27678000. Available at: https://figshare.com/s/dd05174d59ff61bb28a8.

Data S4 (separate file) Shumei Xiao. 2024. (Date the data were deposited: 2024-11-14). Sample richness and environmental data. DOI: 10.6084/m9.figshare.27683355. Available at: https://figshare.com/s/df0bb170c33e8772990c.

Data S5 (separate file) Shumei Xiao. 2024. (Date the data were deposited: 2024-11-14). Environmental data used for prediction in random forest analysis. DOI: 10.6084/m9.figshare.27678477. Available at: https://figshare.com/s/3eb0e9ea89173b4f894a.

Data S6 (separate file) Shumei Xiao. 2024. (Date the data were deposited: 2024-11-14). The result of Boruta algorithm selecting environment variables. DOI: 10.6084/m9.figshare.27715092. Available at: https://figshare.com/s/d6acd555184a2bb05aaf.

Data S7 (separate file) Shumei Xiao. 2024. (Date the data were deposited: 2024-11-14). R script used in this study. DOI: 10.6084/m9.figshare.27715089. Available at: https://figshare.com/s/2807afc99a601c162894.

**References**

[1] Gotelli NJ, Colwell RK. Quantifying biodiversity: procedures and pitfalls in the measurement and comparison of species richness. Ecology Letters 2001;4:379–91.

[2] Slik JWF, Arroyo-Rodriguez V, Aiba S-I and others. An estimate of the number of tropical tree species. Proceedings of the National Academy of Sciences 2015. https://doi.org/10/f7gq67.

[3] Chao A, Jost L. Coverage‐based rarefaction and extrapolation: standardizing samples by completeness rather than size. Ecology 2012;93:2533–47. https://doi.org/10.1890/11-1952.1.

[4] T. C. Hsieh, K. H. Ma and Anne Chao. iNEXT: Interpolation and Extrapolation for Species Diversity 2015:3.0.1. https://doi.org/10.32614/CRAN.package.iNEXT.

[5] Fisher RA, Corbet AS, Williams CB. The relation between the number of species and the number of individuals in a random sample of an animal population. The Journal of Animal Ecology 1943:42–58.

[6] Cazzolla Gatti R, Reich PB, Gamarra JGP, Crowther T, Hui C, Morera A, et al. The number of tree species on Earth. Proceedings of the National Academy of Sciences 2022;119:e2115329119. https://doi.org/10/gpbnf6.

[7] Ter Steege H, Pitman NCA, Do Amaral IL, De Souza Coelho L, De Almeida Matos FD, De Andrade Lima Filho D, et al. Mapping density, diversity and species-richness of the Amazon tree flora. Commun Biol 2023;6:1130. https://doi.org/10.1038/s42003-023-05514-6.

[8] Cooper DLM, Lewis SL, Sullivan MJP, Prado PI, Ter Steege H, Barbier N, et al. Consistent patterns of common species across tropical tree communities. Nature 2024;625:728–34. https://doi.org/10.1038/s41586-023-06820-z.

[9] Oksanen J, Simpson GL, Blanchet FG, Kindt R, Legendre P, Minchin PR, et al. vegan: Community Ecology Package 2024.

[10] Wright MN, Ziegler A. ranger: A Fast Implementation of Random Forests for High Dimensional Data in C++ and R. J Stat Soft 2017;77. https://doi.org/10.18637/jss.v077.i01.

[11] Benito B. SpatialRF: easy spatial regression with random forest. R Package Version 2021;1.

[12] Dray S, Legendre P, Peres-Neto PR. Spatial modelling: a comprehensive framework for principal coordinate analysis of neighbour matrices (PCNM). Ecological Modelling 2006;196:483–93. https://doi.org/10.1016/j.ecolmodel.2006.02.015.

[13] Hengl T, Nussbaum M, Wright MN, Heuvelink GBM, Gräler B. Random forest as a generic framework for predictive modeling of spatial and spatio-temporal variables. PeerJ 2018;6:e5518. https://doi.org/10.7717/peerj.5518.

[14] Ripley B, others. MASS: support functions and datasets for Venables and Ripley’s MASS. R Package Version 2011;7:3–29.

[15] Bates D, Mächler M, Bolker B, Walker S. Fitting Linear Mixed-Effects Models Using **lme4**. J Stat Soft 2015;67. https://doi.org/10.18637/jss.v067.i01.
