# Supplementary figures and images for: Analogous environments across the tropics have similar levels of tree species alpha diversity
Source: Natl Sci Rev. 2025 Oct 29;13(2):nwaf465. doi: 10.1093/nsr/nwaf465 (PMC12860206; doi:10.1093/nsr/nwaf465)

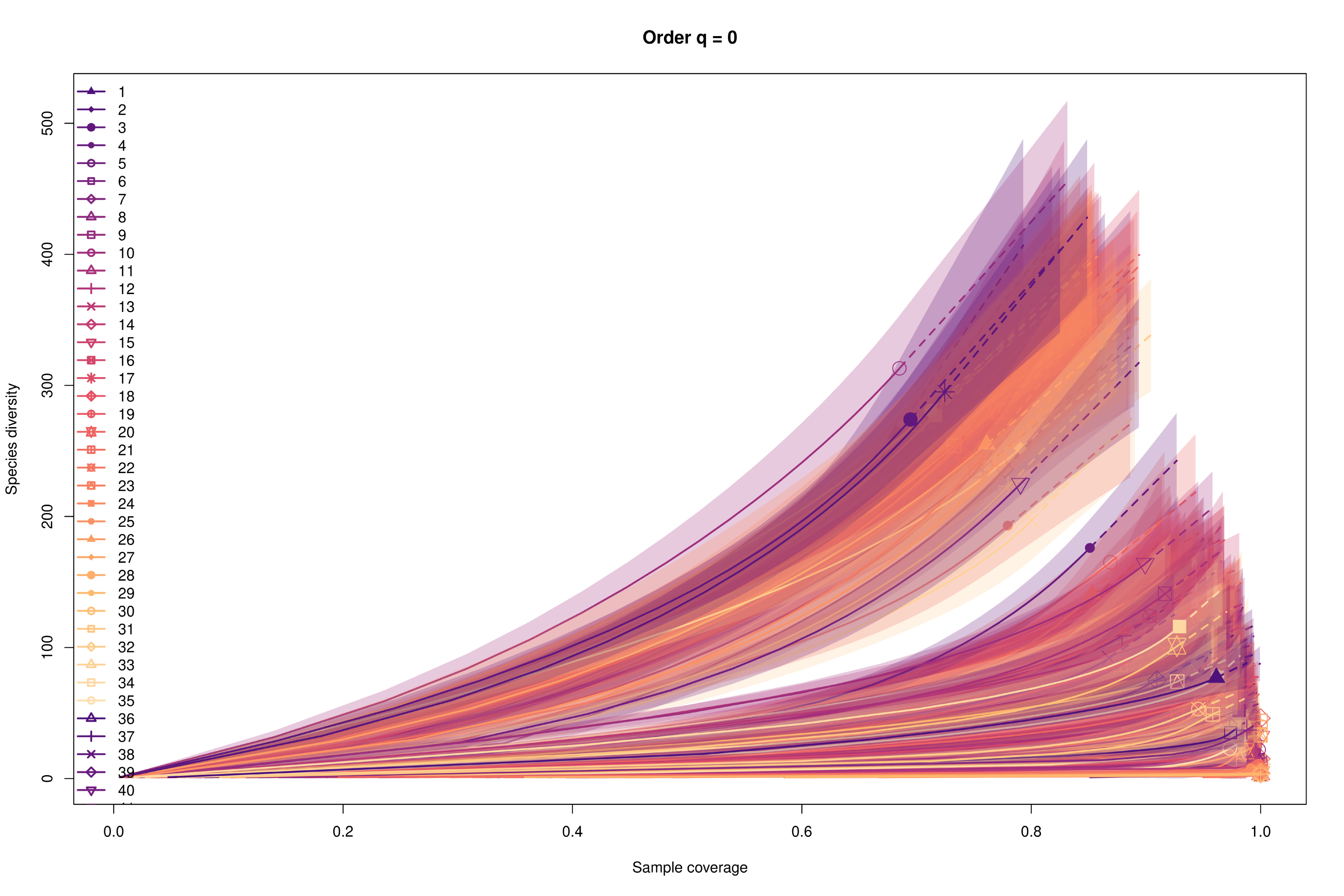

Supplement: nwaf465_Supplemental_Files [file nwaf465_supplemental_files.zip › Supplementary_files/Fig. S1.tif]

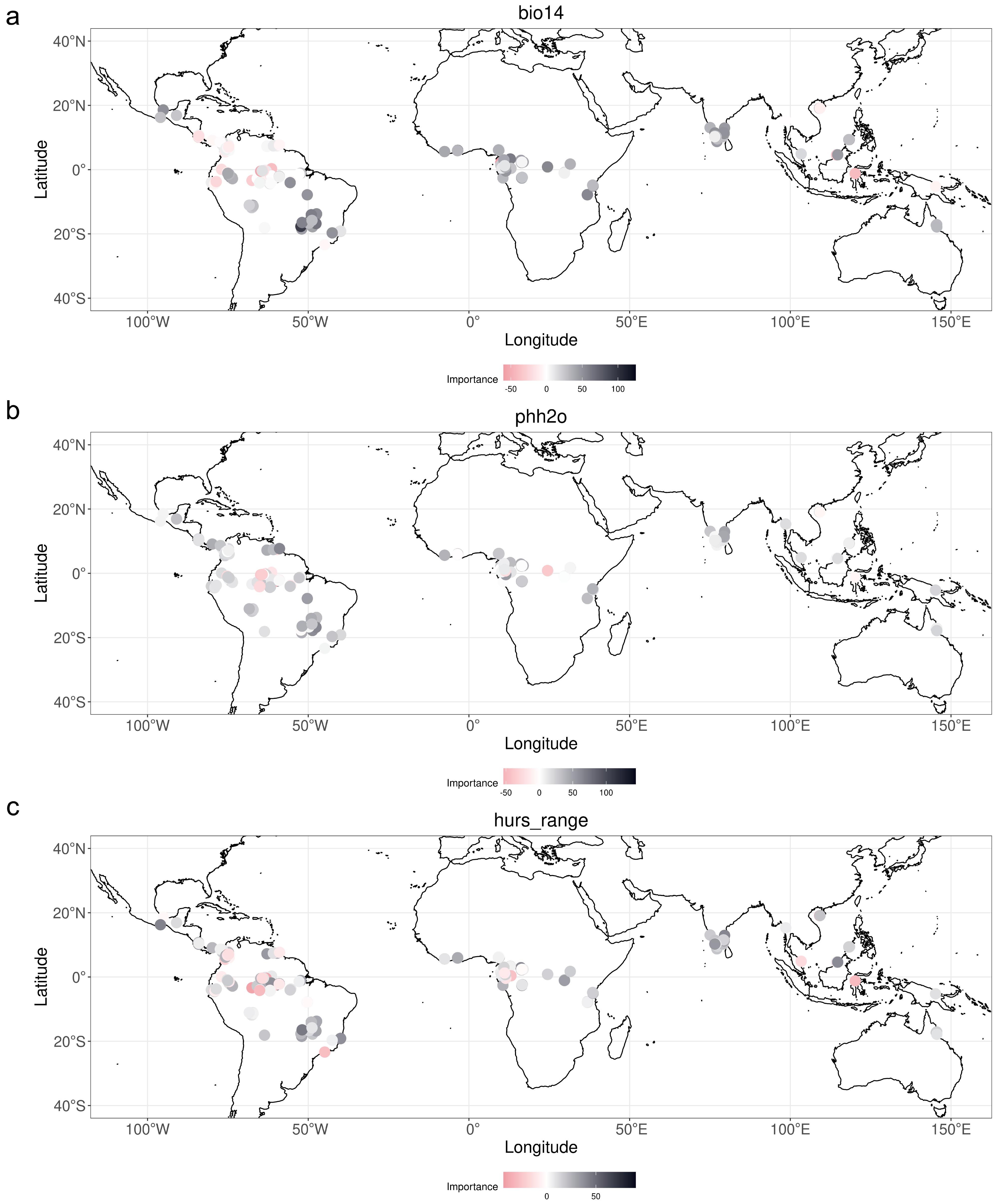

Supplement: nwaf465_Supplemental_Files [file nwaf465_supplemental_files.zip › Supplementary_files/Fig. S12.tif]

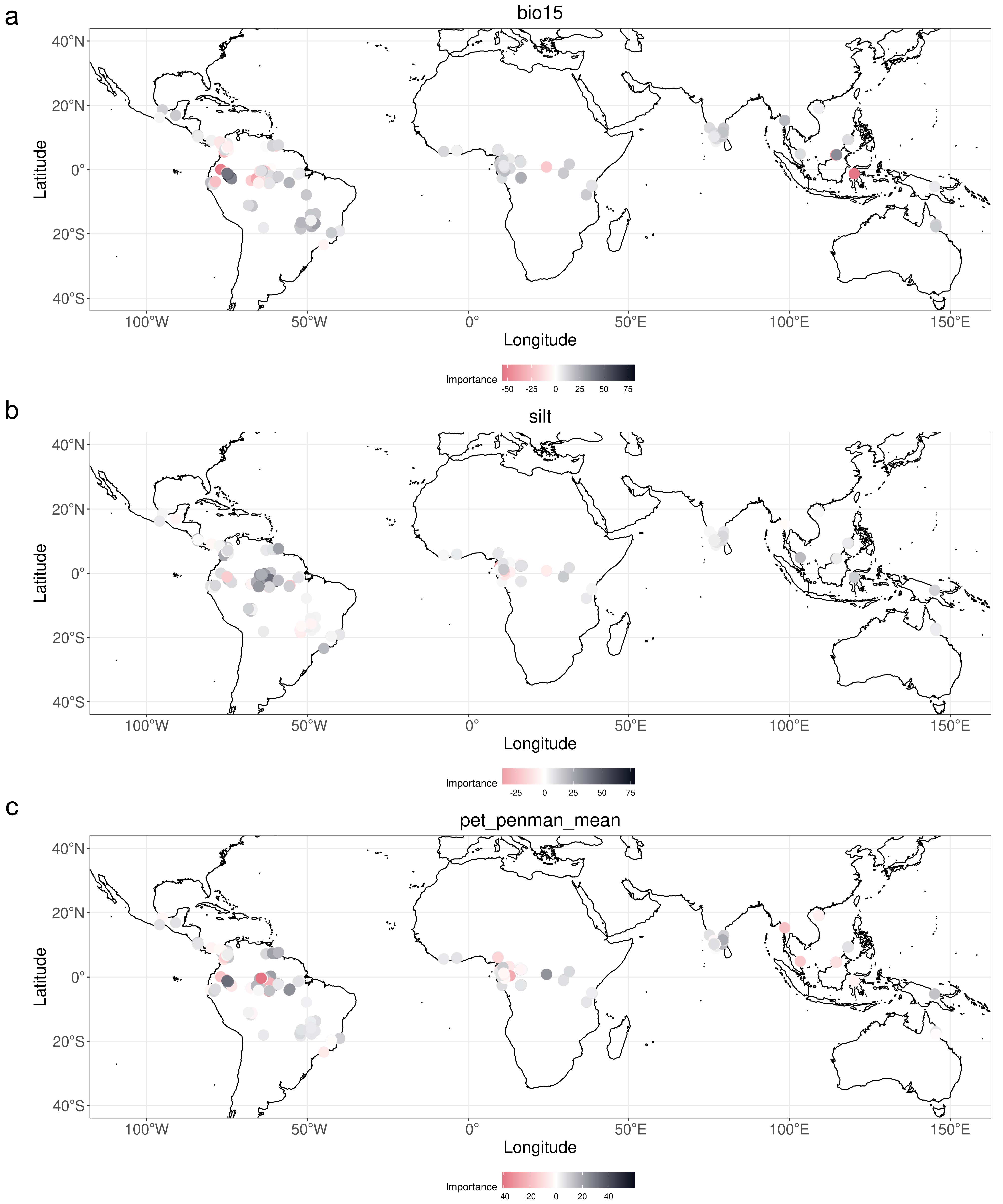

Supplement: nwaf465_Supplemental_Files [file nwaf465_supplemental_files.zip › Supplementary_files/Fig. S13.tif]

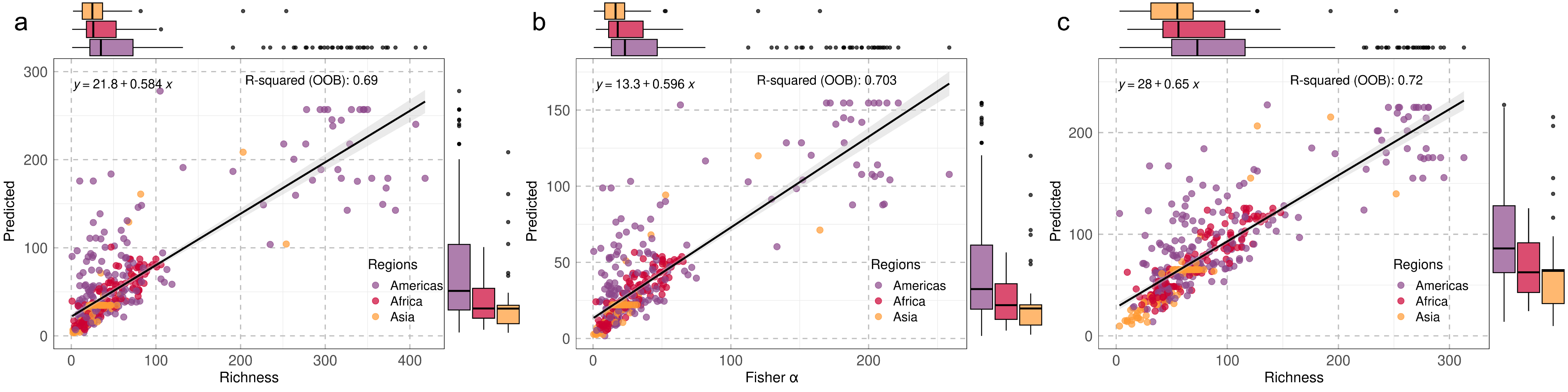

Supplement: nwaf465_Supplemental_Files [file nwaf465_supplemental_files.zip › Supplementary_files/Fig. S14.tif]

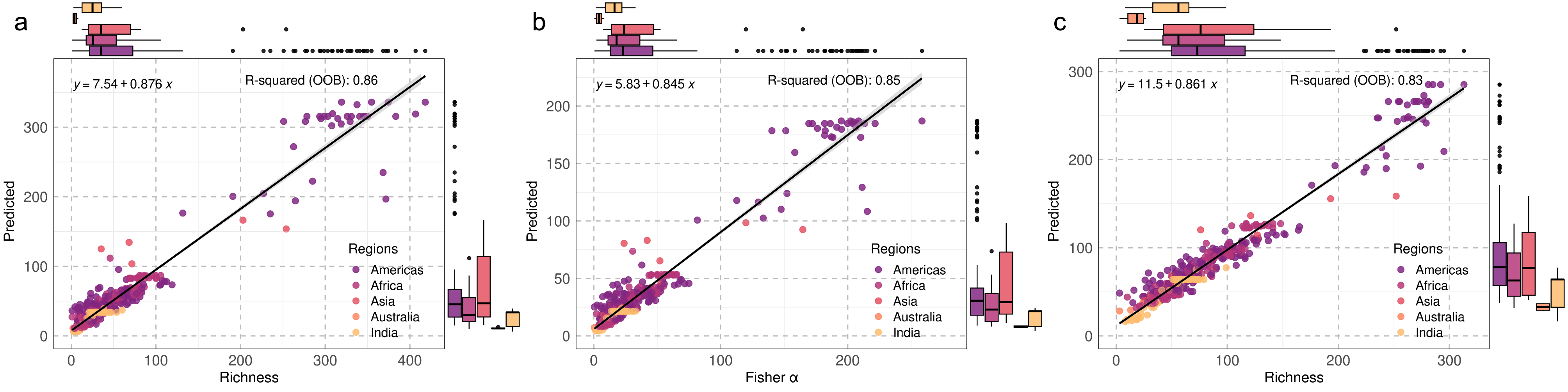

Supplement: nwaf465_Supplemental_Files [file nwaf465_supplemental_files.zip › Supplementary_files/Fig. S15.tif]

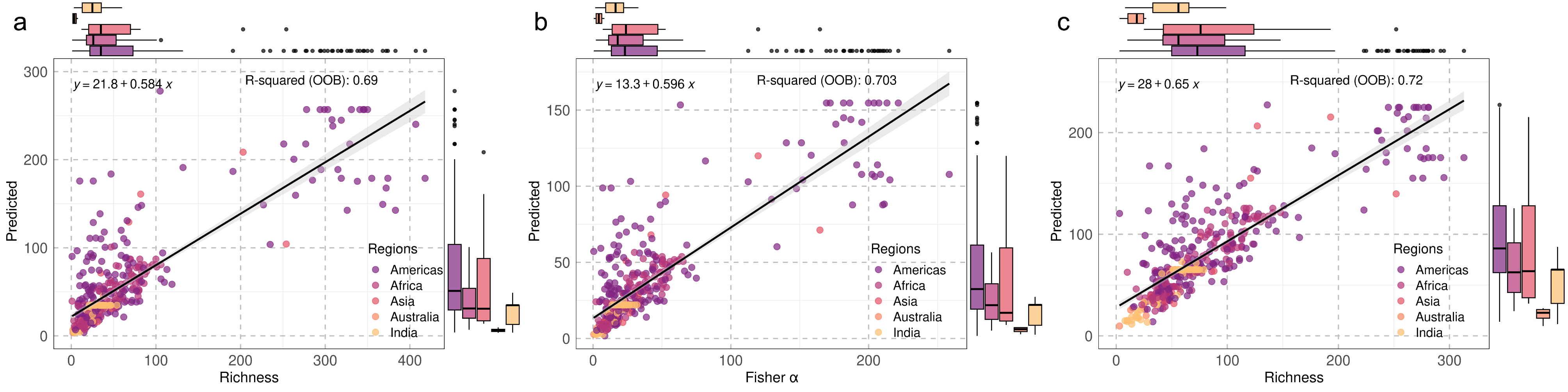

Supplement: nwaf465_Supplemental_Files [file nwaf465_supplemental_files.zip › Supplementary_files/Fig. S16.tif]

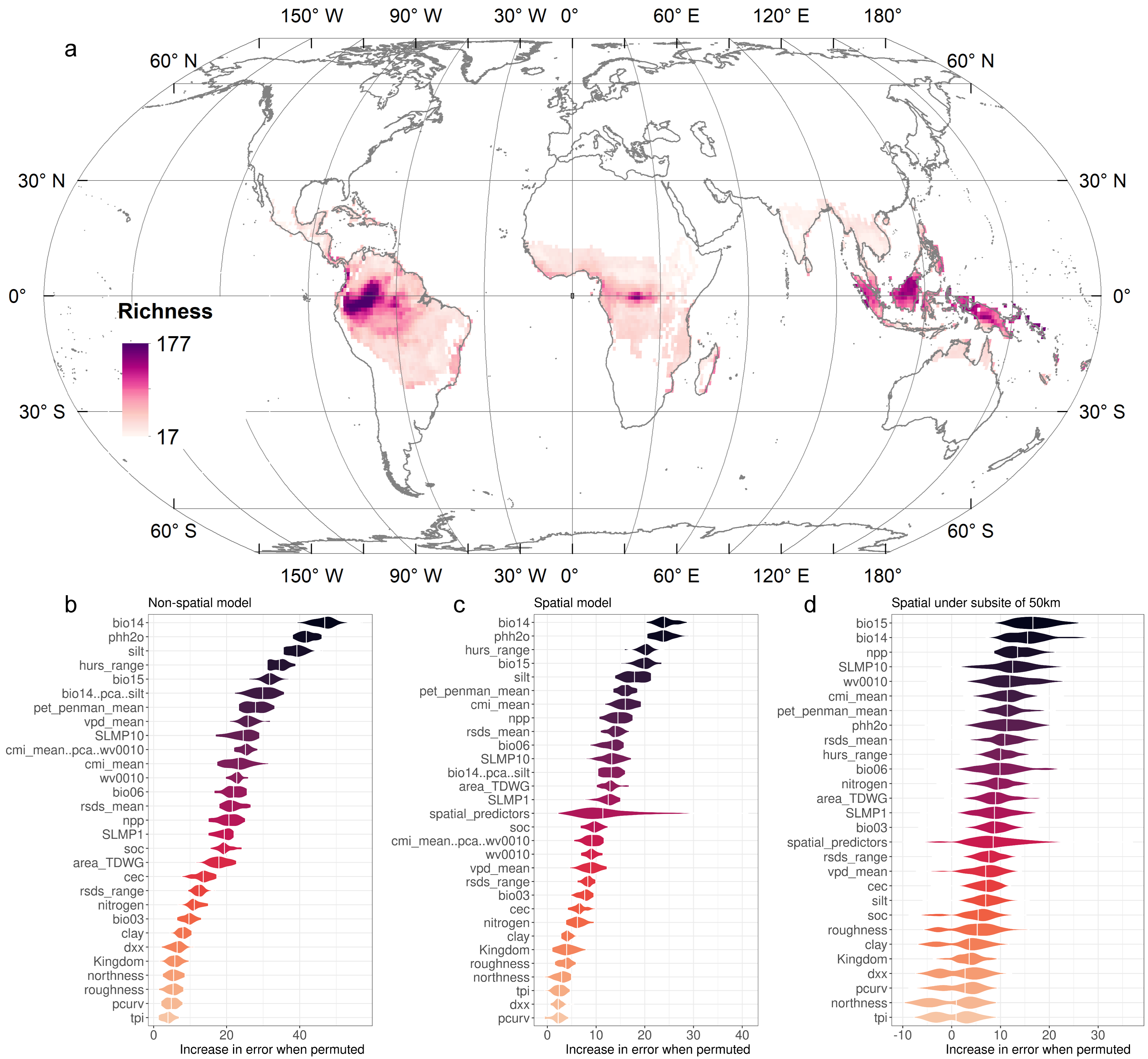

Supplement: nwaf465_Supplemental_Files [file nwaf465_supplemental_files.zip › Supplementary_files/Fig. S17.tif]

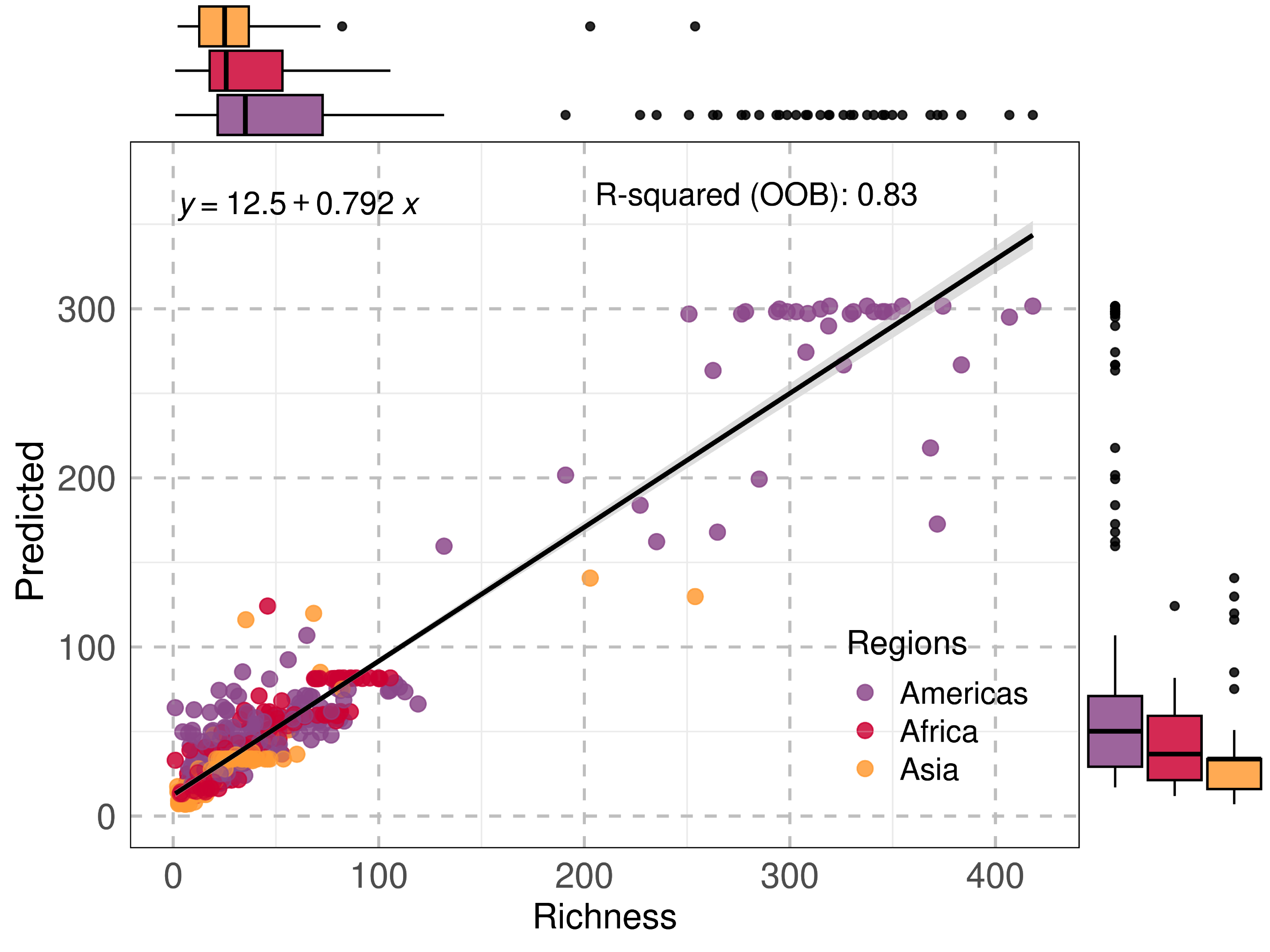

Supplement: nwaf465_Supplemental_Files [file nwaf465_supplemental_files.zip › Supplementary_files/Fig. S18.tif]

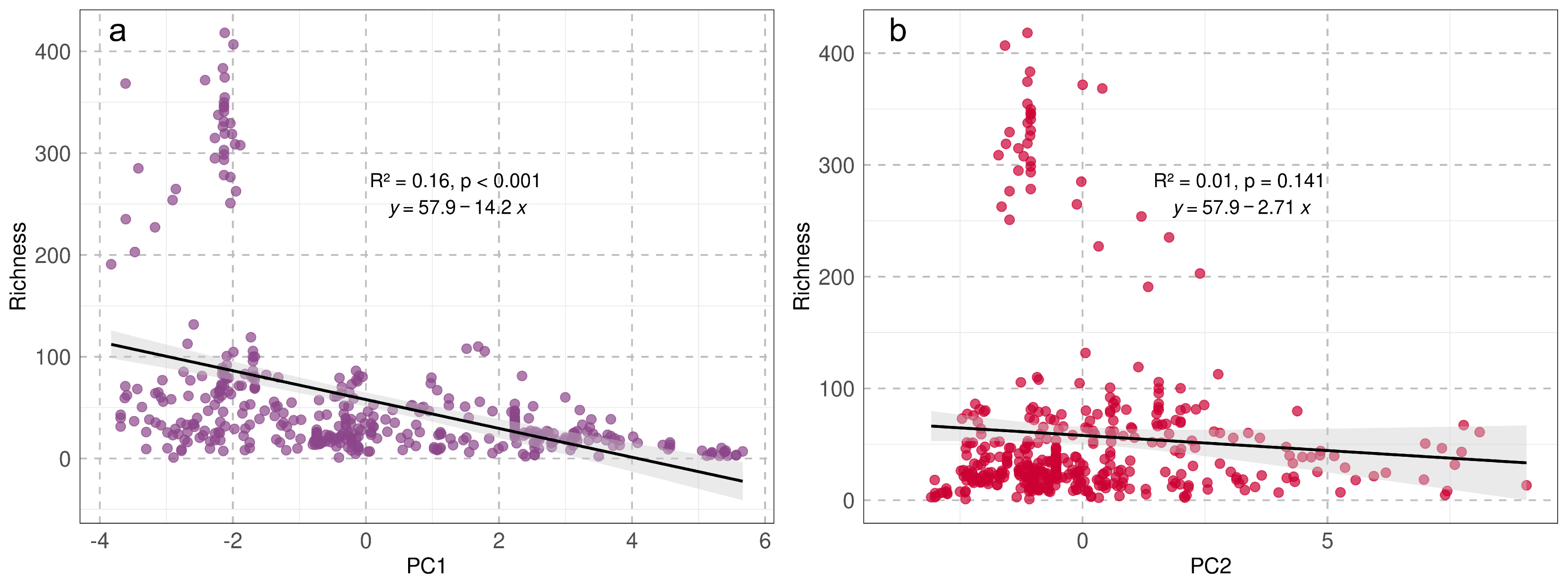

Supplement: nwaf465_Supplemental_Files [file nwaf465_supplemental_files.zip › Supplementary_files/Fig. S19.tif]

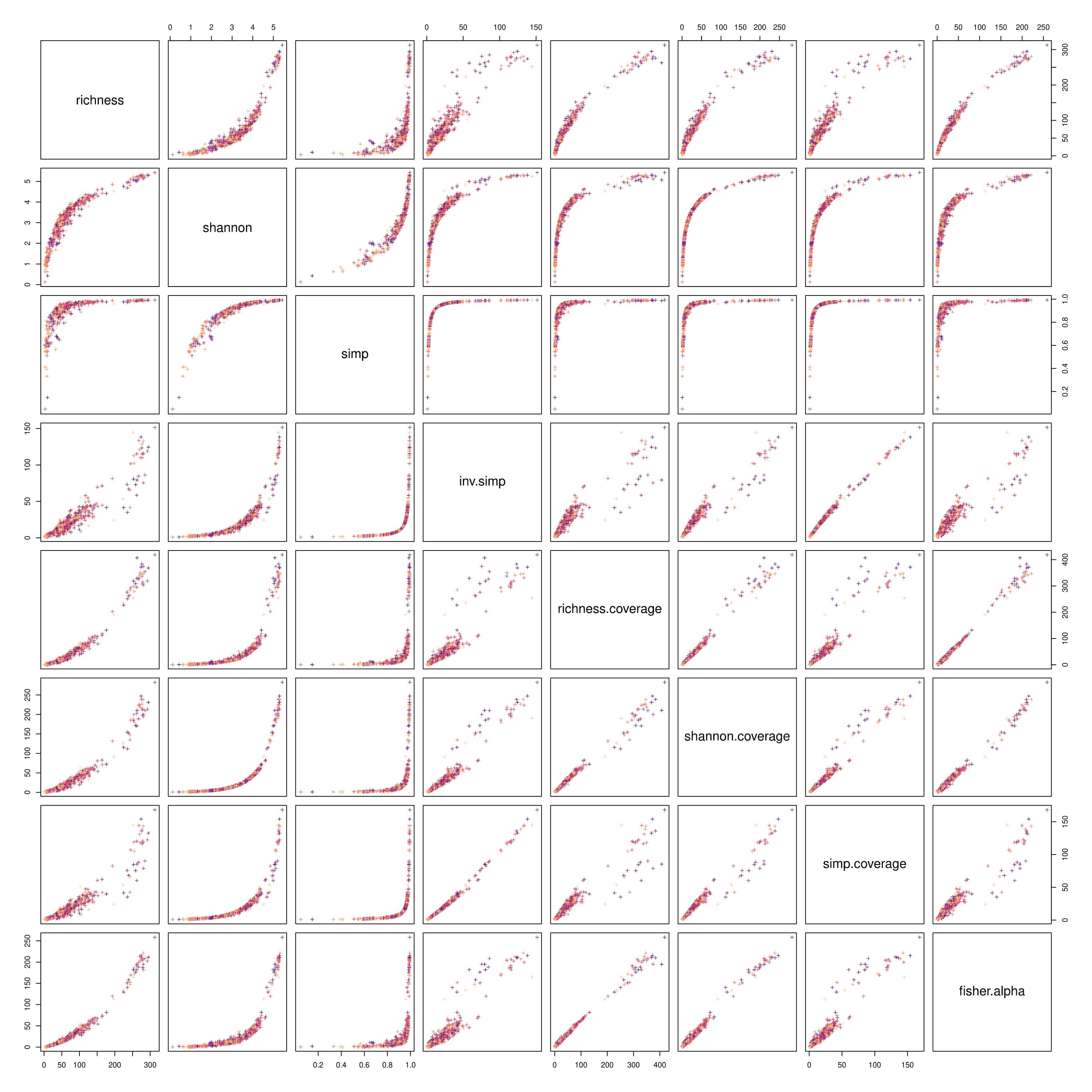

Supplement: nwaf465_Supplemental_Files [file nwaf465_supplemental_files.zip › Supplementary_files/Fig. S2.tif]

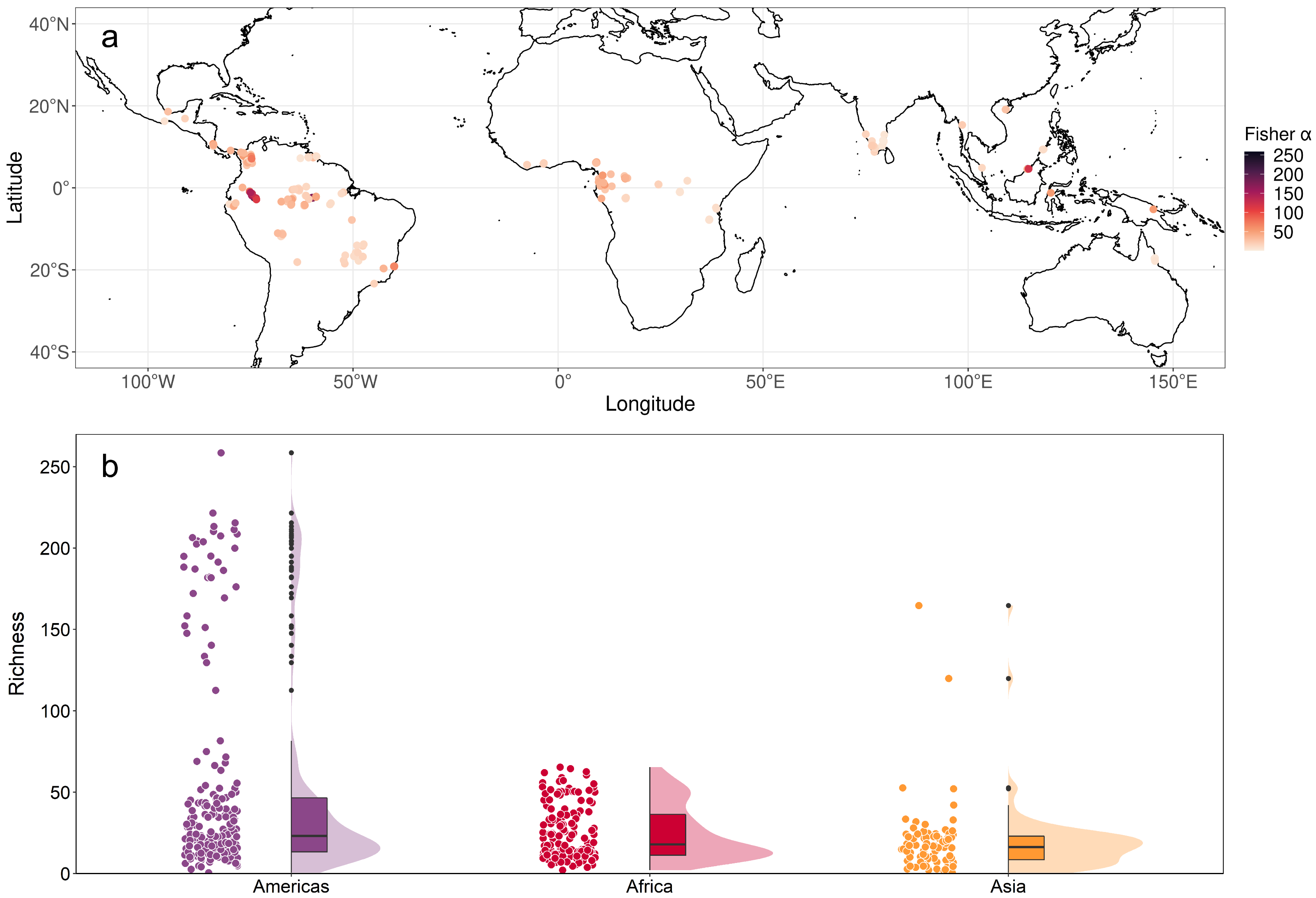

Supplement: nwaf465_Supplemental_Files [file nwaf465_supplemental_files.zip › Supplementary_files/Fig. S3.tif]

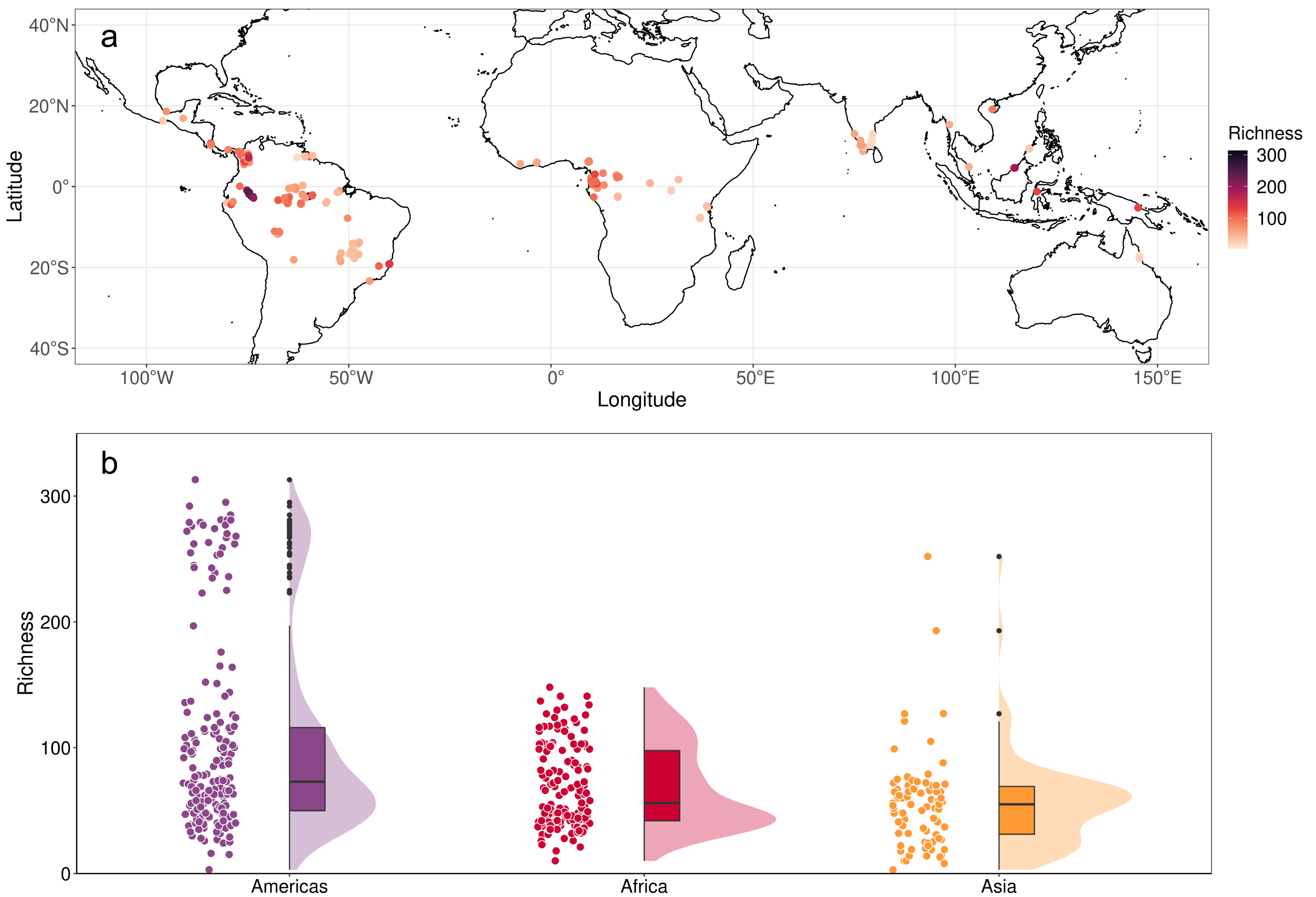

Supplement: nwaf465_Supplemental_Files [file nwaf465_supplemental_files.zip › Supplementary_files/Fig. S4.tif]

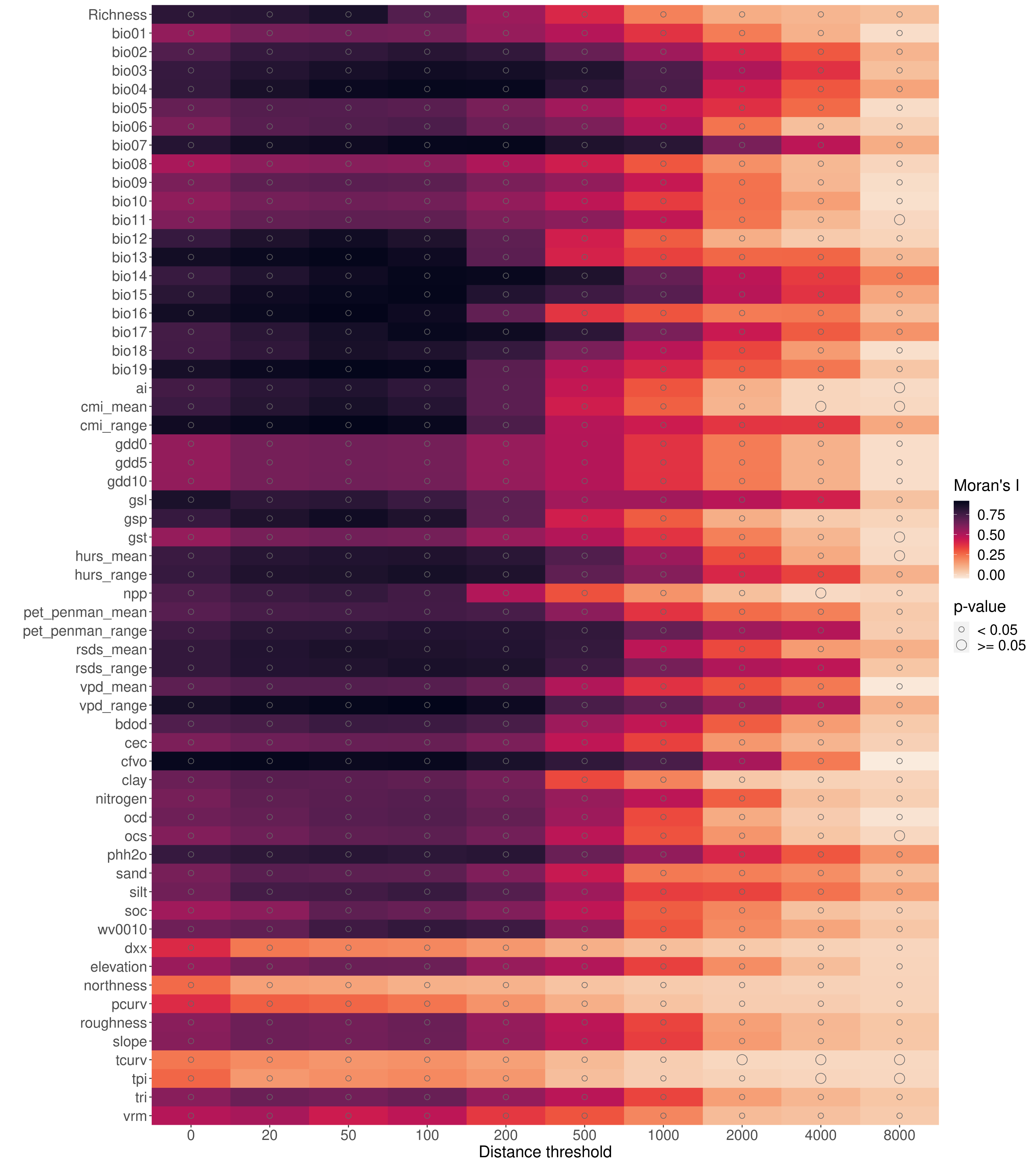

Supplement: nwaf465_Supplemental_Files [file nwaf465_supplemental_files.zip › Supplementary_files/Fig. S5.tif]

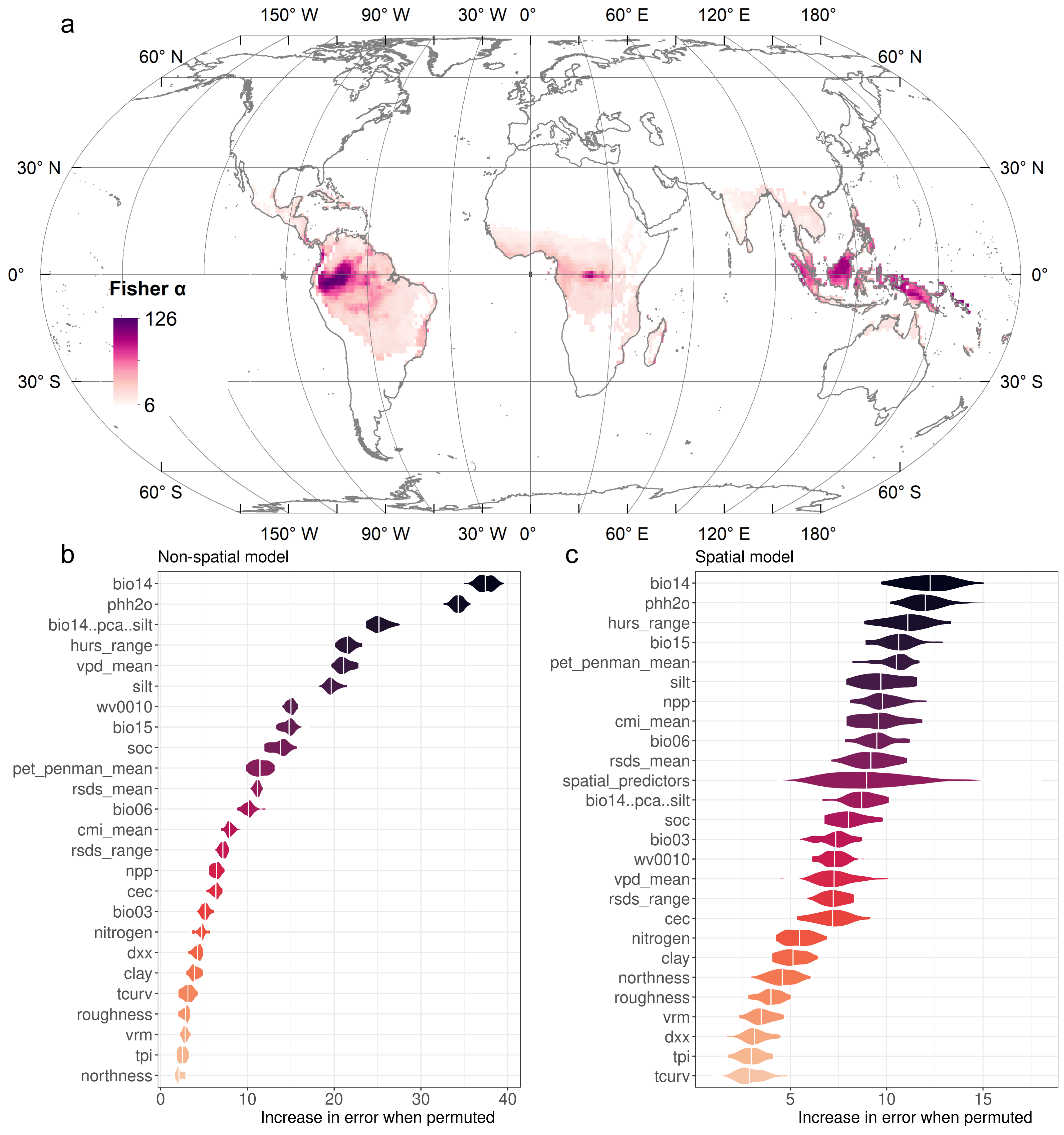

Supplement: nwaf465_Supplemental_Files [file nwaf465_supplemental_files.zip › Supplementary_files/Fig. S6.tif]

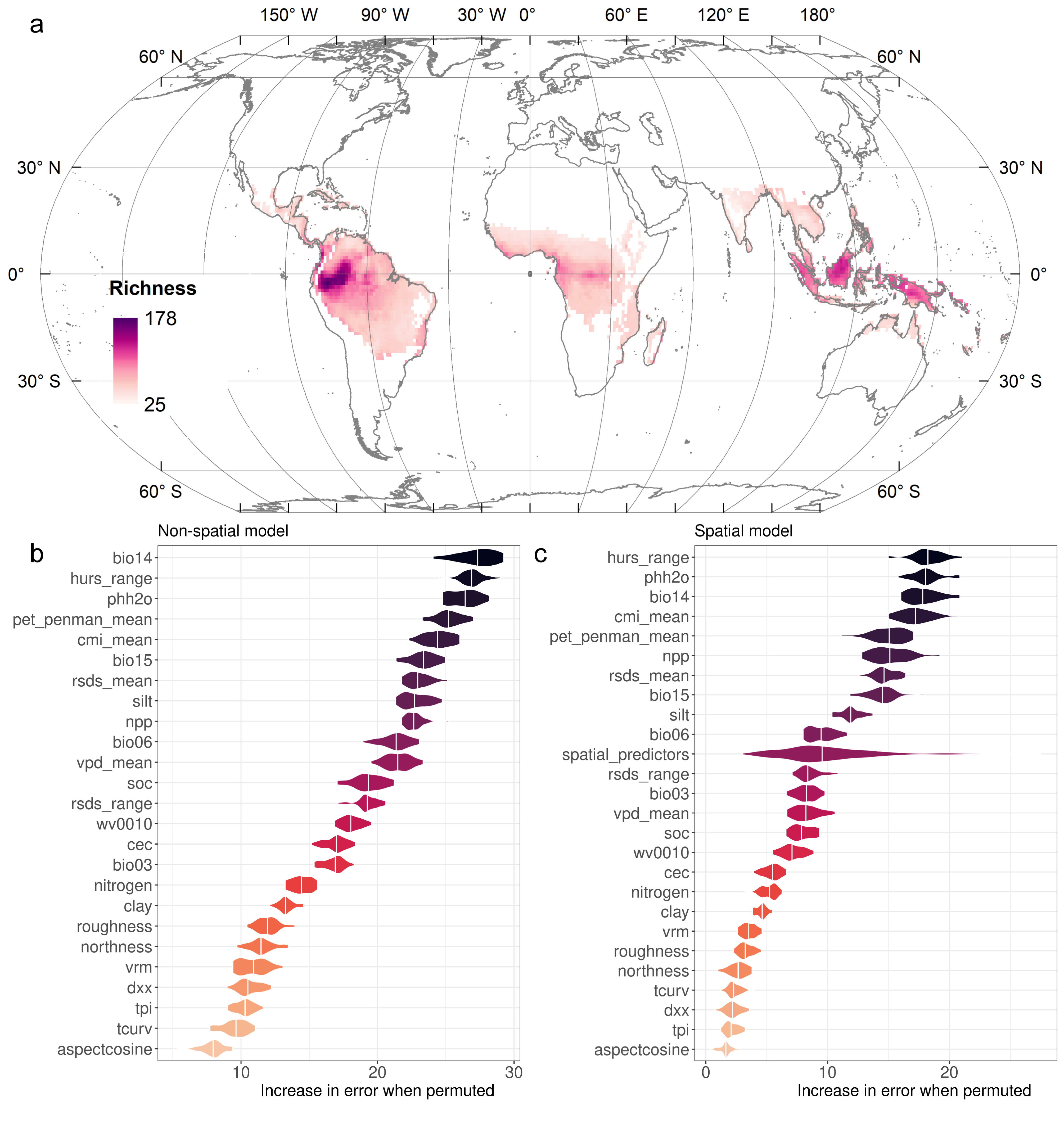

Supplement: nwaf465_Supplemental_Files [file nwaf465_supplemental_files.zip › Supplementary_files/Fig. S7.tif]

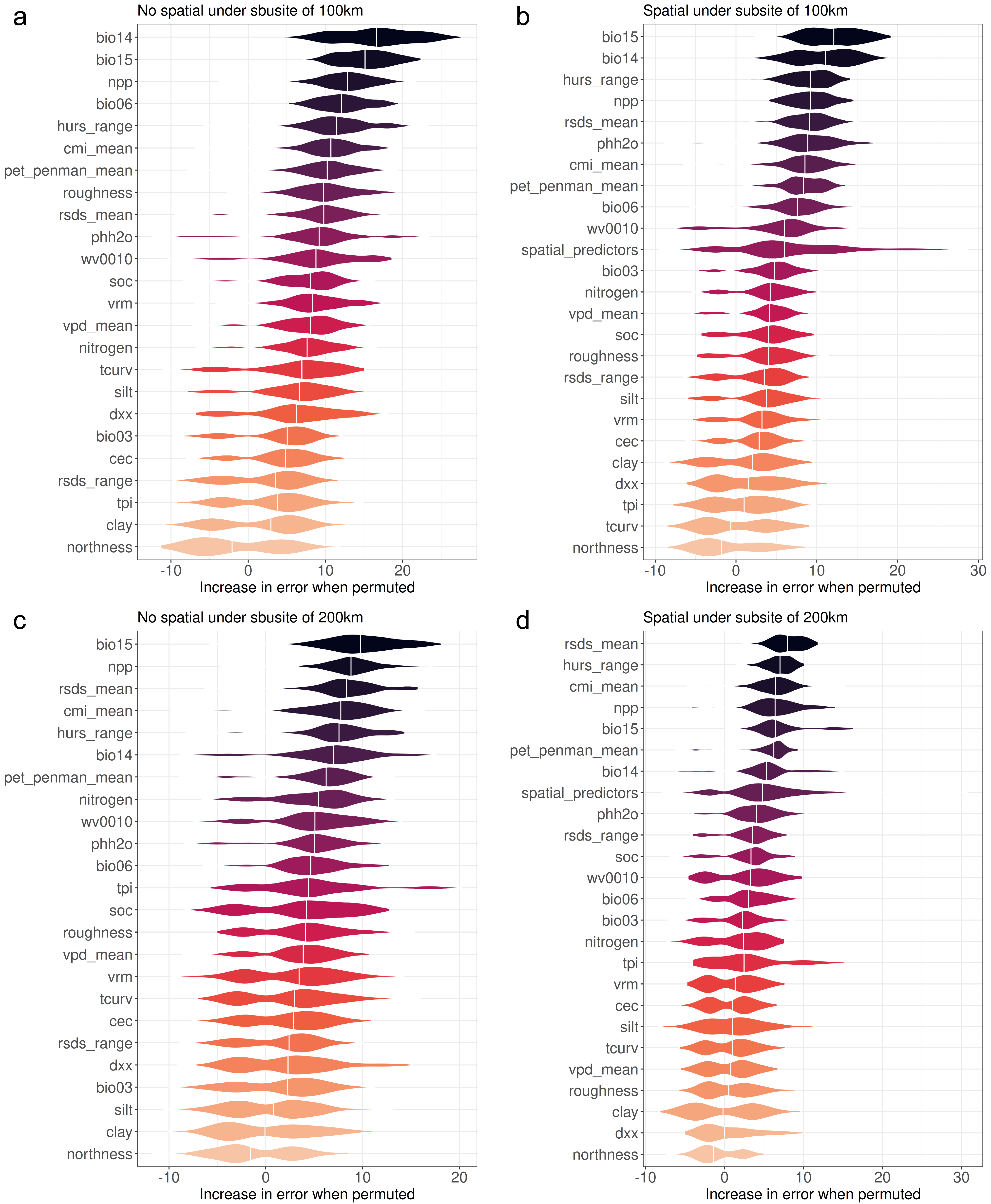

Supplement: nwaf465_Supplemental_Files [file nwaf465_supplemental_files.zip › Supplementary_files/Fig. S8.tif]

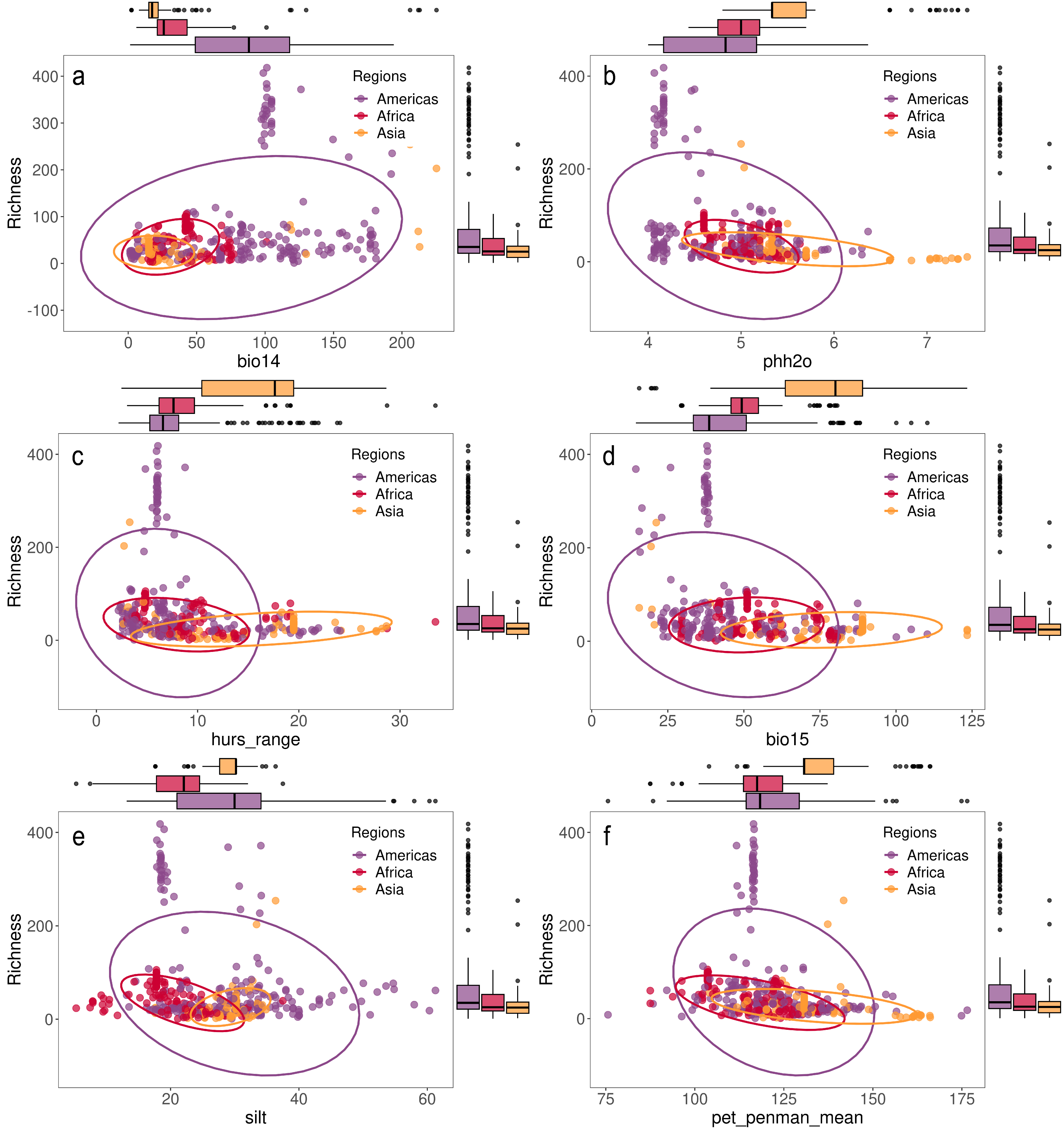

Supplement: nwaf465_Supplemental_Files [file nwaf465_supplemental_files.zip › Supplementary_files/Fig. S9.tif]
